# Restoring Iron Homeostasis via Smoothened Inhibition: A Novel Strategy Against Hearing Loss

**DOI:** 10.1002/advs.202520749

**Published:** 2026-04-17

**Authors:** Huanyu Mao, Xiang Li, Yaqi Liao, Liman Liu, Xian Gao, Hailiang Lin, Wenli Ni, Huawei Li, Yan Chen, Wenyan Li

**Affiliations:** ^1^ ENT Institute and Otorhinolaryngology Department of Eye & ENT Hospital State Key Laboratory of Medical Neurobiology and MOE Frontiers Center For Brain Science Institutes of Biomedical Sciences Fudan University Shanghai P. R. China; ^2^ NHC Key Laboratory of Hearing Medicine (Fudan University) Shanghai P. R. China; ^3^ Department of Otorhinolaryngology Head and Neck Surgery Wuhan Third Hospital Tongren Hospital of Wuhan University Wuhan P. R. China; ^4^ Department of Surgery–Otolaryngology Yale University Medical School New Haven Connecticut USA; ^5^ The Institutes of Brain Science and the Collaborative Innovation Center for Brain Science Fudan University Shanghai P. R. China

**Keywords:** ferroptosis, hair cells preservation, hearing loss, hedgehog signaling, iron homeostasis, smoothened

## Abstract

Sensorineural hearing loss (SNHL) induced by noise or aminoglycoside antibiotics is a significant public health concern without any FDA‐approved pharmaceutical therapies. Dysregulation of iron homeostasis and its subsequently induced ferroptosis has increasingly been identified as a key mechanism underlying cochlear hair cell (HC) damage. Nevertheless, the therapeutic targets for restoring iron balance for hearing protection remain poorly investigated. In this study, we uncover a previously unrecognized role of the SMO pathway in regulating iron homeostasis. SMO expression was rapidly upregulated and activated in HCs following injury. Both genetic ablation of *Smo* and pharmacological inhibition of SMO reduced iron accumulation and lipid peroxidation, promoting HC survival and preserving auditory function in mouse models of ototoxic‐ and noise‐induced hearing loss. Mechanistically, SMO inhibition suppressed ATF2 phosphorylation, resulting in downregulation of IRP1, which decreased iron accumulation via downregulation of *Tfrc* and upregulation of *Fpn*, ultimately protecting HCs from ferroptosis. Notably, treatment with the SMO inhibitor SANT‐1 nearly restored auditory thresholds to baseline levels in mice subjected to ototoxic injury. Our findings identify the SMO–ATF2–IRP1–FPN/TFRC axis as a central regulator of cochlear iron homeostasis and propose SMO inhibition as a promising therapeutic strategy for SNHL through precise modulation of iron metabolism.

## Graphical abstract

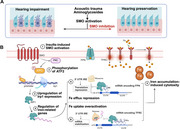

## 1 Introduction

Sensorineural hearing loss (SNHL) is one of the most common sensory disorders, affecting over 400 million people worldwide, with a steadily increasing incidence [1, 2]. Major contributors to SNHL include noise exposure and the use of aminoglycoside antibiotics. Despite its prevalence, no pharmacological therapies have been approved by the Food and Drug Administration (FDA), underscoring the urgent need for novel treatment strategies. Cochlear hair cells (HCs), the mechanosensory receptors of the auditory system, are highly susceptible to iron overload [3, 4], which catalyzes the production of reactive oxygen species (ROS) and lipid peroxidation, ultimately triggering ferroptosis [5]. Ferroptosis has been implicated in both noise‐ and drug‐induced hearing loss [4, 6], and systemic iron overload in conditions such as hemochromatosis is associated with auditory neuropathy [7], highlighting the critical importance of precise iron regulation in auditory protection.

Current approaches to mitigate iron‐related injury primarily rely on iron chelators, such as deferoxamine (DFO), deferiprone (DFP), and deferasirox (DFX), which function by binding circulating iron to reduce oxidative stress [8, 9]. However, their clinical use in hearing protection is constrained by dose‐dependent neurotoxicity and ototoxicity [10, 11], largely resulting from the disruption of physiological iron‐dependent processes, including mitochondrial respiration, DNA synthesis, and myelination [12]. Therefore, a major challenge remains: how to suppress iron‐mediated oxidative damage in cochlear HCs without inducing the harmful effects of iron depletion. At the cellular level, iron homeostasis is maintained through a dynamic balance of iron influx, iron efflux, and intracellular iron recycling. Disruption of these pathways can increase the susceptibility of HCs to ferroptosis [6, 13, 14, 15]. Nevertheless, the complete mechanisms underlying iron‐induced cochlear damage remain incompletely understood, underscoring the need for further investigation.

**FIGURE 1:**
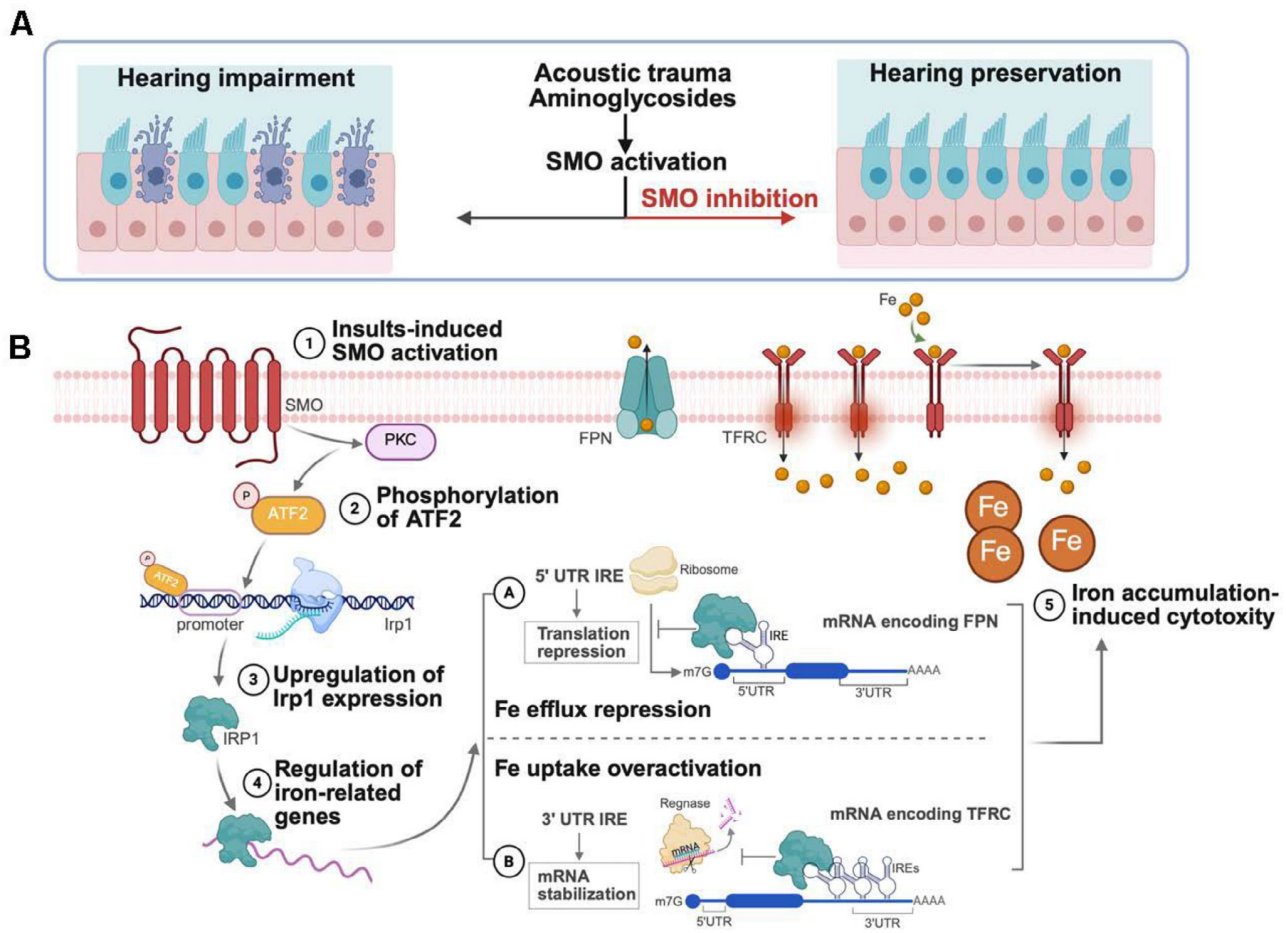
Diagram showing the otoprotective effects of SMO inhibition against aminoglycosides and noise‐induced HC death and hearing loss, and its underlying mechanism. (A) Ototoxic injuries‐induced aberrant activation of SMO signaling in cochlear HCs contributes to HCs' death and subsequent hearing impairment. Genetic ablation or pharmaceutical inhibition of SMO effectively ameliorated acoustic trauma‐ and aminoglycosides‐driven cochlear HC loss and sensorineural hearing impairment in mice. (B) Mechanistically, SMO activation phosphorylated ATF2 to increase IRP1 expression in a PKC‐dependent manner, which subsequently suppressed FPN‐mediated iron efflux and overactivated TFRC‐mediated iron influx, leading to aggravated deleterious iron accumulation and consequent iron‐dependent ferroptosis in HCs.

In this study, we systematically examined alterations in iron metabolism within injured cochlear HCs and identified a pathological disruption characterized mainly by markedly inhibited iron efflux, along with enhanced iron influx and ferritinophagy. Through integrated drug screening and transcriptomic analysis, we discovered that inhibitors of the smoothened (SMO) protein—a class of clinically approved antitumor agents—effectively restored iron homeostasis by promoting ferroportin (FPN)/ceruloplasmin (CP)‐mediated iron efflux and attenuating transferrin receptor protein C (TFRC)‐dependent iron influx. Notably, SMO expression was rapidly upregulated in cochlear HCs following injury. Based on these findings, we proposed that SMO inhibition could serve as a targeted strategy to reestablish iron homeostasis in HCs. Using both genetic and pharmacological approaches, we demonstrated that either *Smo* ablation or treatment with SMO inhibitors reduced iron accumulation and lipid peroxidation, thereby promoting HC survival and preserving auditory function in mouse models of aminoglycosides‐ and noise‐induced hearing loss. Mechanistically, SMO inhibition suppressed ATF2 phosphorylation, decreased IRP1 activity, and restored the expression of FPN and TFRC. Importantly, administration of the SMO‐specific inhibitor SANT‐1 restored auditory function to near‐baseline levels in an ototoxic model of hearing loss (Figure 1).

Collectively, our results identify the SMO–ATF2–IRP1–FPN/TFRC axis as a central regulatory pathway in cochlear iron homeostasis, establishing SMO inhibition as a promising therapeutic strategy for SNHL. These findings not only provide mechanistic insights into iron dysregulation in hearing loss but also underscore the translational potential of repurposing SMO inhibitors for precise auditory protection through iron modulation.

## 2 Materials and Methods

### 2.1 Animals

*Smo* ^2Amc^ (The Jackson Laboratory, 004526) and *Gfi1* ^Cre^ mice were crossed to produce *Gfi1* ^Cre^/*Smo* ^2Amc/2Amc^ mice (*Gfi1* ^Cre^/*Smo*KO mice). *Smo* ^2Amc^ and *Prestin* ^CreER^ mice were crossed to generate *Prestin* ^CreER^/*Smo* ^2Amc/2Amc^ mice (*Prestin* ^CreER^/*Smo*KO mice), which were injected with tamoxifen at a dose of 9 mg/40 g body weight intraperitoneally at P21 and P22 to drive *Smo* depletion in cochlear OHCs. *Smo* ^2Amc^, *Gfi1* ^Cre^ and *ROSA26* *^CAG‐tdTomato^* mice were crossed to generate *Gfi1* ^Cre^ */Smo* ^2Amc/2Amc^ */ROSA26^CAG‐tdTomato^* and *Gfi1* ^Cre^ */ROSA26^CAG‐tdTomato^* littermates, which were used for RNA sequencing. *Gfi1* ^Cre^/*ROSA26^CAG‐tdTomato^* mice were used to label postnatal cochlear HCs with fluorescence. All transgenic mouse lines were on C57BL/6J background. Wild‐type C57BL/6 mice were used to establish the aminoglycoside and noise challenge model.

All animals were bred and fed in the animal facility (SPF) of Fudan University, Shanghai, China. All animal experiments were approved by the Shanghai Medical Laboratory Animal Management Committee (license number: 2009‐0082) and the Animal Ethics Committee of Fudan University, with ARRIVE guidelines followed.

### 2.2 Cochlear Explants Culturing and Drug Administration

The cochlear epithelia were dissected from P2 mice in cold phosphate buffer saline (PBS) with surrounding tissues removed, and then transferred to a sterile slide coated with Cell‐Tak (BD Bioscience). The cochlear explants were cultured in DMEM/F12 medium containing N2 (Life Technologies), B27 (Life Technologies), and ampicillin (Sangon) at 37°C, 5%CO_2_. The spiral ganglion neurons connected to HCs were carefully preserved and co‐cultured with cochlear epithelia when necessary. At least four independent cochleae were examined for each experimental condition.

After overnight incubation, the cochlear explants were stable for drug administration. The compounds used in this study included neomycin (Selleck; 0.2 mm for the experiment in Figure 3B, 1 mm for other neomycin‐challenged cochlear explants, and 2 mm for HEI‐OC1 cell experiments), gentamicin (Selleck; 0.2 mm), tobramycin (Selleck; 0.2 mm), amikacin (Selleck; 0.5 mm), kanamycin (Selleck; 0.5 mm), SAG (Selleck; 0.05–10 µm), SANT‐1 (Selleck; 10 and 20 µm), cyclopamine (Selleck; 10 and 20 µm), taladegib (Selleck; 10 and 20 µm), glasdegib (Selleck; 10 and 20 µm), sonidegib (Selleck; 10 and 20 µm), vismodegib (Selleck; 10 and 20 µm), RSL3 (Selleck; 5 µm), ferrostatin‐1 (Selleck; 10 µm), ammonium ferric citrate (FAC; Selleck; 0.1–10 mm), deferoxamine (DFO; Selleck; 100–500 µm), D4476 (MCE; 100 µm), H89 (Selleck; 20 µm), MK‐2206 (MCE; 5 µm), GSK180736A (Selleck; 5 µm), dactolisib (Selleck; 10 µm), dasatinib (Selleck; 20 µm), bisindolylmaleimide II (BisII; MCE; 10 µm), Go6976 (Selleck; 5 µm), Enzastaurin (Selleck; 5 µm), and Rottlerin (Selleck; 5 µm).

### 2.3 Cell Viability Assay

Cell viability of HEI‐OC1 cells was determined using the Cell Counting Kit‐8 (CCK‐8, Beyotime, C0039) according to the manufacturer's instructions. Briefly, cells were seeded in 96‐well plates at a density of approximately 5000 cells per well. Following the designated treatments, 10 µL of CCK‐8 reagent was added to each well containing 100 µL of culture medium. The plates were then incubated at 37°C for 1 h. The absorbance at 450 nm was measured using a microplate reader. The optical density (OD) values from treatment groups were normalized to those of the untreated control group (defined as 100% viability).

### 2.4 Quantitative PCR (qPCR) and Primers

cDNA was synthesized using 2 µg of total RNA extracted from cochlear explants (*n* = 8 cochlea per each group) or cochlear epithelia dissected from adult mice (*n* = 6 cochlea per each group), using the PrimeScript RT reagent Kit with gDNA Eraser (Takara). For qPCR analysis of cultured explants, each biological replicate (*n*) consisted of RNA pooled from eight independently treated cochlear explants. For noise exposure experiments, each biological replicate (*n*) consisted of RNA pooled from six cochleae of three independent mice. qPCR was conducted using the ABI 7500 RealTime PCR System (Applied Biosystems) and TB Green Premix Ex Taq (Takara). All primers used in this study are listed in Table S1. The data were from at least three independent experiments.

### 2.5 Immunostaining

HEI‐OC1 cells, cochlear explants, or tissues dissected from adult mice were fixed with 4% polyformaldehyde (PFA) as previously described [16] and then permeabilized and blocked in PBST (PBS and 1% Triton X‐100) containing 10% donkey serum at 4°C overnight. The specimens were incubated with the primary antibodies at 4°C overnight. The primary antibodies used in this study include: anti‐myosin VIIA (Myo7a)  (Proteus Biosciences, 1:1000 dilution), anti‐Tuj‐1 (Biolegend, 1:500 dilution), anti‐SRY (sex‐determining region Y)‐box 2 (Sox2) (Santa Cruz Biotechnology, 1:500 dilution), anti‐Smoothened (SMO) (Abcam, 1:100 dilution), anti‐Parvalbumin (Millipore, 1:500 dilution), anti‐4 Hydroxynonenal (4‐HNE, abcam, 1:500 dilution), anti‐FTH (Abcam, 1:500 dilution), anti‐lysosomal associated membrane protein 1 (LAMP1) (Santa cruz, 1:500 dilution), anti‐IREB2/IRP2+Aconitase1/ACO1 (Abcam, 1:500 dilution), anti‐ATF2 (phospho T71) (Abcam, 1:500 dilution). The specimens were then incubated with the corresponding fluorescent secondary antibody for 2 h at room temperature. DAPI (Sigma‐Aldrich, 1:800 dilution) was used to label nuclei. The specimens were detected with confocal fluorescence microscopy (Leica Microsystems, SP8).

### 2.6 RNAscope‐Based Small‐Molecule Fluorescent In Situ Hybridization (smFISH)

The RNAscope‐based smFISH was conducted to detect *Smo* mRNA expression in the postnatal mouse cochlea using RNAscope Multiplex Fluorescent Reagent Kit (ACD). Briefly, the frozen slices of the cochlea from P21 wild‐type mice or their neomycin‐treated littermates were prepared as previously described [17] and pretreated according to the ACD protocol. The specimens were then hybridized with the Mm‐*Smo*‐C1 probe (ACD) followed by Opal520 (Perkin Elmer) according to the manufacturer`s protocol and previous research [18, 19]. The specimens were further stained with anti‐Myo7a primary antibody and DAPI to label cochlear HCs and nuclei separately, and detected with confocal fluorescence microscopy.

### 2.7 Aminoglycosides and Noise Challenge and Trans‐Tympanic Drug Delivery

Five in vitro aminoglycoside‐induced HC death models were used in this study. Neomycin, gentamicin, tobramycin, amikacin, and kanamycin were applied to cochlear explants at the given dose mentioned above, and incubated for 6 h. After aminoglycoside withdrawal, cochlear explants were cultured in medium for 24 h before harvest.

Two in vivo aminoglycoside‐induced hearing loss models were applied in this study. Neomycin was administered through subcutaneous injection at a dose of 200 mg/kg weight daily for consecutive 8 days from P8 to establish a chronic aminoglycoside‐induced hearing loss model [20]. Hearing function was measured at 1‐month‐old, when the auditory function of mice upon damage had stabilized. Amikacin was administered by intraperitoneal injection for 15 consecutive days at a dose of 500 mg/kg [21]. Hearing function was measured at 7 days after the last injection of amikacin. 300 µm SANT‐1 dissolved in 5 µL poloxamer 407‐based thermosensitive hydrogel (PBS containing 30% Poloxamer 407 and 0.5% DMSO) was injected trans‐tympanically two hours before and 10 days after the first injection of amikacin to test its protective effect. The in vivo dose of SANT‐1 was determined according to previous research [22]. Mice were anesthetized with an intraperitoneal injection of 45 g/kg Zoletil (Virbac) and 1000 mg/kg dexmedetomidine hydrochloride (Dexdomitor) and then placed on a heating pad for body temperature maintenance. The drug/vehicle system was gently injected through the tympanic membrane with a thin glass microneedle under the surgical stereomicroscope as previously described [23].

Mice were exposed to 120 dB white noise for consecutive 2 h to establish a noise‐induced hearing loss model. HC loss from noise damage could continue for up to 10 days. Thus, we measured acute threshold shifts at 3 and 7 dpi, and measured permanent threshold shifts at 14 dpi.

Five‐week‐old mice were used to establish the amikacin and noise insult model. All mice used for in vivo experiments were randomly numbered to guarantee a uniform distribution of both sexes across all groups.

### 2.8 Auditory Brainstem Responses (ABR) Measurement

Hearing function was measured by ABR, which were electrical signals evoked from the brainstem stimulated by sound. The sound pressure of the stimulus at a given frequency was reduced from 90 to 20 dB in 5 dB steps. The ABR waveforms were documented using the TDT's RZ6 workstation and Bio‐Sig RP software (Tucker Davis Technology) with subdermal electrodes on the mastoid process, the skull surface, and the back of the tested mice. When the ABR waveform was just above the noise floor, the intensity of the stimulus (dB SPL) was recorded as the hearing threshold at the given frequency. The threshold above 90 dB was defined as 95 dB in this study. Hearing thresholds of 8, 16, 24, and 32 kHz were recorded in this study. ABR wave 1 amplitude was defined as the difference between the amplitude of the positive peak and the following negative trough of the first ABR wave.

### 2.9 siRNA‐Mediated Gene Knockdown

Genetic knockdown of *Fpn* (geneID:53945), *Cp* (geneID:12870), and *Atf2* (geneID:11909) was achieved through siRNA. The culture medium was replaced with Opti‐MEM medium (Thermo Fisher Scientific) one hour before transfection. Cochlear explants were transfected with approximately 100 nm target siRNA or scrambled siRNA diluted in Opti‐MEM using Lipofectamine3000 (Thermo Fisher Scientific). After 12‐hour transfection, the transfection medium was replaced with DMEM/F12 medium containing N2, B27, and ampicillin. The cochlear explants were collected for interfering efficacy validation using qPCR in 48 h after transfection, and challenged with neomycin in 72 h after transfection. The siRNA information was as follows:

si*Fpn*_1, sense, GCCCAGCUUUCCUGUUUAATT, antisense, UUAAACAGGAAAGCUGGGCTT. si*Fpn*_2, sense, GCCUGGCUUUCCUCUAUAUTT, antisense, AUAUAGAGGAAAGCCAGGCTT. si*Cp*_1, sense, CUGGCUGAAUGAAAUAAAUTT, antisense, AUUUAUUUCAUUCAGCCAGTT. si*Cp*_2, sense, GCCACCAAUUCAUGCAAAUTT, antisense, AUUUGCAUGAAUUGGUGGCTT. si*Atf2*_1, sense, GCAGAAGCUGUAGCCACUUTT, antisense, AAGUGGCUACAGCUUCUGCTT. si*Atf2*_2, sense, CUUCCGAAGAUGACAUUAATT, antisense, UUAAUGUCAUCUUCGGAAGTT. si*Atf2*_3, sense, CACCUCCACUACAGAAACUTT, antisense, AGUUUCUGUAGUGGAGGUGTT.

### 2.10 Western Blot

Protein extracts were obtained from cochlear explants (*n* = 8 cochlea for each group) or cochlear epithelia (*n* = 6 cochlea for each group) dissected from adult mice using RIPA lysis buffer (Beyotime) containing 1 mm PMSF (Beyotime), 1x protease and phosphatase inhibitor cocktail (Thermo Fisher).  Proteins were separated on SDS‐PAGE gels and transferred to PVDF membranes. After being blocked with QuickBlock blocking buffer (Beyotime), the membrane was incubated with the following primary antibodies overnight at 4°C: anti‐4‐Hydroxynonenal (4‐HNE, abcam, 1:2000 dilution), anti‐IREB2/IRP2+Aconitase1/ACO1 (Abcam, 1:2000 dilution), anti‐ATF2 (phospho T71) (Abcam, 1:2000 dilution), anti‐GAPDH (Beyotime, AF0006, 1:1000 dilution), anti‐FPN (Novus Biologicals, 1:2000 dilution). After washing with TBS‐T (1× tris‐buffered saline containing 1% Tween‐20), the membrane was incubated with the corresponding HRP‐labeled Goat Anti‐Mouse IgG(H+L) (Thermo Fisher Scientific, 1:2000 dilution) or/and HRP‐labeled Goat Anti‐Rabbit IgG(H+L) (Thermo Fisher Scientific, 1:2000 dilution) antibody for 2 h at room temperature. The protein bands were imaged using the Bio‐Rad imaging system. The data were obtained from at least three independent experiments.

### 2.11 HC Sorting and Bulk RNA Sequence

Cochlear explants extracted from P2 *Gfi1* ^Cre^/*Smo* ^2Amc/2Amc^/*ROSA26* *^CAG‐tdTomato^* and *Gfi1* ^Cre^/ *ROSA26* *^CAG‐tdTomato^* mice were obtained and cultured as forementioned. After incubated with neomycin for 4 h, cochlear explants were collected in cold PBS. The tissue was immersed in 1 mL 0.25% Trypsin‐EDTA solution at 37°C for cell digestion. During digestion, the tissues were gently flicked every 5 min to resuspend them. After digestion, 1 mL of culture medium was added to stop digestion, and the post‐digested tissues were gently triturated to single cells. The dissociation state of cells was observed under a microscope. Last, the obtained single‐cell suspensions were filtered through a 40 µm cell strainer (Millipore). *Gfi1*‐tdTomato+ cochlear hair cells were sorted with flow cytometry [24] and used for RNA extraction, library construction, and transcriptome sequencing.

Total RNA of the sorted cells was extracted using Trizol. The concentration of RNA was assessed using Qubit 4.0 (Thermo Scientific). mRNA was purified and used for library construction according to the manufacturer's protocol using the Stranded mRNA‐seq kit (Vazyme). The constructed libraries were sequenced using the Illumina Nova6000 to obtain sequencing data. Differential analysis was performed using the DESeq (2012) R package, where genes with a *p*‐value < 0.05 and a fold change > 1.5 in expression were considered statistically differentially expressed genes. These genes were further used for enrichment analysis.

### 2.12 Lipid Peroxides Detection Assays

Lipid peroxides were detected with three probes, Liperfluo, C11 BODIPY^581/591^, and MitoPeDPP, and 4‐HNE immunostaining and western blot. The latter two experiments were conducted as mentioned above. Liperfluo (Dojindo) was designed for exclusive detection of lipid peroxides rather than other reactive oxygen species (ROS). Once oxidized by lipid peroxides, Liperfluo emits intense fluorescence with the maximal emission wavelength at 535 nm. C11 BODIPY^581/591^ (Invitrogen) was a fluorescent radio‐probe for indexing membrane lipid peroxidation, the fluorescence of which shifted from red (*E*_m _= 595 nm) to green (*E*_m _= 520 nm) once oxidized by membrane lipid peroxides. The cochlear explants were cultured on the glass‐bottom dish and incubated with 5 µm Liperfluo or C11 BODIPY^581/591^ for 30 min at 37°C. After washing, the specimens were observed and imaged using confocal fluorescence microscopy with a live cell imaging system (Leica Microsystems). MitoPeDPP probe (Dojindo) was used to monitor Mitocondrial lipid peroxides according to the manufacturer`s protocol and procedures mentioned above.

### 2.13 Iron Detection Assays

Intracellular Fe^2^^+^ levels were monitored using the FerroOrange probe (Dojindo), which had a maximal emission wavelength of 580 nm, following the manufacturer's protocol. In HEI‐OC1 cells, intracellular Fe^2^^+^ levels were measured 4 h after challenge with neomycin, SAG, or FAC; whereas in HCs, levels were assessed 4 h after treatment with neomycin, SAG, or FAC, or 72 h after noise exposure. Due to the fixation‐sensitive nature of the FerroOrange probe, co‐staining with HC‐specific antibodies was not feasible. The HC region was identified based on the characteristic anatomical arrangement of HCs, which was visualized through nuclear staining with Hoechst [23, 25].

To detect intracellular Fe^2+^ content in the cochlear HCs after acoustic trauma, the frozen slices of the cochlea from noise‐insult adult mice and control ones were incubated with FerroOrange working solution according to the manufacturer`s protocol.

### 2.14 Cut&Tag

Approximately 1 × 10^6^ HEI‐OC1 cells under neomycin insult for 18 h were collected for the CUT&TAG assay. CUT&Tag was conducted using the Hyperactive In Situ ChIP Library Prep Kit for Illumina (Vazyme Biotech, China) according to the manufacturer's protocol [26]. Briefly, HEI‐OC1 cells were resuspended and bound to concanavalin A‐coated magnetic beads. Bound cells were permeabilized using digitonin, and then incubated with anti‐ATF2 (phospho T71) (Abcam), secondary antibody, and pA‐Tn5 transposase. DNA bound to p‐ATF2 was cut into fragments by pA‐Tn5 transposase. p‐ATF2‐bound DNA fragments were ligated with P5 and P7 adaptors and further amplified using PCR with the P5 and P7 primers. PCR products were evaluated using the Agilent 2100 Bioanalyzer (Agilent Technologies) and sequenced on the Illumina NovaSeq6000 platform.

The raw sequence data were trimmed by fatsp software to obtain the clean reads, which were further aligned to the mouse genome (GRCm39/mm39) using the Bowtie2 software and subsequently analyzed by the SEACR software to detect putative binding sites. Peaks were annotated using ChipSeeker software. The significantly different peaks were identified with the threshold of *p*‐value < 0.05 and |*M*‐value| > 1 using Manorm software for GO enrichment analysis.

### 2.15 Plasmids Transfection and Dual‐Luciferase Assay

To overexpress ATF2, we cloned the transcript of the mouse ATF2 gene (NM_001025093.2) in the pcDNA3.1 vector to construct ATF2‐OE plasmids. We mutated the phosphorylation sites threonine51/53 to aspartic acid to mimic phosphorylation activation (p‐ATF2‐OE plasmids). pcDNA3.1 vectors were used as controls. The promoter region of the mouse *Irp1* gene (ch4, NC_000070.7, 40141429∼40144429) was cloned in the pGL4 vector to construct *Irp1*‐promoter plasmids. The predicted ATF2 binding site (ch4, 40143646∼40143658) was deleted to construct mutated *Irp1*‐promoter plasmids (△*Irp1*‐promoter). The Renilla luciferase reporter (pGL4‐RL) plasmids were used as the internal reference. All plasmids were constructed by GenePharm, China.

ATF2‐OE or pATF2‐OE plasmids were co‐transfected with Irp1‐promoter and pGL4‐RL plasmids into HEI‐OC1 cells using Lipofectamine3000 to examine the role of p‐ATF2/ATF2 on Irp1 expression. Cells were harvested to detect the luciferase activity in 48 h after transfection using the Duo‐Lite Luciferase Assay kit (Vazyme) according to the manufacturer's protocol.

### 2.16 Statistics

Data were represented as mean ± standard deviation (SD). Statistical analysis was conducted with GraphPad Prism 9 software, utilizing data gathered from a minimum of three independent experiments. For comparing two groups, a two‐tailed Student's *t*‐test was employed. For comparing multiple groups, one‐way analysis of variance (ANOVA) with Bonferroni's multiple comparison or two‐way ANOVA with a post‐hoc Student Newman–Keuls test was applied. ns means no significant difference, */^#^ *p *< 0.05, **/^##^ *p* < 0.01, ***/^###^ *p* < 0.001, ****/^####^ *p *< 0.0001.

## 3 Results

### 3.1 Disruption of Iron Homeostasis Contributed to HC Damage During SNHL

**FIGURE 2:**
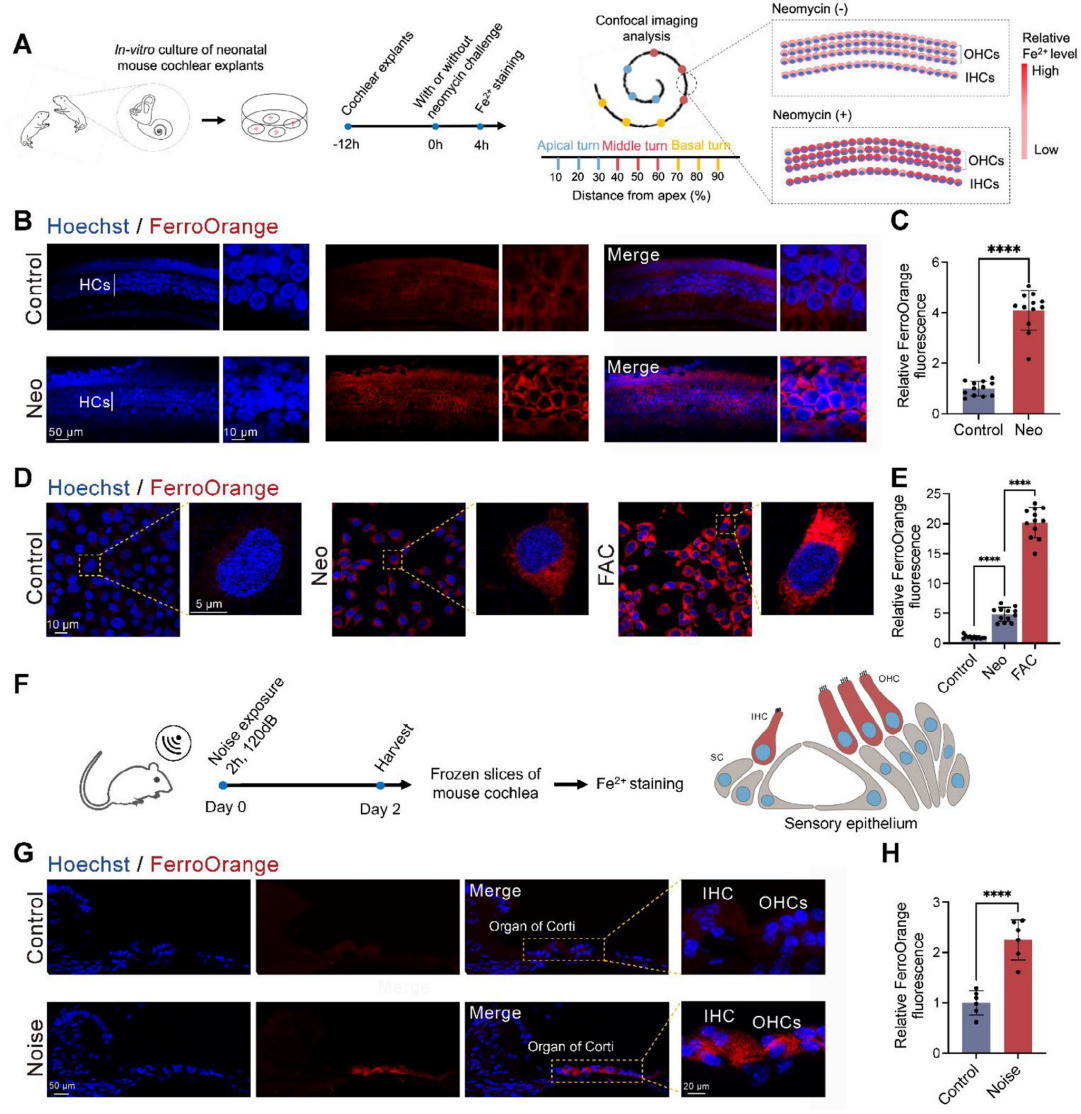
Excessive iron accumulation occurred in damaged HCs during SNHL. (A–C) Experimental design (A), representative images (B), and quantitative analysis (C) of FerroOrange staining (red) in cochlear HCs after neomycin injury. (D,E) Representative images (D) and quantitative analysis (E) of FerroOrange staining (red) in HEI‐OC1 cells after neomycin injury or FAC (2mM) treatment. (F–H) Experimental design (F), representative images (G), and quantitative analysis (H) of FerroOrange staining (red) in the cochlea after noise exposure. Statistical analysis: two‐tailed Student's *t‐*test was employed for (C) and (H); two‐way ANOVA with a post‐hoc Student Newman–Keuls test was employed for (E). ns means no significant difference, * *p *< 0.05, ** *p* < 0.01, *** *p* < 0.001, **** *p* < 0.0001.

Cochlear HCs are the most vital but delicate mechano‐electrical sensors necessary for auditory perception [27], and they are vulnerable to noise [28, 29] and to ototoxic drugs like aminoglycoside antibiotics [30]. Neomycin, a commonly used aminoglycoside antibiotic, effectively induces cochlear HC damage, serving as a robust approach for inducing the SNHL model. To demonstrate the disruption of iron homeostasis during HC damage, we first measured the relative intracellular content of ferrous ions after neomycin exposure via the specific Fe^2+^ indicator FerroOrange [31] using cochlear explants and HEI‐OC1 cells, a cochlear cell line widely used for studying ototoxic mechanisms and screening potential ototoxic or otoprotective drugs [32] (Figure 2A). The intracellular ferrous ions significantly increased in both cochlear HCs (Figure 2B,C) and HEI‐OC1 cells following neomycin damage (Figure 2D,E). Given the mechanistic similarities between noise and aminoglycosides‐induced hearing loss [33], we further investigated the iron content in the mouse cochlea after noise injury (Figure 2F), and found notable noise‐induced accumulation of Fe^2+^ in the sensory epithelium (Figure 2G,H).

**FIGURE 3:**
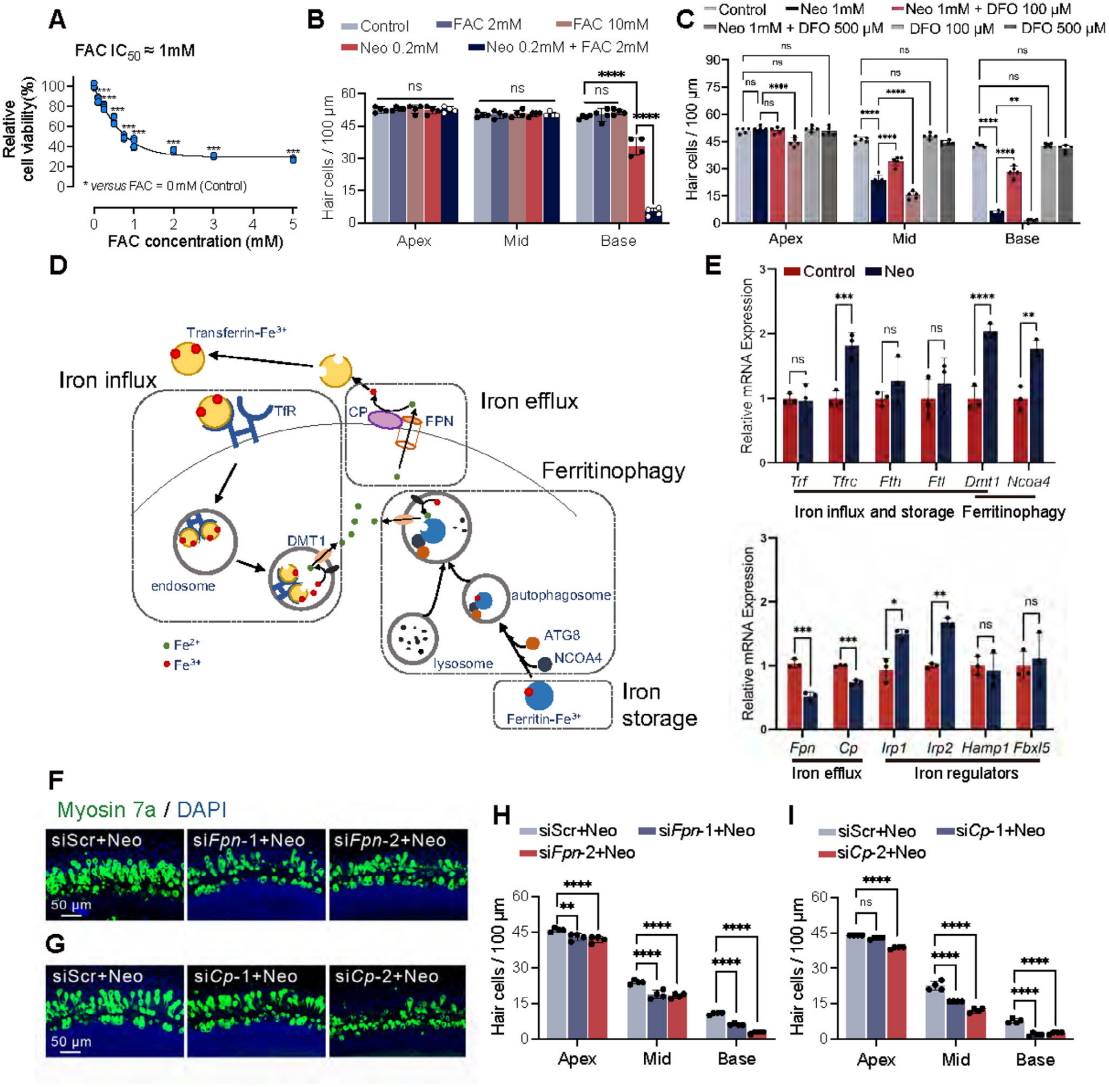
Disruption of iron homeostasis contributed to HC damage. (A) Analysis of HEI‐OC1 cell viability after incubating with a gradient dose of FAC for 24 h by CCK8 assay. (B) Quantitative analysis of HCs from the apical, middle, and basal turns of the cochlea after incubating with low‐dose neomycin (0.2 mm) and/or FAC in vitro. (C) Quantitative analysis of cochlear HCs after neomycin exposure with or without different doses of DFO in vitro. (D) Diagram showing intracellular iron metabolism regulation. (E) qPCR analysis of *Trf, Tfrc, Fth, Ftl, Dmt1, Ncoa4, Fpn, Cp, Irp1, Irp2, Hamp1*, and *Fbxl5* after neomycin damage. (F–I) Representative images (F,G) and quantitative analysis (H,I) of cochlear HCs after neomycin exposure with different siRNAs. Statistical analysis: one‐way analysis of variance (ANOVA) with Bonferroni's multiple comparison was employed for (A); two‐tailed Student's *t*‐test was employed for (E); two‐way ANOVA with a post‐hoc Student Newman–Keuls test was employed for (B), (C), (H), and (I). ns means no significant difference, * *p *< 0.05, ** *p* < 0.01, *** *p* < 0.001, **** *p* < 0.0001.

To directly assess iron toxicity, we established an in vitro iron overload model by adding ferric ammonium citrate (FAC) to HEI‐OC1 cultures. FAC induced elevated Fe^2+^ content and dose‐response cell death in HEI‐OC1 cells (Figure 3A; Figure S1A,B), and clearly aggravated neomycin‐induced iron accumulation and cochlear HCs loss (Figure 3B; Figure S1A–D). Together, these results indicated that excessive iron overload contributed to neomycin‐induced HC loss. Co‐treatment with the iron chelator deferoxamine (DFO, 100 µm) rescued HCs from FAC‐induced damage. However, DFO at higher doses induced ototoxicity, highlighting the narrow therapeutic window of systemic chelation (Figure 3C).

Iron homeostasis is maintained mainly by three physiological processes involving multiple iron metabolism‐related proteins: (1) iron influx, mediated by transferrin (Tf)‐transferrin receptor (TfR) endocytosis and divalent metal transporter 1 (DMT1); (2) cellular iron availability and storage, which is controlled by ferritin turnover and nuclear receptor coactivator‐4 (NCOA4)‐mediated ferritinophagy; and (3) iron efflux through ferroportin (FPN), the only known iron export pump (Figure 3D) [12, 34]. Ceruloplasmin (CP), a ferroxidase, is found to be functionally coupled with FPN on the cell membrane to facilitate iron efflux [35]. To further elucidate the pathological mechanism of iron overload in cochlear HC injury, we assessed the expression levels of key genes involved in iron homeostasis regulatory pathways. Consistent with prior reports, our results revealed upregulation of *Tfrc* (encoding the transferrin receptor) and *Ncoa4* (a ferritinophagy regulator) expression in cochlear explants exposed to 1 mm neomycin for 4 h (Figure 3E), indicating the activation of iron endocytosis and ferritinophagy pathways post‐injury. The flavonoid luteolin has been shown to inhibit ferroptosis by downregulating *Tfrc* expression, thereby reducing intracellular iron levels and protecting HCs from cisplatin‐induced damage [6]. Our previous study indicates that Nuciferine protects cochlear HCs from cisplatin‐induced ferroptosis by inhibiting *Ncoa4*‐mediated ferritinophagy, thereby reducing intracellular iron accumulation and oxidative stress [15]. Notably, we observed significant downregulation of *Fpn* (also known as *Slc40a1*) and *Cp* expression in the damaged cochleae (Figure 3E), suggesting suppression of iron efflux, which has not been previously described during the pathological progression of HC damage.

To further investigate the role of FPN and CP in cochlear HC injury, we knocked down *Fpn* and *Cp* expression using siRNA. Silencing *Fpn* or *Cp* expression markedly exacerbated intracellular iron overload during injury and reduced HC resistance to neomycin toxicity (Figure 3F–I; Figure S2A,B), while transfection with either si*Fpn* or si*Cp* alone, in the absence of neomycin, did not affect HC survival (Figure S2C,D). These data demonstrate that FPN and CP‐mediated iron efflux is critical for maintaining cochlear HC iron homeostasis.

Taken together, cochlear HCs exhibit iron overload during injury, which serves as a critical pathological mechanism. Our study identified three key contributors to iron accumulation: 1) enhancement of TFR‐mediated iron endocytosis, 2) enhancement of NCOA4‐driven ferritinophagy, and 3) impairment of FPN/CP‐mediated iron efflux. These findings collectively elucidate how dysregulation of iron influx, recycling, and efflux systems synergistically drives pathological iron overload in damaged HCs.

### 3.2 SMO Inhibition Exhibited Otoprotective and Iron Homeostasis‐Regulating Effects

**FIGURE 4:**
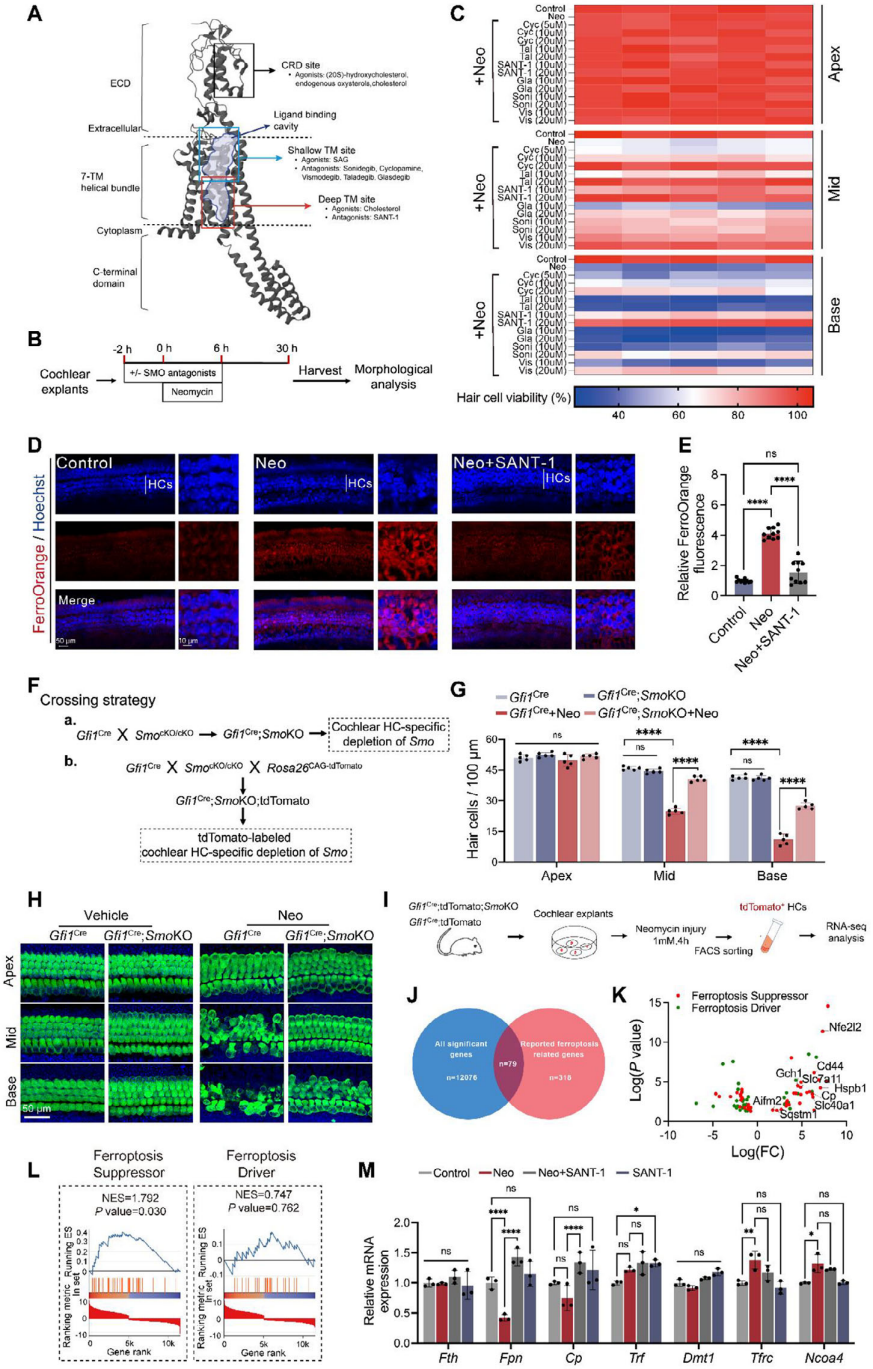
SMO inhibition exhibited otoprotective and iron homeostasis regulating effect. (A) SMO structure (mouse, Uniprot ID: P56726.) and binding sites of small molecules. ECD: Extracellular domain. 7‐TM: Seven‐transmembrane domain. CRD: Cysteine‐rich domain. (B) Experimental design for evaluating the otoprotective effects of SMO inhibitors against neomycin‐induced HC damage. (C) The otoprotective profiles of six SMO inhibitors evaluated with dose‐response experiments in cochlear explants. (D,E) Representative images (D) and quantitative analysis (E) of FerroOrange staining (red) in cochlear HCs after neomycin exposure with or without SANT‐1. (F) Mouse lines and breeding strategy. “*Smo* ^cko/cko^” refers to the *Smo* ^2Amc/2Amc^ mice. (G‐H) Representative images (H) and quantitative analysis (G) of Myo7a^+^ cochlear HCs harvested from neonatal *Gfi1* ^Cre^ or *Gfi1* ^Cre^/*Smo*KO mice with or without neomycin damage in vitro. (I) Experimental design for cochlear HC sorting and bulk mRNA sequencing. (J) The Venn plot showing the intersection between differentially expressed genes from the RNA‐seq dataset and the Ferdb database. Out of the 318 ferroptosis‐related genes, 79 genes showed differential changes in *Smo*‐deficient HCs. (K) Volcano plot displaying differentially expressed genes of the ferroptosis pathway. (L) GSEA analysis of the ferroptosis pathway. Ferroptosis suppressor genes were significantly enriched in the *Smo*KO group (NES = 1.792, *p* = 0.030). (M) qPCR validation of differentially expressed genes. Statistical analysis: two‐way ANOVA with a post‐hoc Student Newman–Keuls test was employed for (E), (G) and (M). ns means no significant difference, * *p *< 0.05, ** *p* < 0.01, *** *p* < 0.001, **** *p* < 0.0001. FC, fold change.

As previously highlighted, modulating iron homeostasis in cochlear HCs represents a promising therapeutic strategy for drug‐ and noise‐induced SNHL. However, effective iron homeostasis regulators remain scarce. Screening clinically approved drugs for repurposing is a validated strategy to accelerate therapeutic development, as it circumvents the lengthy de novo drug discovery processes. In our preliminary studies, we discovered that multiple FDA‐approved SMO inhibitors, including vismodegib (Vis), sonidegib (Soni), SANT‐1, Taladegib (Tal), and glasdegib (Gla), effectively protected HEI‐OC1 cells from FAC‐induced injury (Figure 4A; Figure S3A–C). Dose‐response experiments were then conducted for six SMO inhibitors to screen for their otoprotective potency (Figure 4B). As depicted in Figure 4C, all six SMO inhibitors alleviated neomycin‐induced HC loss. Of these six compounds, SANT‐1, exhibited the best profile based on its protective activity in cochlear explants, as evidenced by the greatest reduction in HC loss in the SANT‐1 co‐treatment group. Besides, in the presence of SANT‐1, excessive accumulation of iron in both HEI‐OC1 cells and cochlear HCs after neomycin or FAC treatment was restored, as evidenced by the repression of FerroOrange fluorescence (Figure 4D,E; Figure S3D–G). These findings suggest that SMO inhibitors may serve as otoprotection agents by modulating iron homeostasis.

SMO, a 7‐pass transmembrane protein, has been well studied as the transducer of the Hedgehog (Hh) signaling. Previous work has demonstrated that SMO‐mediated Hh signaling plays a significant role in the embryogenesis of the inner ear [36, 37]. However, the role of SMO in the cochlear HC survival and iron metabolism regulation has never been reported. We then tested whether SMO deficiency could protect mice from aminoglycoside‐induced HC death. We generated *Gfi1* ^Cre^/*Smo* ^2Amc/2Amc^ (*Gfi1* ^Cre^/*Smo*KO) mice [38], in which Cre recombinase removes the floxed sequence and creates a null *Smo* allele in *Gfi1* ^+^ HCs, and *Gfi1* ^Cre^ mice lacking the *Smo* ^2Amc^ allele were used as controls (Figure 4F). We cultured cochlear explants from *Gfi1* ^Cre^/*Smo*KO mice at postnatal day 3 (P3) and conducted neomycin challenge experiments. The results suggested that *Smo* deficiency greatly suppressed neomycin‐induced HC loss (Figure 4G,H).

Next, we delved into the downstream mechanism through which SMO inhibition exerts its effect on otoprotection and iron metabolism regulation. To identify the differential gene transcriptional programs altered by genetic deletion of *Smo* in HCs, we generated *Gfi1* ^Cre^ */Smo* ^2Amc/2Amc^ */ROSA26^CAG‐tdTomato^* mice to label all cochlear HCs with red fluorescence and then sorted out cochlear HCs via flow cytometry for RNA sequencing (RNA‐seq) after neomycin treatment (Figure 4I). *Gfi1* ^Cre^ */ROSA26^CAG‐tdTomato^* mice without the *Smo* ^2Amc^ allele were used as controls. We analyzed the differentially expressed genes using the Ferrdb database [39], a comprehensive resource for ferroptosis‐related genes, to reveal the potential relationship between *Smo* depletion and iron dysregulation‐related cell death in cochlear HCs. Among 319 reported ferroptosis‐regulating genes, 79 were identified in our differentially expressed gene sets (Figure 4J). Gene set enrichment analysis (GSEA) based on the Ferrdb database indicated the upregulation of the ferroptosis suppression pathway (Figure 4K,L; Figure S4A). Secondary validation via qPCR indicated that SMO inhibition altered the expression of these genes, especially those involved in iron homeostasis, including upregulation of *Cp, Fpn* (Figure 4M; Figure S4B). We also observed a downregulation trend in *Tfrc* expression. These results suggested that SMO inhibition exerts its iron homeostasis regulation effect through targeting iron influx and efflux.

Note that *Ncoa4* expression was upregulated after neomycin insult. Co‐localization between FTH and LAMP‐1 (a lysosome marker) in HEI‐OC1 cells also significantly increased after neomycin damage, as evidenced by immunofluorescence co‐localization assays. However, the *Ncoa4* expression level and the co‐localization between FTH and LAMP‐1 did not differ with or without SANT‐1 treatment (Figure S5A,B), suggesting SMO inhibition did not target ferritinophagy to regulate iron homeostasis.

### 3.3 SMO Activation Contributed to Iron Overload and HC Death During SNHL

As mentioned, SMO inhibition effectively attenuated cochlear HC damage by reducing iron overload and subsequent cell death. The current findings prompt critical questions: Does neomycin exposure induce SMO activation through specific signaling mechanisms? Could SMO signaling represent a key regulatory pathway that exacerbates iron accumulation while amplifying cochlear HC death during SNHL? Resolving these mechanistic uncertainties will be essential for understanding the pathogenesis of SNHL.

**FIGURE 5:**
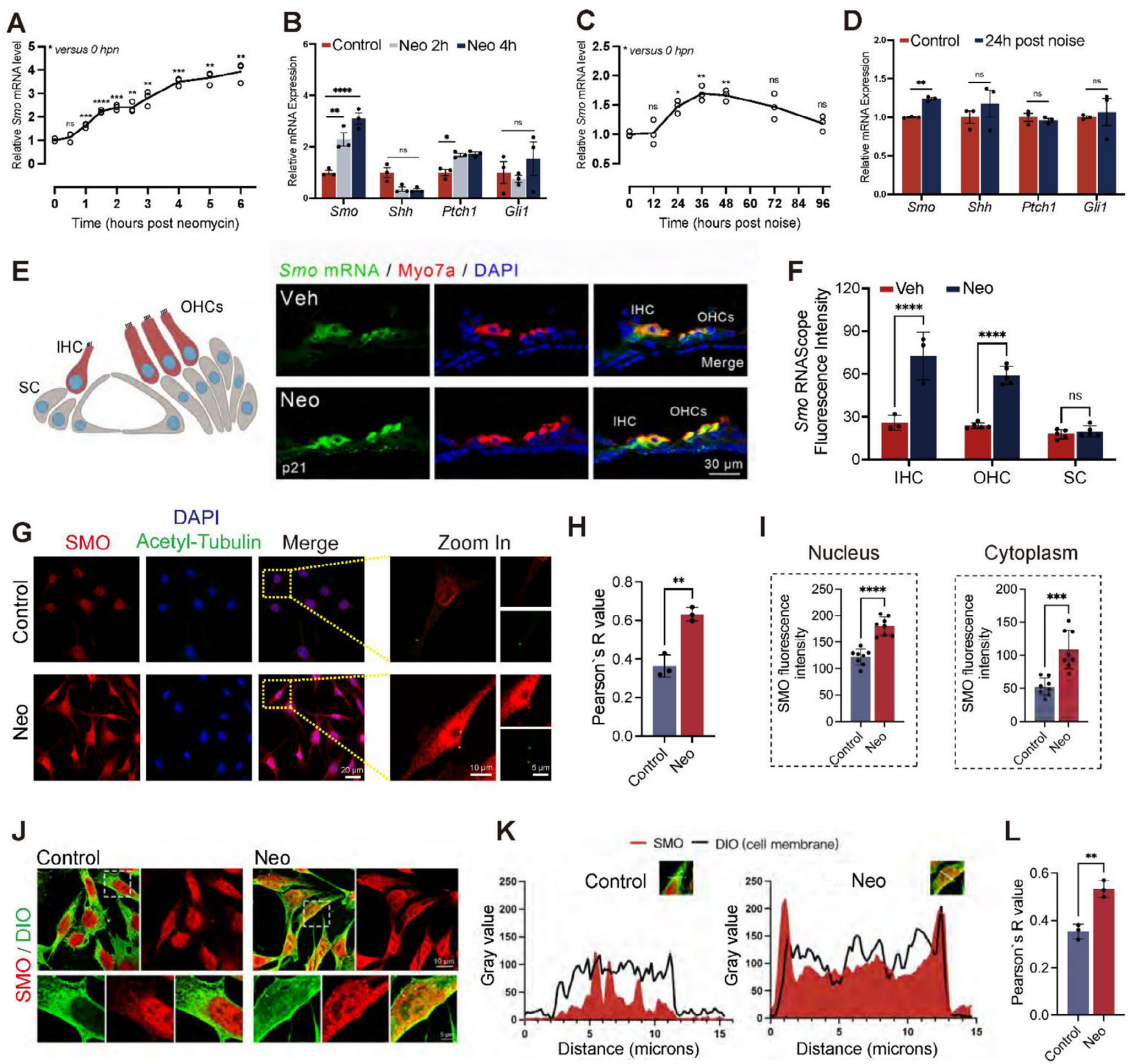
Insult‐induced SMO activation in cochlear HCs. (A) The *Smo* expression pattern in cultured cochlear explants after neomycin injury. hpn, hours post‐neomycin. (C) The *Smo* expression pattern in cochlear epithelia after noise exposure. hpn, hours post noise. (B,D) Expression of key genes in Hedgehog signaling in cochleae after neomycin (B) or noise (D) insult. (E) RNAScope images of *Smo* (green probe) in the organ of Corti of P21 mice. See also Figure S6B for individual optical sections, demonstrating signal distribution across adjacent hair cells within the section. (F) Quantitative analysis of the *Smo* RNAScope fluorescence intensity. (G) Immunofluorescent labeling of SMO (red) and acetylated α‐tubulin (green, a marker for primary cilia) in HEI‐OC1 cells following neomycin injury. (H) Quantitative co‐localization analysis between the SMO immunofluorescence signal and acetyl‐tubulin signal in HEI‐OC1 cells. (I) Quantitative analysis of SMO fluorescence signal in the cytoplasm and nucleus of HEI‐OC1 cells. (J) Immunofluorescent labeling of SMO (red) and cell membranes (DIO, green) in HEI‐OC1 cells. The lower row of images is enlarged views of the areas outlined by white dashes. (K) Quantitative analysis of SMO and DIO fluorescence intensity along the white dashed line as shown in the top‐right corner image. (L) Quantitative co‐localization analysis between the SMO immunofluorescence signal and the membrane marker signal (DIO) in HEI‐OC1 cells. Statistical analysis: one‐way analysis of variance (ANOVA) with Bonferroni's multiple comparison was employed for (A–C); two‐tailed Student's *t‐*test was employed for (D), (F), (H), (I), and (L). ns means no significant difference, * *p *< 0.05, ** *p* < 0.01, *** *p* < 0.001, **** *p* < 0.0001.

To address this question, we first examined the expression pattern of *Smo*/SMO in cochlear sensory epithelium from postnatal mice using RNAscope‐based small‐molecule fluorescent in situ hybridization (smFISH) and immunofluorescence. The protein and mRNA of *Smo* were observed in HCs, including both outer HCs (OHCs) and inner HCs (IHCs), as well as in supporting cells (SCs) (Figure 5E; Figure S6A,B). Interestingly, qPCR results demonstrated a significant upregulation of *Smo* expression observed in mouse cochleae upon neomycin injury (Figure 5A,B). smFISH results showed that *Smo* was upregulated in cochlear HCs after neomycin injury (Figure 5E,F). Consistent with the mRNA data, quantitative immunofluorescence analysis revealed a significant increase in SMO protein levels in both HEI‐OC1 cells (Figure 5G,I) and in cochlear HCs (Figure S6C,D) following neomycin injury. The qPCR results also confirmed that *Smo* expression was significantly enhanced in a time‐dependent manner after acoustic trauma (Figure 5C,D). The next question was whether injury led to SMO activation. Interestingly, *Shh* and *Gli1*, which encode the key endogenous ligands and downstream effectors of the Hh signaling cascade and are the widely accepted markers for canonical Hh signaling activation [40, 41, 42], remained unchanged after either neomycin or noise injury (Figure 5B,D). Another hallmark of SMO activation is its re‐localization and accumulation on the primary cilia and cell membrane [43]. Using HEI‐OC1 cells, we co‐stained for SMO and acetylated α‐tubulin (a marker for primary cilia) following neomycin injury. As shown in Figure 5G,H, we observed a clear increase in the co‐localization of SMO with primary cilia​ after neomycin treatment compared to controls, suggesting that SMO was indeed activated by ototoxic injury. Interestingly, we also observed increased membrane association of SMO after neomycin injury in OC1 cells, as evidenced by enhanced co‐localization between SMO staining and DIO, a cellular plasma membrane probe (Figure 5J,K). Quantitative co‐localization analysis revealed a significant increase in the Pearson's correlation coefficient between SMO and the plasma membrane marker upon neomycin treatment compared to controls, objectively confirming SMO translocation to the membrane (Figure 5L). Together, these results indicated that injury induced SMO upregulation and activation in cochlear HCs, which led to an unresolved question about the potential role of the SMO signaling in the pathogenesis of ototoxicity.

**FIGURE 6:**
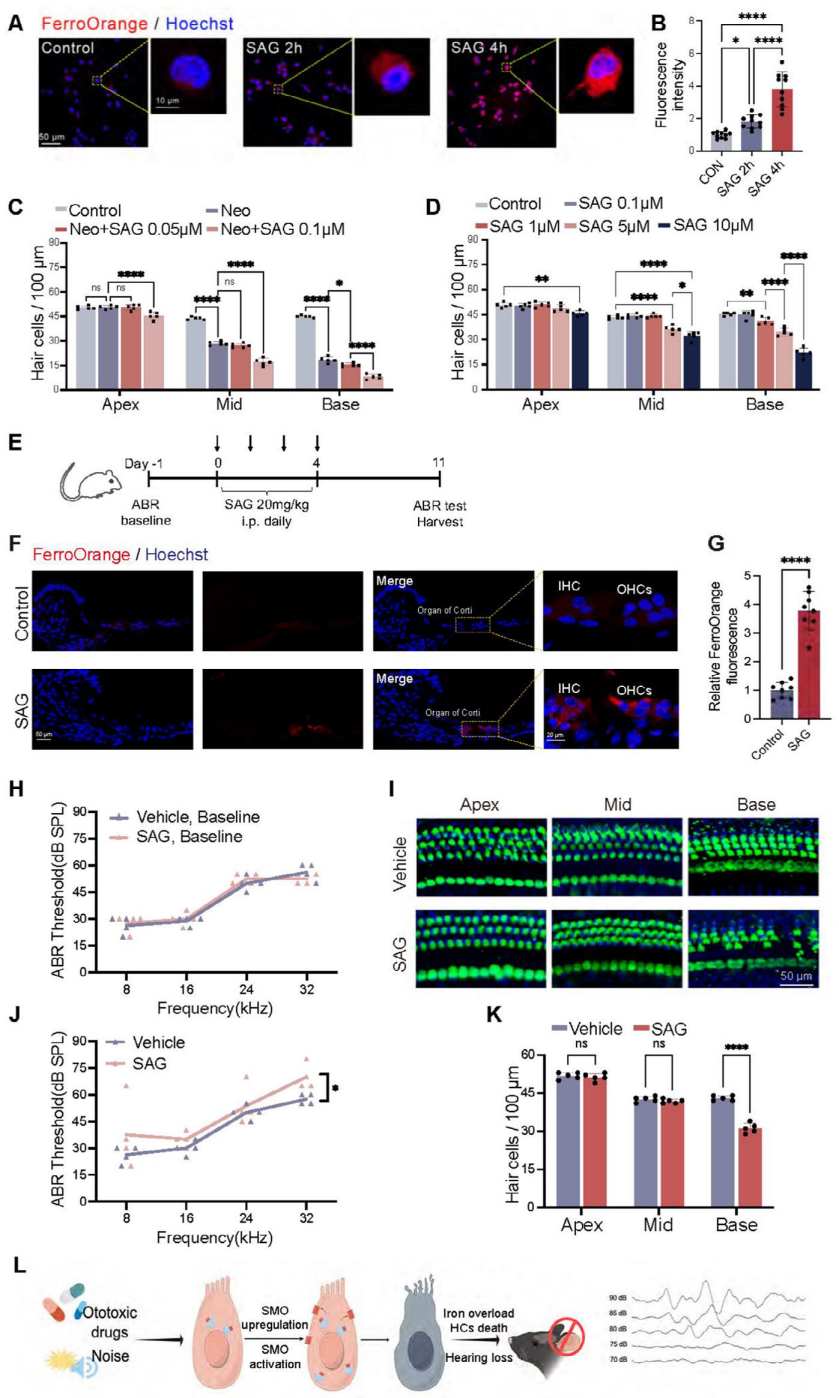
SMO activation contributed to iron overload and HC death during SNHL. (A,B) Representative images (A) and quantitative analysis (B) of FerroOrange staining (red) in HEI‐OC1 cells after SAG (5 µm) treatment. (C) Quantitative analysis of cochlear HCs after neomycin damage with or without SAG. (D) Quantitative analysis of HCs in the cochlear explants treating with a series of doses of SAG. (E) Experimental design of the intraperitoneal injection of SAG in C57BL/6 wide‐type mice. (F,G) Representative images (F) and quantitative analysis (G) of FerroOrange staining (red) in the cochlea after SAG challenge. (H,J) ABR thresholds of C57BL/6 wide‐type mice before (H) and after (J) treatment with SAG or vehicle. *n* = 4 mice for each group. (I,K) Representative images and quantitative analysis of Myo7a^+^ cochlear HCs after SAG treatment in vivo. (L) Diagram showing SMO activation caused cochlear HC death and hearing loss in mice. SPL, sound pressure level. Statistical analysis: one‐way analysis of variance (ANOVA) with Bonferroni's multiple comparison was employed for (B); two‐tailed Student's *t*‐test was employed for (G), (H), (J), and (K). The statistical significance was analyzed for the ABR threshold of each independent frequency between the vehicle and SAG group in (H) and (J); two‐way ANOVA with a post‐hoc Student Newman–Keuls test was employed for (C) and (D). ns means no significant difference, * *p *< 0.05, ** *p* < 0.01, *** *p* < 0.001, **** *p* < 0.0001.

Next, we explored the potential impact of SMO activation on iron homeostasis and HC death in response to aminoglycoside exposure. SAG is a specific SMO agonist with effective dosages ranging from 0.1 nm to 100 µm [40, 44]. To investigate whether SMO activation directly contributes to iron dyshomeostasis, we treated HEI‐OC1 cells with the SMO agonist SAG for 2–4 h. FerroOrange fluorescence quantification revealed a time‐dependent iron accumulation in SAG‐treated cells (Figure 6A,B). Co‐treatment with low concentrations of SAG (0.05 and 0.1 µm) distinctly exacerbated neomycin‐induced HC loss (Figure 6C). Moreover, administration of SAG at higher doses (≥1 µm) alone induced HC loss in a dose‐dependent manner, also showing a basal to apical gradient of ototoxicity similar to neomycin (Figure 6D). We revalidated the ototoxicity of SAG in adult mice with peritoneal injection of SAG and assessed hearing function using the auditory brainstem response (ABR) test at 1 week after SAG administration (Figure 6E). SAG treatment significantly increased FerroOrange fluorescence in cochlear HCs (Figure 6F,G), indicating that SMO activation is sufficient to elevate intracellular ferrous iron levels. Notably, SAG caused significant elevation of hearing thresholds at 32 kHz (Figure 6H,J) and corresponding HC loss in the basal turn of the cochlea (Figure 6I,K). These findings establish a causal relationship between SMO activation and iron dysregulation, demonstrating that SMO activation exacerbates cochlear HC injury (Figure 6L).

### 3.4 SMO Inhibition Ameliorated the Accumulation of Lipid Peroxides and HC Ferroptosis During SNHL

**FIGURE 7:**
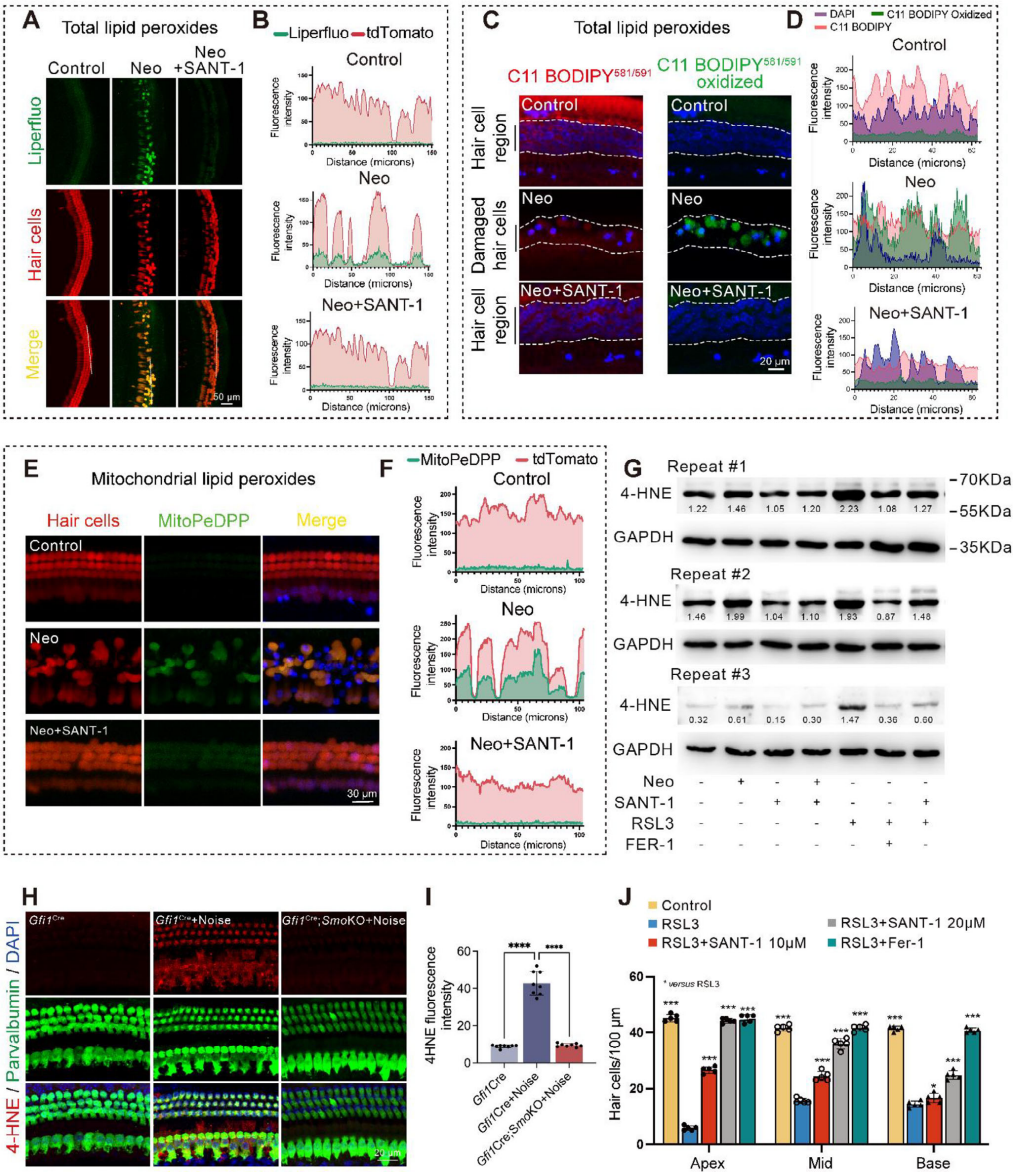
SMO inhibition ameliorated the accumulation of lipid peroxides and HC ferroptosis during SNHL. (A,B) Representative images and quantitative analysis of Liperfluo (green) staining in *Gfi1* ^Cre^ */ROSA26^CAG‐tdTomato^* cochlea explants (HCs were labeled with tdTomato (red)). (C,D) Detection of lipid peroxides by C11 BODIPY^581/591^ staining. (E,F) Detection of mitochondrial lipid peroxides by MitoPeDPP staining. (G) Western blot analysis of 4‐HNE for the indicated groups. The expression levels (relative to GAPDH) are indicated below the 4‐HNE protein bands. (H,I) Representative images (H) and quantitative analysis (I) of 4‐HNE staining (red) at 3 days after noise exposure. HCs were labeled with parvalbumin (green). (J) Quantitative analysis of cochlear HCs after RSL3 treatment with or without Fer‐1 and SANT‐1. Statistical analysis: two‐way ANOVA with a post‐hoc Student Newman–Keuls test was employed for (I) and (J). ns means no significant difference, * *p *< 0.05, ** *p* < 0.01, *** *p* < 0.001, **** *p* < 0.0001.

Excessive cellular iron load, especially free Fe^2+^, catalyzes lipid peroxides into more destructive lipid peroxyl radicals through the Fenton reaction and eventually contributes to ferroptosis, which is characterized by intracellular iron overload and deleterious accumulation of lipid peroxides. The increase of lipid peroxides like 4‐hydroxynonenal (4‐HNE) is widely recognized as a hallmark of ferroptosis [45, 46]. Our results suggested that SMO inhibition significantly reduced neomycin and noise induced total intracellular lipid peroxides overload in cochlear HCs, as evidenced by the lipid peroxide probe Liperfluo (Figure 7A,B; Figure S7A,B) and C11 BODIPY^581/591^ (Figure 7C,D; Figure S7C,D), and the detection of 4‐HNE content with immunofluorescence and western‐blot (Figure 7G–I). Moreover, we found that mitochondrial lipid peroxides were also downregulated by SANT‐1, as demonstrated by MitoPeDPP staining (Figure 7E,F; Figure S7E,F).

The more direct approach to investigate the relationship between SMO inhibition and ferroptosis is to examine the effect of SMO inhibition on HC damage induced by ferroptosis inducers. Interestingly, RSL3, a specific ferroptosis inducer, caused severe OHC death along the entire length of the cochlea but little IHC loss (Figure 7J; Figure S8). RSL3‐induced HC ferroptosis could be rescued by both SANT‐1 and Fer‐1 (Figure 7J; Figure S8). Taken together, these results suggested that ferroptosis contributed to both noise and neomycin‐induced HC death, which could be ameliorated by SMO inhibition.

### 3.5 SMO Inhibition Restored Iron Homeostasis to Alleviate Ferroptosis via PKC‐ATF2‐IRP1‐FPN/TFRC Axis

**FIGURE 8:**
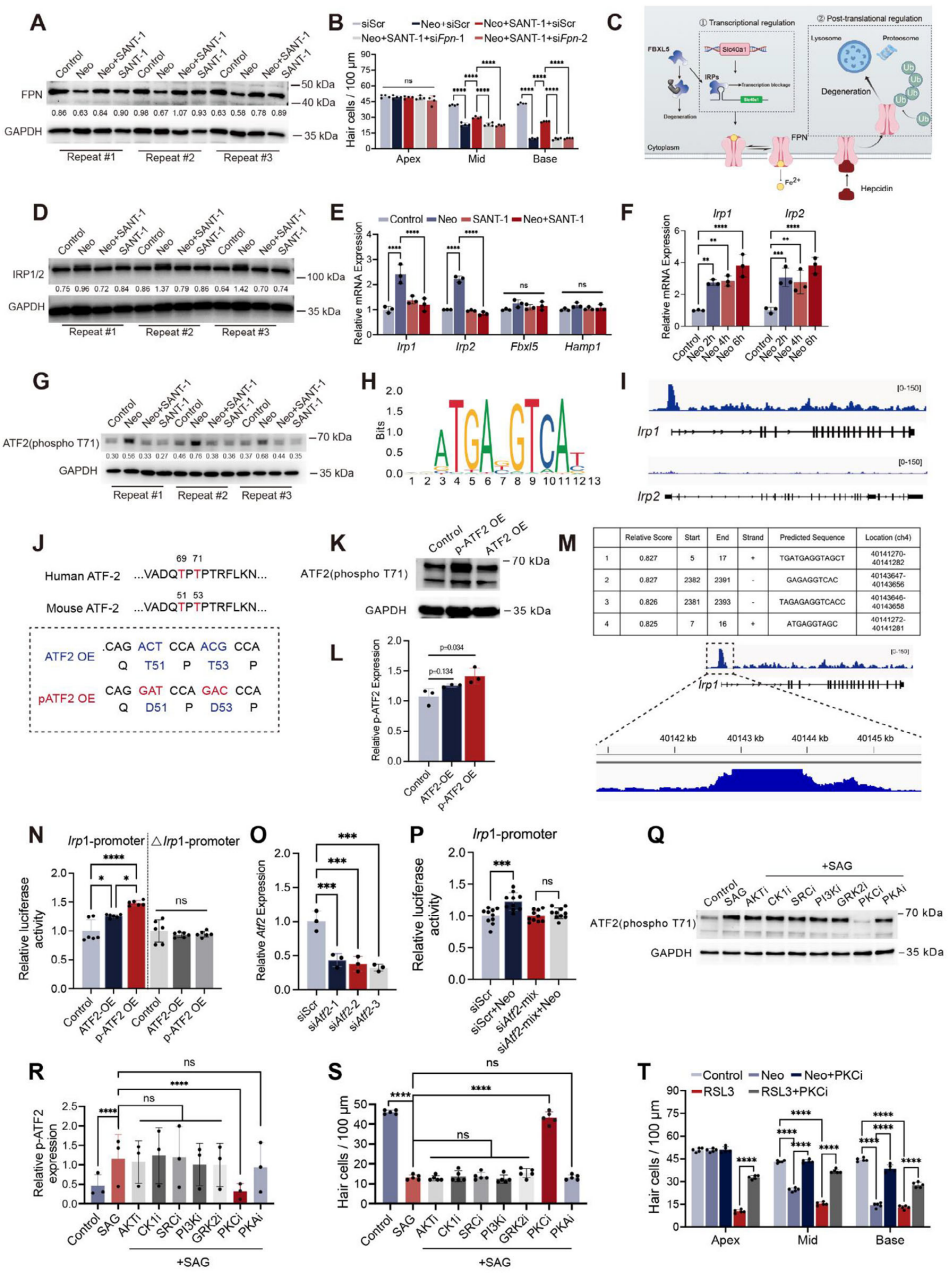
SMO inhibition restored iron homeostasis to alleviate ferroptosis via PKC‐ATF2‐IRP1‐FPN/TFRC axis. (A) Western blot analysis of FPN. (B) Quantitative analysis of cochlear HCs after neomycin and SANT‐1 treatment with different siRNAs. (C) Diagram showing the regulation of FPN expression at the transcriptional and post‐translational levels. (D) Western blot analysis of IRPs. (E) qPCR analysis of *Irp1*, *Irp2*, *Fbxl5*, and *Hamp1*. (F) qPCR analysis of *Irp1* and *Irp2* expression at various time points post‐neomycin damage. (G) Western blot analysis of ATF2(phosphor T71). (H) Predicted binding motif of p‐ATF2. (I) The binding tracks for p‐ATF2 at the genomic loci of *Irp1* and *Irp2* identified by calling peaks in the CUT&TAG‐seq data. (J) Top: Comparison of phosphorylation sites in human and mouse ATF2. Bottom: Schematic of overexpression of the phospho‐mimetic ATF2 mutant, with threonine 51 and 53 mutated to aspartic acid to mimic constitutive phosphorylation. (K,L) Validation of the overexpression effects of ATF2 and p‐ATF2 plasmids by Western blot. (M) The predicted p‐ATF2 binding sites within the promoter region of *Irp1* and their specific locations. (N) Binding of p‐ATF2 to the promoter of *Irp1* in HEI‐OC1 cells analyzed by dual luciferase assay. (O) qPCR validation of the efficacy of siRNA‐mediated knockdown of *Atf2*. (P) *Atf2* knockdown abolished neomycin‐induced *Irp1* expression, as analyzed by dual luciferase assay. (Q,R) p‐ATF2 expression in cochlear explants after SAG treatment with indicated protein kinase inhibitors. MK‐2206, AKT inhibitor (AKTi). D4476, CK1 inhibitor (CK1i). Dasatinib, SRC inhibitor (SRCi). Dactolisib, PI3K inhibitor (PI3Ki). GSK180736A, GRK2 inhibitor (GRK2i). Bisindolylmaleimide II (BisII), PKC inhibitor (PKCi). H89, PKA inhibitor (PKAi). (S) Quantitative analysis of HCs after SAG treatment with or without indicated protein kinase inhibitors. (T) Quantitative analysis of HCs after RSL3 treatment with or without PKCi. Statistical analysis: one‐way analysis of variance (ANOVA) with Bonferroni's multiple comparison was employed for (F); two‐tailed Student's *t*‐test was employed for (L), (O), (P), (R) and (S); two‐way ANOVA with a post‐hoc Student Newman–Keuls test was employed for (B), (E), (N) and (T). ns means no significant difference, * *p *< 0.05, ** *p* < 0.01, *** *p* < 0.001, **** *p* < 0.0001.

As depicted above, the expression level of *Fpn* and *Cp* was downregulated after neomycin damage, which was restored by SMO inhibition (Figure 4M and Figure 8A). Thus, we questioned whether the otoprotective SMO inhibition targeted the FPN/CP system and altered iron efflux. Cochlear explants were challenged with neomycin following transfection with *Fpn* or *Cp* siRNA, with or without SANT‐1. Notably, following either *Fpn* or *Cp* knockdown, the protective effect of SANT‐1 against neomycin damage was diminished (Figure 8B; Figure S9). Previous studies have revealed that the ferroxidase activity of CP is required to stabilize FPN at the cell surface and that genetic deletion of *Cp* induces the rapid internalization and degradation of FPN [47, 48]. Therefore, in our experiments, it was rather difficult to distinguish the effect of knockdown of *Fpn* from that of *Cp* because knockdown of either one might induce a ‘knock‐out effect’ on the other. Taken together, these results suggest that inhibiting SMO restores FPN/CP expression to suppress iron overload in cochlear HCs.

Next, we sought to determine how neomycin and noise‐induced damage reduced FPN/CP expression and caused cytotoxic iron accumulation in HCs. How *Cp* expression is regulated is not quite clear, so we focused more on *Fpn*/FPN regulation, which is generally classified into two mechanisms, namely IRPs‐IRE‐mediated transcriptional regulation and hepcidin (encoded by *Hamp1*)‐triggered post‐translational regulation (Figure 8C) [12]. The iron‐responsive element (IRE), is a stem‐loop structure which can bind the iron regulatory protein (IRP). The IRE‐IRP interaction within 5′‐untranslated regions (UTRs) of *Fpn* blocks ribosome recruitment and subsequently represses *Fpn* transcription [49]. Note that neomycin‐activated SMO signaling also enhanced the iron influx process through upregulation of the ferritin receptor 1 (encoded by *Tfrc*), which is also regulated by IRPs. IRP‐IRE interaction within the 3`UTR of *Tfrc* mRNA protects the transcript from endoribonucleases‐mediated degradation [12]. There are two functionally similar IRPs, IRP1 (ACO1) and IRP2 (IREB2), and the expression of both IRPs was enhanced after neomycin and noise damage and was suppressed by SANT‐1 (Figure 8D–F; Figure S10A,B). Immunofluorescence analysis further revealed IRPs were regulated specifically in cochlear HCs (Figure S10A,B). IRPs have been reported to be regulated post‐translationally by FBXL5‐mediated degeneration in the ubiquitin‐proteasome pathway [50], but their transcriptional regulation is poorly studied. There were no apparent changes in the mRNA level of *Fbxl5* after neomycin or SANT‐1 treatment (Figure 8E), suggesting that increased IRPs levels were not due to decreased FBXL5‐mediated degeneration.

The next question was how SMO signaling increased IRPs expression. We then used the Genecards website and JASPAR database to search for potential transcriptional factor binding motifs in the *Irp1/2* gene promotor, and activating transcription factor 2 (ATF2) was predicted to be a co‐transcriptional factor for both *Irp1/2* genes (the predicted binding motif is shown in Figure 8H). Interestingly, ATF2 was found to be strongly activated in cochlear HCs after insults, but not in SCs, by detecting its phosphorylated form (Figure S10C,D). The expression of phosphorylated ATF2 (p‐ATF2) was enhanced after neomycin and was suppressed by SANT‐1(Figure 8G). Thus, we hypothesized that SMO might upregulate *Irp* expression through phosphorylated ATF2.

To elucidate the role of p‐ATF2 on *Irp1/2* expression, Cleavage Under Target & Tagmentation sequencing (CUT&Tag‐seq) analysis was then performed in the cultured HEI‐OC1 cells 24 h post‐neomycin stimulation to profile the genome‐wide DNA binding sites for p‐ATF2. A total of 10 197 peaks corresponding to 7879 RefSeq genes were identified by CUT&Tag‐seq, of which 60.75% were located at the promoter‐transcription start site (Figure S11A,B). Importantly, pathway enrichment analysis of the CUT&Tag‐seq data indicated that candidate transcription targets of p‐ATF2 were significantly enriched in the ferroptosis pathway (Figure S11C). The p‐ATF2 binding site was observed at the promoter region of the *Irp1* gene and not *Irp2* as identified by called peaks in CUT&Tag‐seq (Figure 8I,M). The results of a dual luciferase reporter assay further confirmed that p‐ATF2 overexpression evidently enhanced *Irp1*‐promoter‐mediated luciferase expression (Figure 8J–L,N). The called peak region mentioned above contains some DNA sequences with consensus to the predicted DNA‐recognition motif of p‐ATF2 (ATGAxGTCA) (Figure 8H,M). We then constructed a mutated *Irp1*‐promoter‐luciferase (△*Ireb1*‐promoter) plasmid by deleting the predicted p‐ATF2 binding site (ch4, 40143646‐40143658). △*Irp1*‐promoter abolished both ATF2 and p‐ATF2 overexpression‐induced luminescence enhancement (Figure 8N). To determine if ATF2 is required for the injury‐induced upregulation of *Irp1*, we knocked down *Atf2* expression in HEI‐OC1 cells using siRNA (Figure 8O). While neomycin robustly induced *Irp1* expression in control cells, this induction was significantly attenuated upon *Atf2* knockdown (Figure 8P), demonstrating that ATF2 is necessary for the transcriptional activation of *Irp1* in response to ototoxic stress. Together, these results suggest that p‐ATF2 is a novel transcription activator of *Irp1*.

As previously reported, the phosphorylation of ATF2 was mainly mediated by multiple protein kinases, and activation of SMO has been reported to stimulate different protein kinases, including PI3K, AKT, CK1, GRK2, SRC, PKA, and PKC [51, 52, 53, 54]. We applied specific inhibitors of these protein kinases to determine which kinase mediated SMO activation‐induced ATF2 phosphorylation and subsequent IRP signaling. We found that only bisindolylmaleimide II (BisII), a PKC family inhibitor, abolished the SAG‐induced ATF2 phosphorylation (Figure 8Q,R) and HC death (Figure 8S). Moreover, PKCi ameliorated neomycin and RSL3‐induced HC death (Figure 8T). Taken together, these results suggest that the PKC/p‐ATF2 axis is involved in SMO activation‐induced IRP1 overexpression and iron accumulation in cochlear HCs.

### 3.6 SMO Inhibition Suggests the Clinical Potential for Treating Aminoglycosides and Noise‐Induced Hearing Loss

**FIGURE 9:**
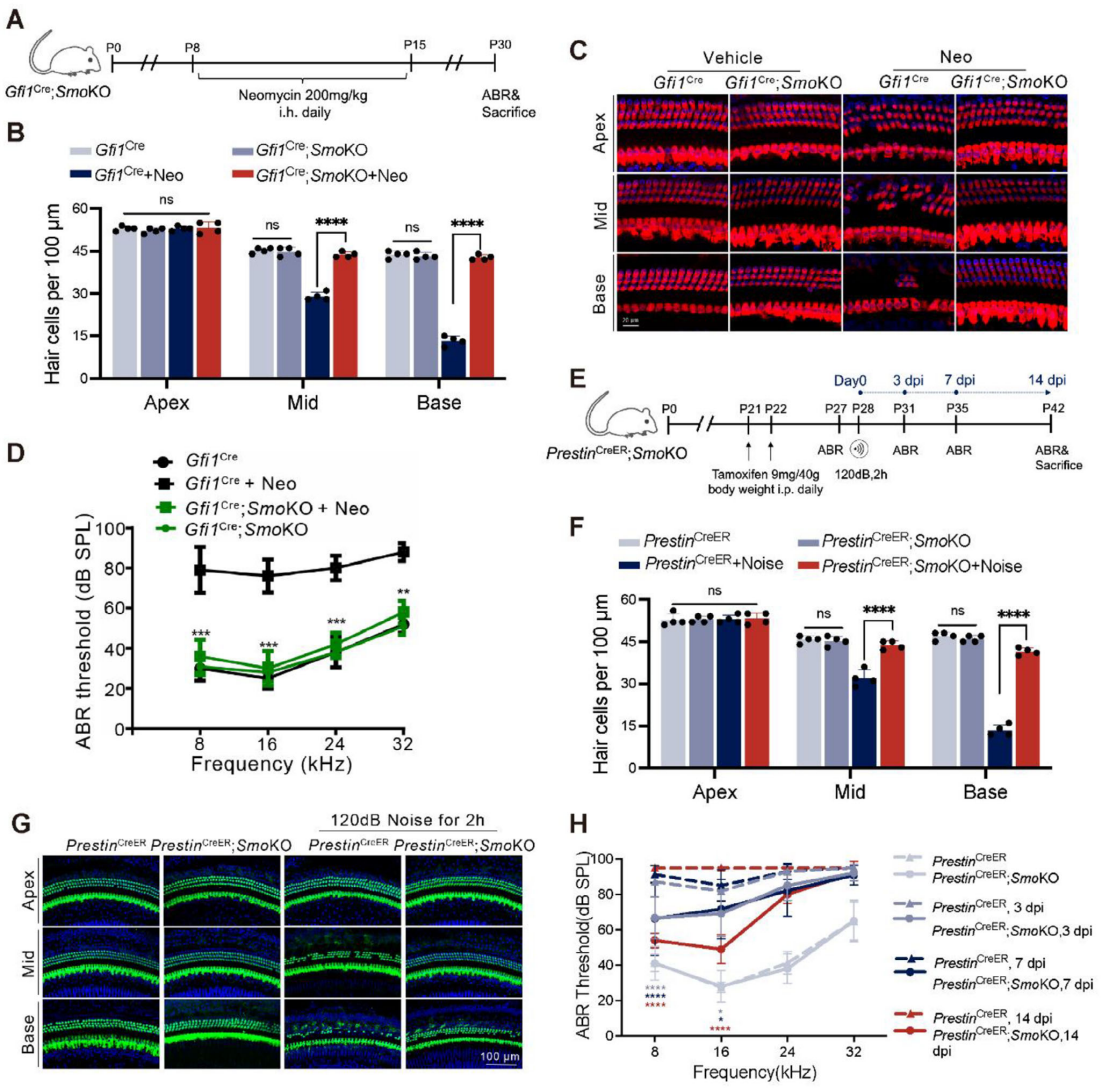
SMO inhibition ameliorated aminoglycosides and noise‐induced hearing loss. (A) Experimental design of B–D. (B,C) Quantitative analysis and representative images of cochlear HCs labeled with Myo7a (green) from *Gfi1* ^Cre^ or *Gfi1* ^Cre^/*Smo*KO mice with or without neomycin damage. (D) ABR thresholds of *Gfi1* ^cre^ and *Gfi1* ^cre^/*Smo*KO mice after neomycin damage. (E) Experimental design for F–H. (F,G) Quantitative analysis and representative images of cochlear HCs from *Prestin* ^creER^ and *Prestin* ^creER^/*Smo*KO mice with or without acoustic trauma. (H) ABR thresholds of *Prestin* ^creER^ and *Prestin* ^creER^/*Smo*KO mice after acoustic trauma. At least 5 mice were used in each group in (D) and (H). Statistical analysis: a two‐tailed Student's *t*‐test was employed for (D) and (H). The statistical significance was analyzed for the ABR threshold of each independent frequency between *Gfi1* ^Cre^; *SmoKO* and *Gfi1* ^Cre^; *SmoKO* +Neo, *Gfi1* ^Cre^ and *Gfi1* ^Cre^ +Neo in (D), and between *Prestin* ^CreER^ and *Prestin* ^CreER^; *SmoKO* in (H); two‐way ANOVA with a post‐hoc Student Newman–Keuls test was employed for (B) and (F). ns means no significant difference, * *p *< 0.05, ** *p* < 0.01, *** *p* < 0.001, **** *p* < 0.0001.

We next tested whether SMO inhibition could protect mice from aminoglycosides or noise‐induced hearing loss. As depicted above, genetic ablation of *Smo* protected neonatal cochlear HCs from neomycin ototoxicity. This observation was further supported by in vivo results (Figure 9A–C). After the neomycin challenge, *Gfi1* ^Cre^/*Smo*KO mice exhibited significantly better hearing function, as demonstrated by reduced auditory threshold shifts at all tested frequencies compared to control mice (Figure 9D).

We also employed a noise‐induced hearing loss model to further explore the otoprotective effect of SMO inhibition in adulthood. Given that cochlear OHCs are much more sensitive to acoustic trauma than IHCs, we generated *Prestin* ^CreER^/*Smo* ^2Amc/2Amc^ (*Prestin* ^CreER^/*Smo*KO) mouse line, in which *Smo* was specifically targeted for deletion in OHCs at P21/P22, an age when cochlear HCs had fully matured. *Prestin* ^CreER^ mice lacking the *Smo* ^2Amc^ allele were used as controls. HC loss from noise damage can continue for up to 10 days, and thus we measured permanent threshold shifts at 14 days post injury (dpi) (Figure 9E). *Prestin* ^CreER^/*Smo*KO mice exhibited significantly lower hearing thresholds at 3, 7, and 14 dpi as compared to control mice (Figure 9H), and HC loss was reduced accordingly in the *Prestin* ^CreER^/*Smo*KO group (Figure 9F,G). Therefore, inhibiting SMO not only ameliorated noise‐induced hearing loss in the acute phase (3 and 7 dpi), but also improved the ultimate hearing outcomes (14 dpi). Taken together, these results suggested that HC‐specific *Smo* knockout alleviated aminoglycoside and noise‐induced hearing loss.

**FIGURE 10:**
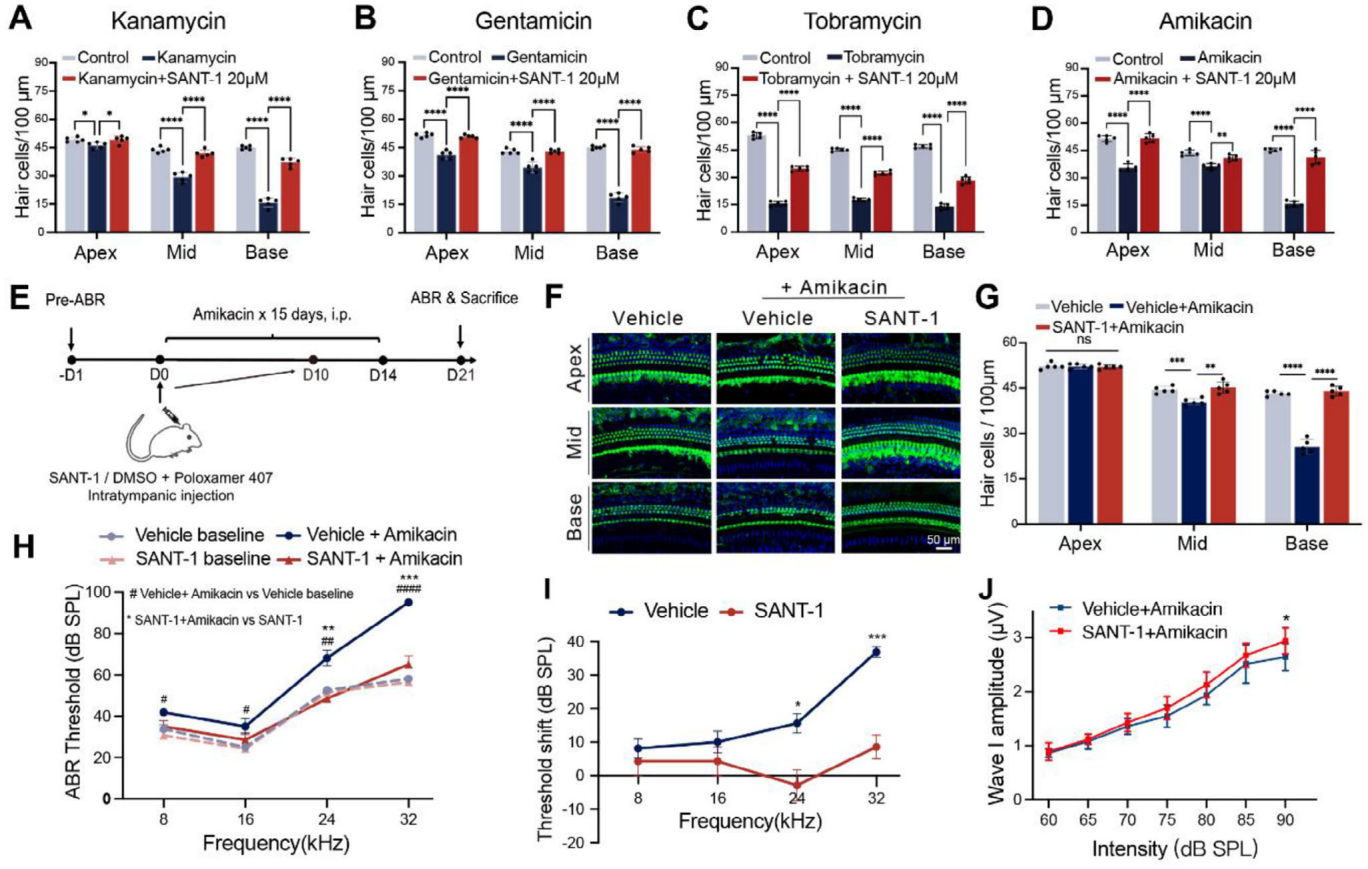
SMO inhibition showed clinical potential for treating aminoglycoside‐induced hearing loss. (A–D) Quantitative analysis of HCs in cochlear explants after kanamycin (A), gentamicin (B), tobramycin (C), and amikacin (D) damage with or without SANT‐1. (E) Experimental design for F–J. (F,G) Representative images (F) and quantitative data (G) of Myo7a^+^ cochlear HCs from amikacin‐treated mice with or without SANT‐1. (H,I) ABR threshold (H) and threshold shift (I) at the indicated group. (J) Wave I amplitude at 16 kHz in amikacin‐treated mice with or without SANT‐1. Statistical analysis: two‐tailed Student's *t*‐test was employed for (H–J); two‐way ANOVA with a post‐hoc Student Newman–Keuls test was employed for (A–D) and (G). ns means no significant difference, */^#^ *p *< 0.05, **/^##^ *p* < 0.01, ***/^###^ *p* < 0.001, ****/^####^ *p* < 0.0001.

SMO inhibitors have been widely used in the treatment of Hh‐driven cancer types like medulloblastoma [55], basal cell carcinoma [56], and acute myeloid leukemia [57]. Since the safety of various SMO inhibitors has been comprehensively studied in humans, it is worth evaluating their potential for treating hearing loss. As mentioned in Figure 4C, SANT‐1 exhibited the best otoprotective profile in vitro among all SMO inhibitors. Further, SANT‐1 showed a broad‐spectrum protective effect against a series of clinically widely‐used aminoglycosides, including kanamycin, tobramycin, gentamicin, and amikacin (Figure 10A–D), suggesting that SANT‐1 is a potent therapeutic candidate for preventing aminoglycoside ototoxicity in the clinic.

To further evaluate the protective effects of SANT‐1 against aminoglycoside‐induced HC death and hearing loss in adult mice, we established the amikacin insult model by administering intraperitoneal injection of amikacin for 15 consecutive days at a dose of 500 mg/kg (Figure 10E). Considering the potential adverse effects of systemically‐delivered SMO inhibitors such as dysgeusia, muscle spasm, and alopecia, we adopted trans‐tympanic injection for SANT‐1 delivery to minimize such toxicity. At 7 days after the final injection of amikacin, we observed auditory threshold elevations of 7–37 dB at all tested frequencies (8, 16, 24, and 32 kHz). Mice co‐treated with SANT‐1 exhibited significantly reduced threshold shifts, with a mean reduction of ∼18 and ∼28 dB at frequencies of 24 and 32 kHz, respectively (Figure 10H,I). Likewise, the amplitudes of the ABR wave 1 at 16 kHz (90 dB stimulus level) were significantly higher in the SANT‐1‐injected mice (Figure 10J). Morphological analysis of the cochlea showed that SANT‐1 ameliorated amikacin‐induced HC loss in the middle and basal turns (Figure 10F,G). Together, both ABR tests and HC morphological measurements demonstrated that SANT‐1 reduced aminoglycoside‐induced hearing loss in adult mice. According to the clinical trial guidelines for otoprotectants, tested compounds reducing hearing loss by at least 20 dB at a given frequency or by at least 10 dB at any two adjacent frequencies can be considered effective [22, 58]. Given that the robust protection effect conferred by trans‐tympanically‐delivered SANT‐1 was as high as 28 dB at multiple frequencies, our finding that SMO inhibitors can act as otoprotectants suggests that such compounds can significantly advance the clinical prevention and treatment of aminoglycoside‐induced ototoxicity in patients.

## 4 Discussion

The major conclusion of this work is that activation of SMO leads to increased iron overload, which exacerbates aminoglycosides and noise induced hearing impairment, while inhibiting SMO shows promising otoprotective effects. We provided the following pieces of evidence to support our conclusion: First, *Smo*/SMO was upregulated and activated in cochlear HCs, quickly after injury, prior to apparent HC death. Second, genetic ablation or pharmaceutical inhibition of SMO protected cochlear HCs and hearing function. Third, SMO inhibition restored iron homeostasis and thus suppressed HC ferroptosis by regulating iron efflux and influx via the PKC‐ATF2‐IRP1‐FPN/TFRC axis (Figure 1).

Besides serving as an important developmental morphogen, previous research has also shown that SMO signaling affects postnatal organ homeostasis by regulating stem cell self‐renewal and injury‐dependent regeneration, as evidenced by DHH‐promoted angiogenesis after ischemia [59] and SHH‐accelerated wound healing in diabetic mice [60]. Note that most studies on postnatal SMO functions focus on proliferative and differentiating cells, such as stem cells and tumor cells, while the role of SMO in terminally differentiated cells is poorly elucidated. In this work, we reported for the first time that neomycin and acoustic injury induced apparent SMO upregulation and activation in HCs, exacerbating iron overload toxicity and subsequent ferroptosis. Similarly, an earlier work reports that activation of SMO inhibits GLT‐1, a glutamate transporter, and aggravates glutamate toxicity in neurons [41]. The effect of SMO activation could be time‐specific, tissue‐specific, and cell‐type specific. Understanding the diverse pathological conditions that arise from aberrant SMO activation requires careful consideration of these distinctions.

An important observation in this study was that SMO regulated iron homeostasis through the IRP1‐FPN/TFRC axis in cochlear HCs. Dysregulated iron metabolism is closely associated with hearing loss. Mitochondrial iron overload and dysregulated iron homeostasis are speculated as the pathological mechanisms behind mutated FDXR‐induced auditory neuropathies [61]. Our work provides direct evidence showing that iron overload in cochlear HCs contributes to aminoglycoside and noise‐induced hearing loss, which are two very common acquired hearing deficits. This is also the first report revealing the important role of SMO signaling in iron homeostasis regulation. SMO inhibition significantly upregulated FPN‐mediated iron efflux and downregulated TFRC‐mediated iron influx through downregulation of IRPs. In addition, a comprehensive picture of the iron metabolism in cochlear HCs, including iron influx, iron storage (ferritin and autophagic degradation), and iron efflux, is provided in this study, thus laying the groundwork for more comprehensive studies on iron regulation in the cochlea, which still remains poorly understood.

Another question that intrigued us was how SMO signaling increased *Irp* expression. We adopted two transcriptional factor prediction models to search for potential *Irp* regulators, among which ATF2 was found to be activated in HCs upon injury and inactivated by SMO inhibition. Previous work implies the dual role of ATF2 in regulating cell death in different cell types, and this is supported by the reports that p‐ATF2 inhibits gastric cancer cell death [62] while promoting neuronal cell death after mechanical damage [63]. According to our results, p‐ATF2 upregulated *Irp1* expression and aggravated iron overload toxicity in cochlear HCs, which was activated by SMO in a manner dependent of PKC. Some questions remain not fully addressed here. First, our results could only identify p‐ATF2 as a downstream mediator for SMO‐dependent iron metabolism regulation, and more comprehensive analyses are needed to verify whether p‐ATF2 as the major mediator and whether other molecules are also involved. Second, despite the fact that early studies have reported PKC as the upstream activator of ATF2 [64, 65], additional evidence is needed to confirm that PKC directly binds to and phosphorylates ATF2 in cochlear HCs. Third, it is unclear which subtype of PKC is involved in the SMO‐dependent ATF2 phosphorylation. Pharmacological screening with isoform‐selective inhibitors pointed to PKCδ as a candidate (Figure S12). However, given the potential lack of absolute specificity of these pharmacological tools, future studies employing genetic knockout of specific PKC isoforms in HCs are essential to conclusively define the isoform‐specific requirements for SMO‐mediated HC death.

Interestingly, our study suggests a novel, non‐canonical role for SMO in mediating cochlear HC death, distinct from its well‐established function in SHH‐dependent developmental patterning. We found that SMO was activated in cochlear HCs by aminoglycosides and noise exposure. In the canonical Hedgehog signaling, SMO activation requires the presence of the Hedgehog ligand to relieve the inhibition by PTCH. However, previous studies have demonstrated that *Shh* expression in the mouse cochlea is below the detectable level after birth [37]. Furthermore, our results showed that *Shh* mRNA was not upregulated in the cochlea upon damage (Figure 5B,D), suggesting that SMO activation might be independent of SHH. Consistent with SMO activation, we observed a significant increase in SMO localization to primary cilia and the cell membrane in HEI‐OC1 cells following neomycin injury (Figure 5G,H,J–L). However, the downstream transcriptional output of the canonical Hedgehog pathway remains silent (Shh ligand was not induced, and target genes such as *Gli1 and Ptch1* were not upregulated, Figure 5B,D). These results imply that the activated SMO signals through a non‐canonical, SHH‐independent pathway​ to mediate HC death, a mechanism that requires further exploration.

Sensorineural hearing loss is a widespread and debilitating condition that severely impacts quality of life. According to the World Health Organization, over 1.5 billion people worldwide suffer from hearing impairment [2]. Building on our current findings and on previous work, we propose targeting iron homeostasis as a new and promising approach. However, clinical trials regarding iron chelator therapy in beta‐thalassemia report that DFO may cause hearing loss due to its neurotoxicity [10]. Importantly, our work suggests that SMO is a novel druggable target for relieving iron ototoxicity. In this work, we screened six different SMO inhibitors, among which SANT‐1 exhibited the best otoprotective profile. The safety and effectiveness of SANT‐1 should be further tested in primate models before proceeding to clinical trials.

## Author Contributions

Yan Chen, Huawei Li, and Wenyan Li conceived the experiments. Huanyu Mao, Xiang Li, and Yaqi Liao performed the majority of experiments and acquired the data. Liman Liu, Xian Gao, and Hailiang Lin performed the in vitro cochlear explant culture. Wenli Ni bred the mice. Huanyu Mao and Yan Chen analyzed the data and wrote the paper. Huawei Li, Yan Chen, and Wenyan Li revised the paper. All authors read and approved the final manuscript.

## Funding

This work was supported by the National Key R&D Program of China (No. 2025YFC3508205), the National Natural Science Foundation of China (Nos. 82425018, 82271170, 82192861, 82271159), the STI2030‐Major Projects (No. 2022ZD0205400), and the Key Research and Development Program of Zhejiang Province (2024C03238).

## Conflicts of Interest

The authors declare no conflicts of interest.

## Supporting information




**Supporting File 1**: advs74603‐sup‐0001‐SuppMat.docx.

**Supporting File 2**: advs74603‐sup‐0002‐DataSet.docx.

## Data Availability

The data that support the findings of this study are available from the corresponding author upon reasonable request.
