# Supplementary material for: Restoring Iron Homeostasis via Smoothened Inhibition: A Novel Strategy Against Hearing Loss
Source: Adv Sci (Weinh). 2026 Apr 17;13(29):e20749. doi: 10.1002/advs.202520749 (PMC13205593; doi:10.1002/advs.202520749)
Supplement: Supplementary file 1 — Supporting File 1: advs74603‐sup‐0001‐SuppMat.docx. [file ADVS-13-e20749-s001.docx]

**Supplementary Materials**

**Restoring Iron Homeostasis via Smoothened Inhibition: A Novel Strategy Against Hearing Loss**

Huanyu Mao ^a,b*^, Xiang Li ^a,b*^, Yaqi Liao ^a,b,c*^, Liman Liu ^e^, Xian Gao^a,b^ , Hailiang Lin^a,b^, Wenli Ni ^a,b^, Huawei Li ^a,b,d†^, Yan Chen ^a,b†^, Wenyan Li ^a,b,d†^

^a^ ENT Institute and Otorhinolaryngology Department of Eye & ENT Hospital, State Key Laboratory of Medical Neurobiology and MOE Frontiers Center for Brain Science, Institutes of Biomedical Sciences, Fudan University, Shanghai, 200031, PR China

^b^ NHC Key Laboratory of Hearing Medicine (Fudan University), Shanghai, 200031, PR China

^c^ Department of Otorhinolaryngology Head and Neck Surgery, Wuhan Third Hospital, Tongren Hospital of Wuhan University, Wuhan, 430030, PR China

^d^ The Institutes of Brain Science and the Collaborative Innovation Center for Brain Science, Fudan University, Shanghai, 200032, PR China

^e^ Department of Surgery–Otolaryngology, Yale University Medical School, 310 Cedar Street, New Haven, CT, 06511, USA.

^*^ These authors contributed equally.

^†^ Correspondence authors. E-mail address(es): wenyan_li@fudan.edu.cn (Wenyan Li, Tel: +86 13761735615)

; chenyan0528@fudan.edu.cn (Yan Chen); hwli@shmu.edu.cn (Huawei Li).

**List of Supplementary Materials**

Fig. S1 to S12 for multiple supplementary figures.

Table S1 for one supplementary table

**Supplementary Figure1**


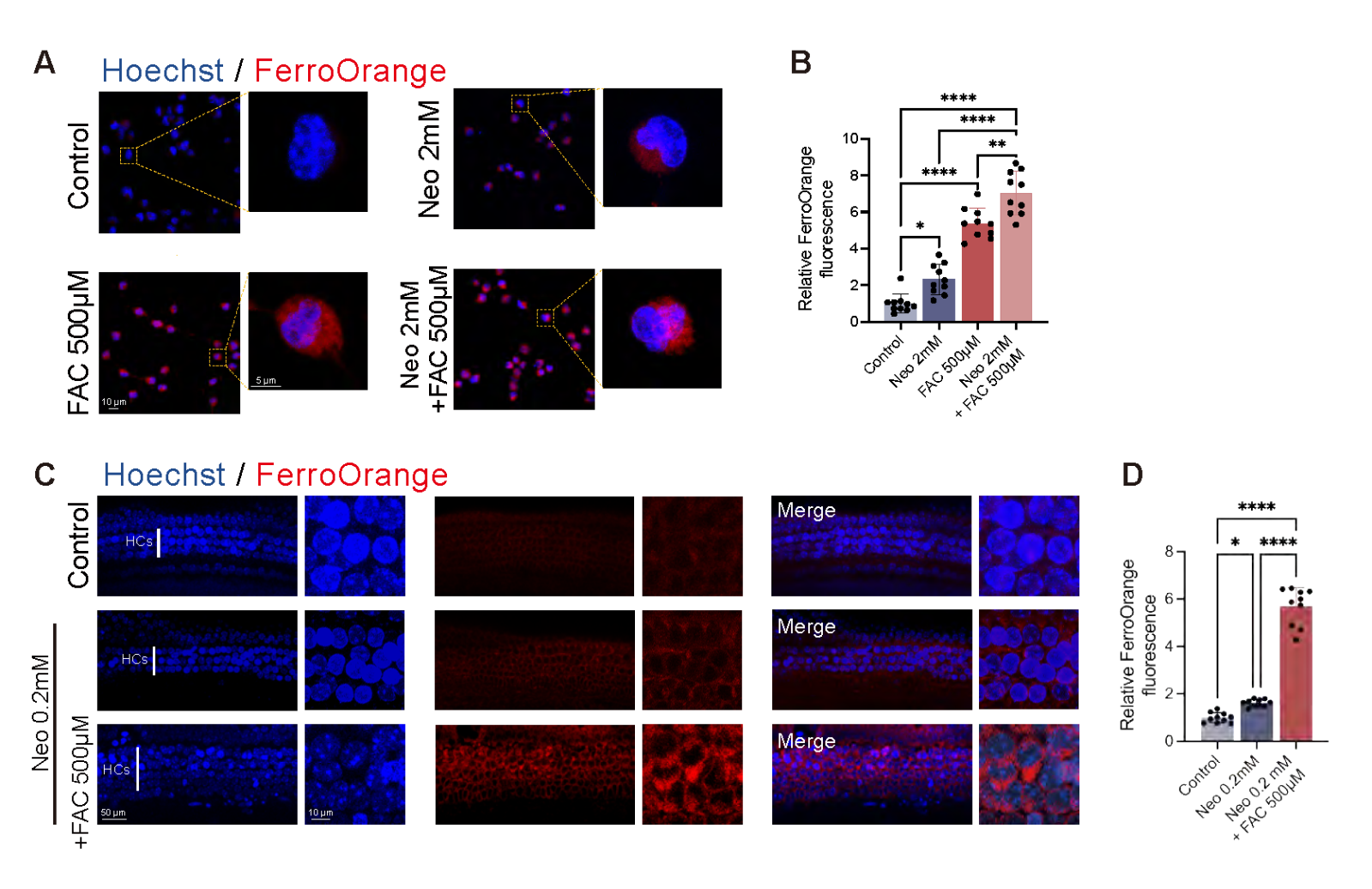


**Figure S1 FAC aggravated neomycin induced iron accumulation in both HEI-OC1 cells and cochlear HCs. Related to Figure 3.** Representative images and quantitative analysis of FerroOrange staining (red) in HEI-OC1 cells (A and B) and cochlear HCs (C and D) at the indicated groups. Statistical analysis: two-way ANOVA with a post-hoc Student Newman-Keuls test was employed for (B) and (D). ns means no significant difference, * *p*< 0.05, ** *p* < 0.01, *** *p* < 0.001, **** *p* < 0.0001.

**Supplementary Figure2**


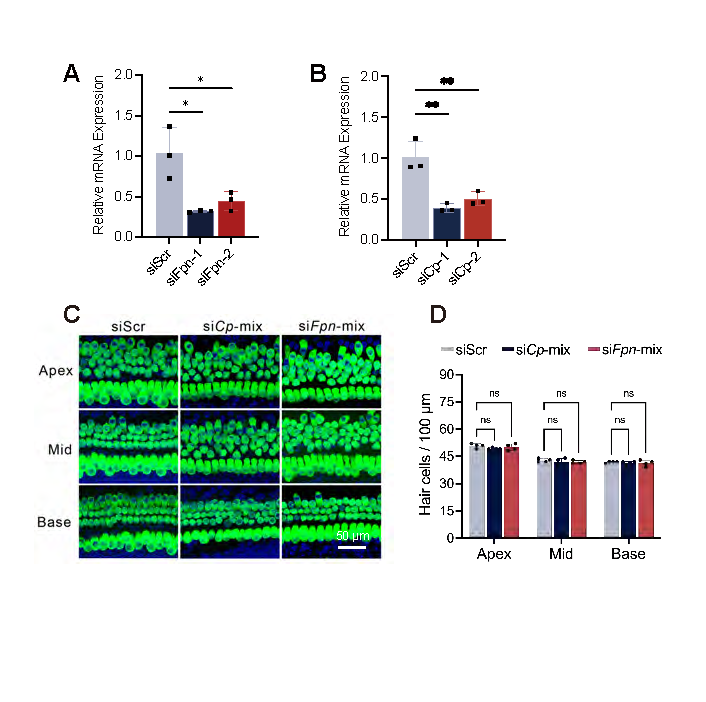


**Figure S2 Transfection with either si*Fpn* or si*Cp* alone did not affect hair cell survival in the absence of neomycin. Related to Figure 3.** (A) qPCR analysis of si*Fpn* efficacy. (B) qPCR analysis of si*Cp* efficacy. (C and D) Representative images and quantitative analysis of cochlear HCs after transfected with different siRNAs. Statistical analysis: two-tailed Student’s t-test was employed for (A), (B) and (D). ns means no significant difference, * *p*< 0.05, ** *p* < 0.01, *** *p* < 0.001, **** *p* < 0.0001.

**Supplementary Figure3**


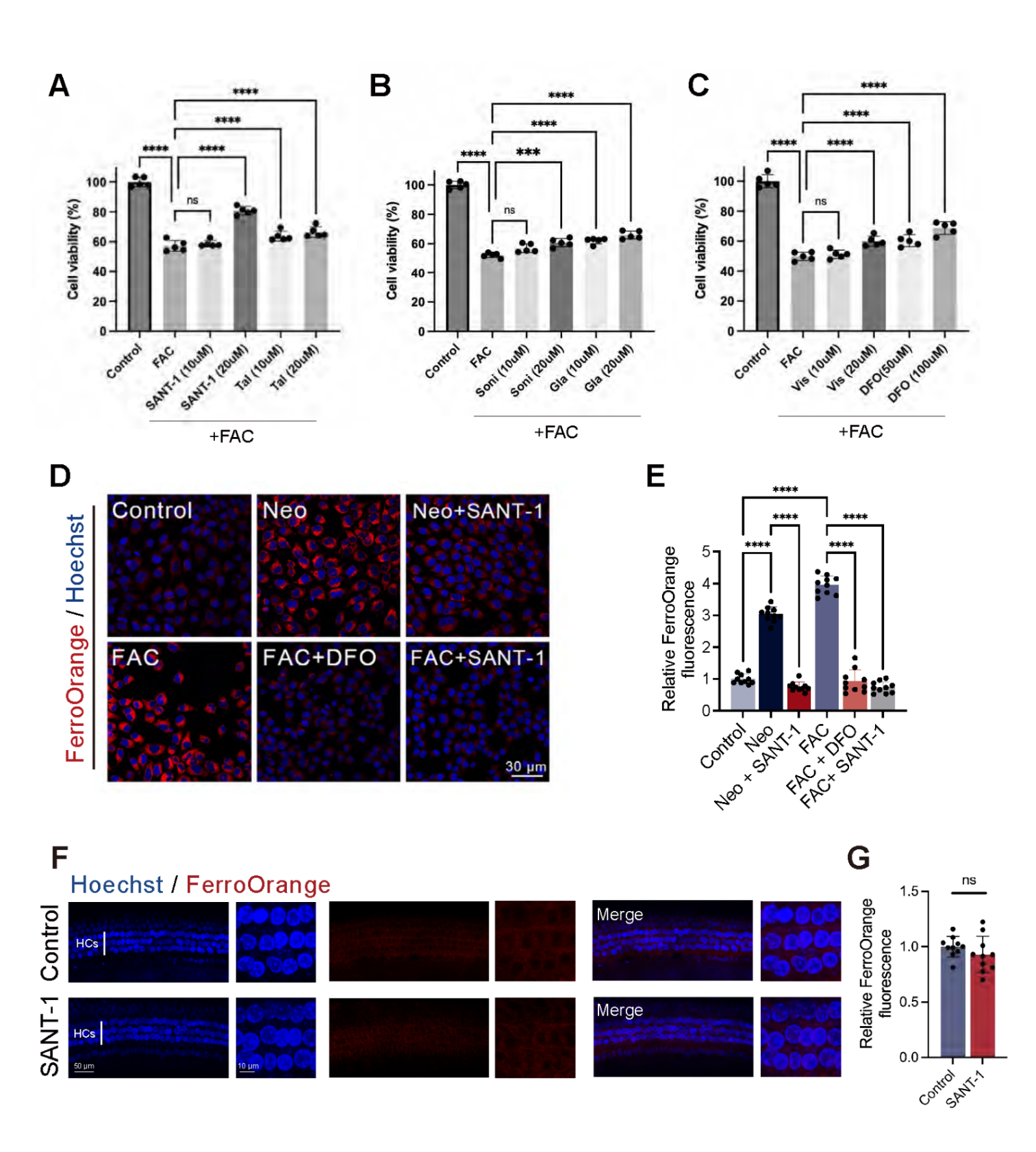


**Figure S3 SMO antagonists ameliorated iron overload-induced HEI-OC1 cell death.** **Related to Figure 4.** (A-C) Analysis of HEI-OC1 cell viability after incubating with FAC and SMO antagonists or DFO for 24 hours by CCK8 assay. (D-E) Representative images (D) and quantitative analysis of FerroOrange staining (red) in HEI-OC1 cells from the indicated group. (F-G) Representative images (F) and quantitative analysis (G) of FerroOrange staining (red) in cochlear HCs with or without SANT-1 treatment. Statistical analysis: two-tailed Student’s t-test was employed for (G); two-way ANOVA with a post-hoc Student Newman-Keuls test was employed for (A-C) and (E). ns means no significant difference, * *p*< 0.05, ** *p* < 0.01, *** *p* < 0.001, **** *p* < 0.0001.

**Supplementary Figure4**


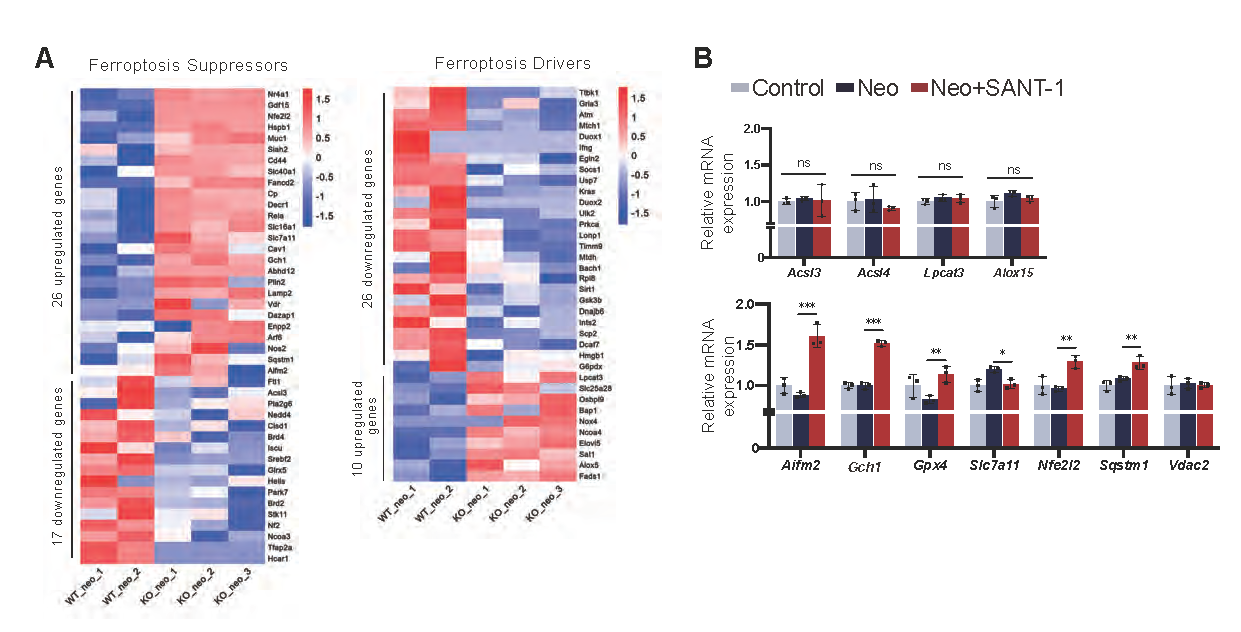


**Figure S4 RNA-sequencing and qPCR analysis revealing the effect of SMO inhibition on ferroptosis-related genes.** **Related to Figure 4.** (A) Heatmap displaying differentially expressed genes of ferroptosis pathway. (B) qPCR validation of differentially expressed genes. wt_neo, cochlear HCs of *Gfi1*^Cre^;*ROSA26^CAG-tdTomato^* mice after neomycin damage; ko_neo, cochlear HCs of *Gfi1*^Cre^*;Smo*KO*;ROSA26^CAG-tdTomato^* mice after neomycin damage. Statistical analysis: two-tailed Student’s t-test was employed for (B). ns means no significant difference, * *p*< 0.05, ** *p* < 0.01, *** *p* < 0.001, **** *p* < 0.0001.

**Supplementary Figure5**


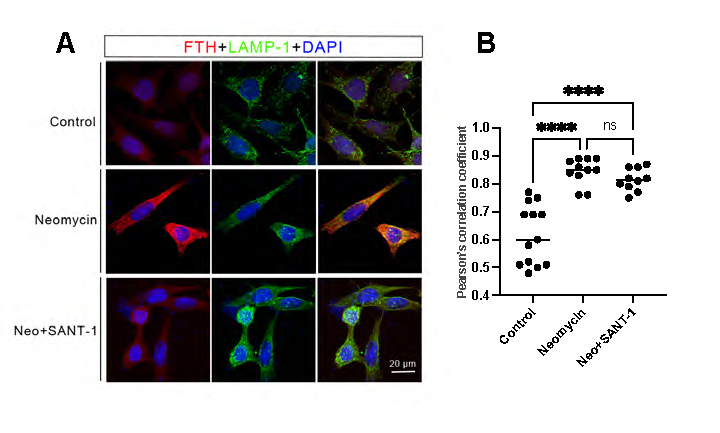


**Figure S5 The effect of SANT-1 on ferritinophagy. Related to Figure 4.** (A) Representative images of FTH (red) and LAMP-1 (green) staining in HEI-OC1 cells at the indicated conditions. (B) Quantitative analysis of Pearson`s correlation coefficient between FTH and LAMP-1 staining. Statistical analysis: two-way ANOVA with a post-hoc Student Newman-Keuls test was employed for (B). ns means no significant difference, * *p*< 0.05, ** *p* < 0.01, *** *p* < 0.001, **** *p* < 0.0001.

**Supplementary Figure6**

**Figure S6 SMO expression pattern in the cochlear sensory epithelium. Related to Figure 5.** (A) Representative images of SMO staining (red) in the cochlear sensory epithelium from P14 and P30 C57BL/6 wide-type mice. HCs were labeled with Parvalbumin (green) and SCs were labeled with SOX2 (white). (B) Individual optical sections of Figure 5E (neomycin-treated group), demonstrating signal distribution across adjacent hair cells within the section. (C and D) Representative images (C) and quantitative analysis (D) of SMO immunofluorescence in the organ of Corti with or without neomycin injury. Statistical analysis: two-tailed Student’s t-test was employed for (D). ns means no significant difference, * *p*< 0.05, ** *p* < 0.01, *** *p* < 0.001, **** *p* < 0.0001.

**Supplementary Figure7**


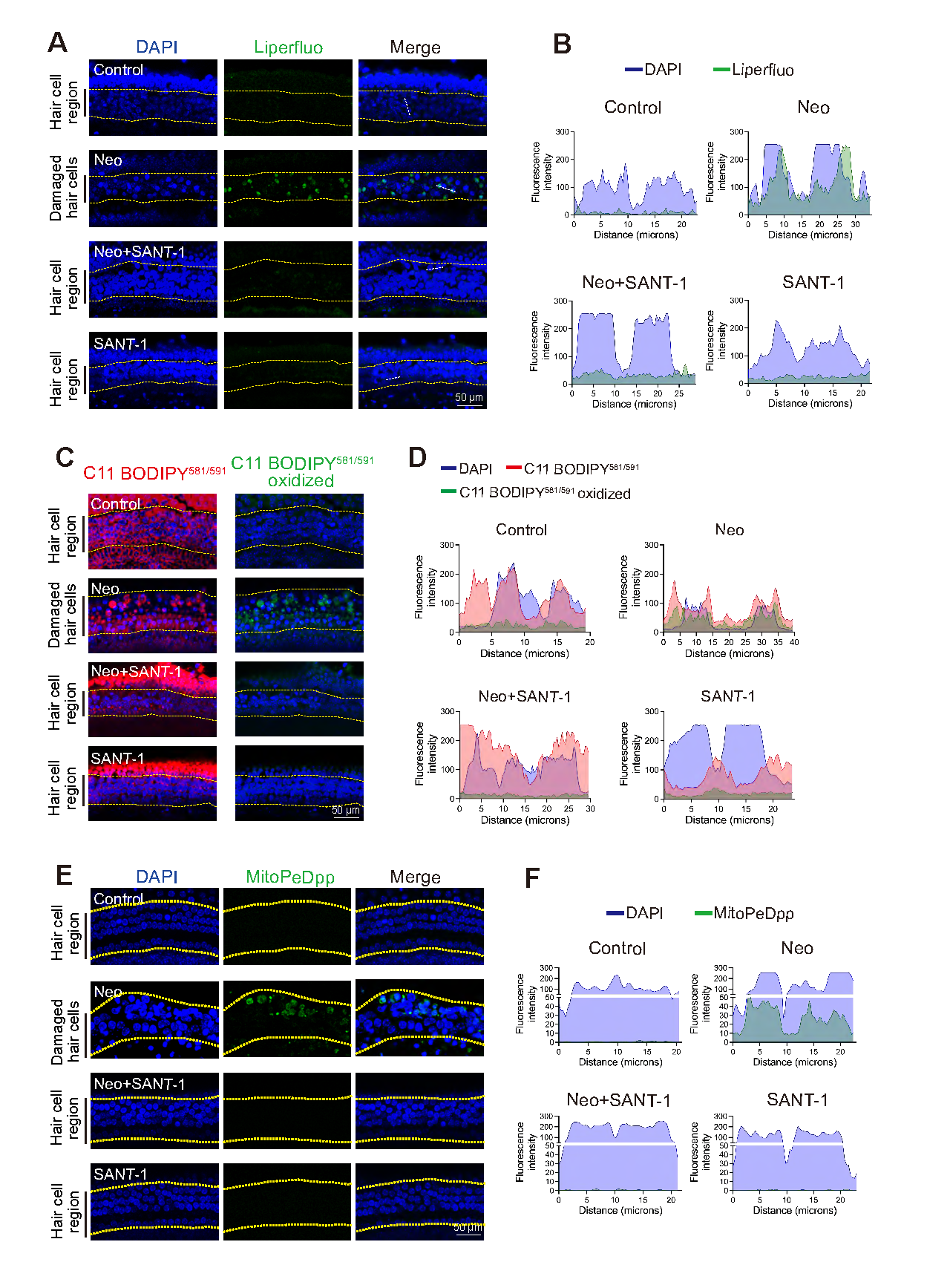
**Figure S7 SANT-1 ameliorated neomycin-induced lipid peroxides accumulation in cochlear HCs. Related to Figure 7.** Detection of lipid peroxides by Liperfluo (A and B), C11 BODIPY581/591 (C and D), and MitoPeDpp (E and F).

**Supplementary Figure8**

**
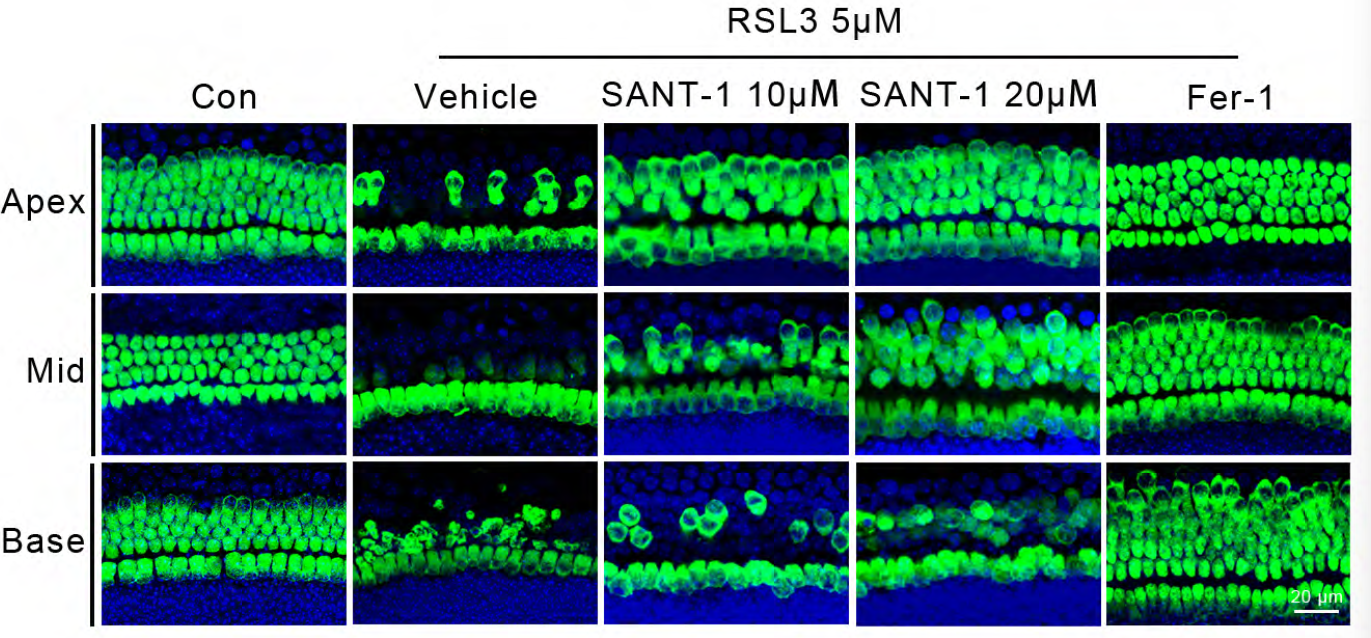
**

**Figure S8 SANT-1 protected cochlear HCs from RSL3-induced ferroptosis. Related to Figure 7.** Representative images of cochlear HCs after RSL3 treatment with or without Fer-1 and SANT-1.

**Supplementary Figure9**


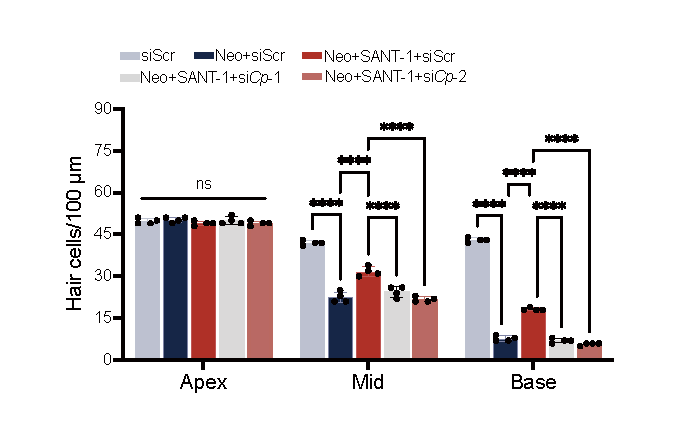


**Figure S9 Cp knockdown abolishes SANT-1 protection against neomycin injury. Related to Figure 8.** Statistical analysis: two-way ANOVA with a post-hoc Student Newman-Keuls test was employed for Fig.S9. ns means no significant difference, * *p*< 0.05, ** *p* < 0.01, *** *p* < 0.001, **** *p* < 0.0001.

**Supplementary Figure10**


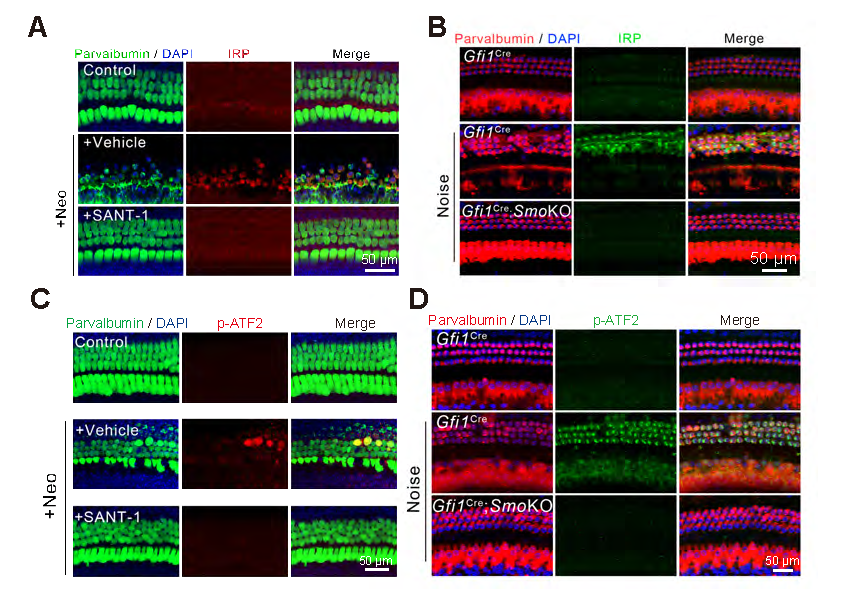


**Figure S10** **SMO inhibition suppressed neomycin- and noise-induced IRP upregulation and ATF2 phosphorylation in cochlear HCs.** **Related to Figure 8.** (A and B) Representative images of IRPs staining in neomycin-treated (A) and noise-damaged (B) cochlear sensory epithelia. (C-D) Representative images of p-ATF2 staining in neomycin-treated (C) and noise-damaged (D) cochlear sensory epithelia. HCs were labeled with Parvalbumin.

**Supplementary Figure11**


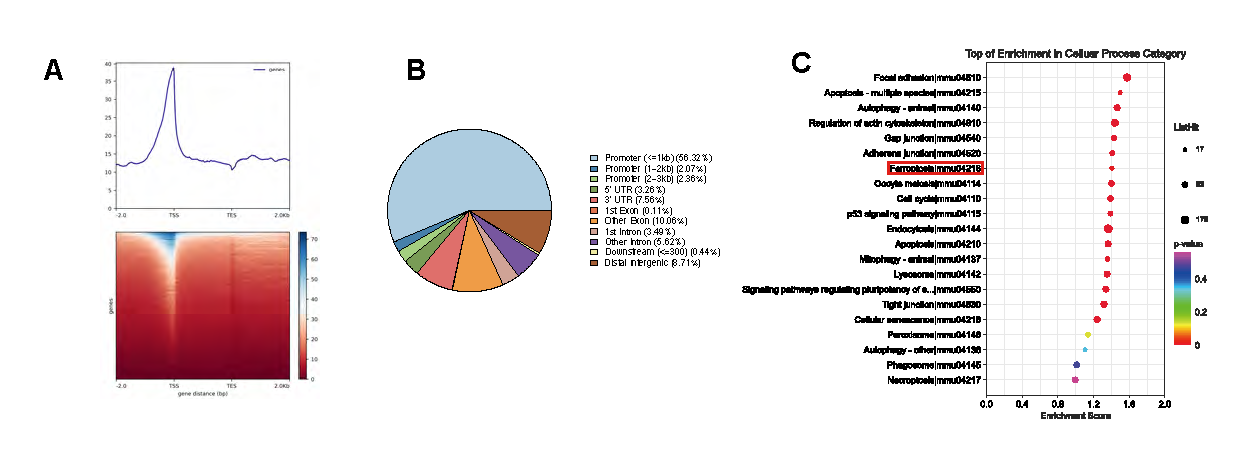


**Figure S11 Analysis results of the CUT&TAG-seq data.** **Related to Figure 8.** (A) Distribution of reads across all gene regions. The x-axis shows the TSS (TES) ± 2000bp region, while the y-axis shows the mean read abundance. TSS, transcription start site. TES, transcription end site. (B) Annotation plot of peaks detected in gene functional elements. A total of 60.75% of the detected peaks are located within gene promoter regions. (C) GO enrichment analysis of peak-associated genes.

**Supplementary Figure12**


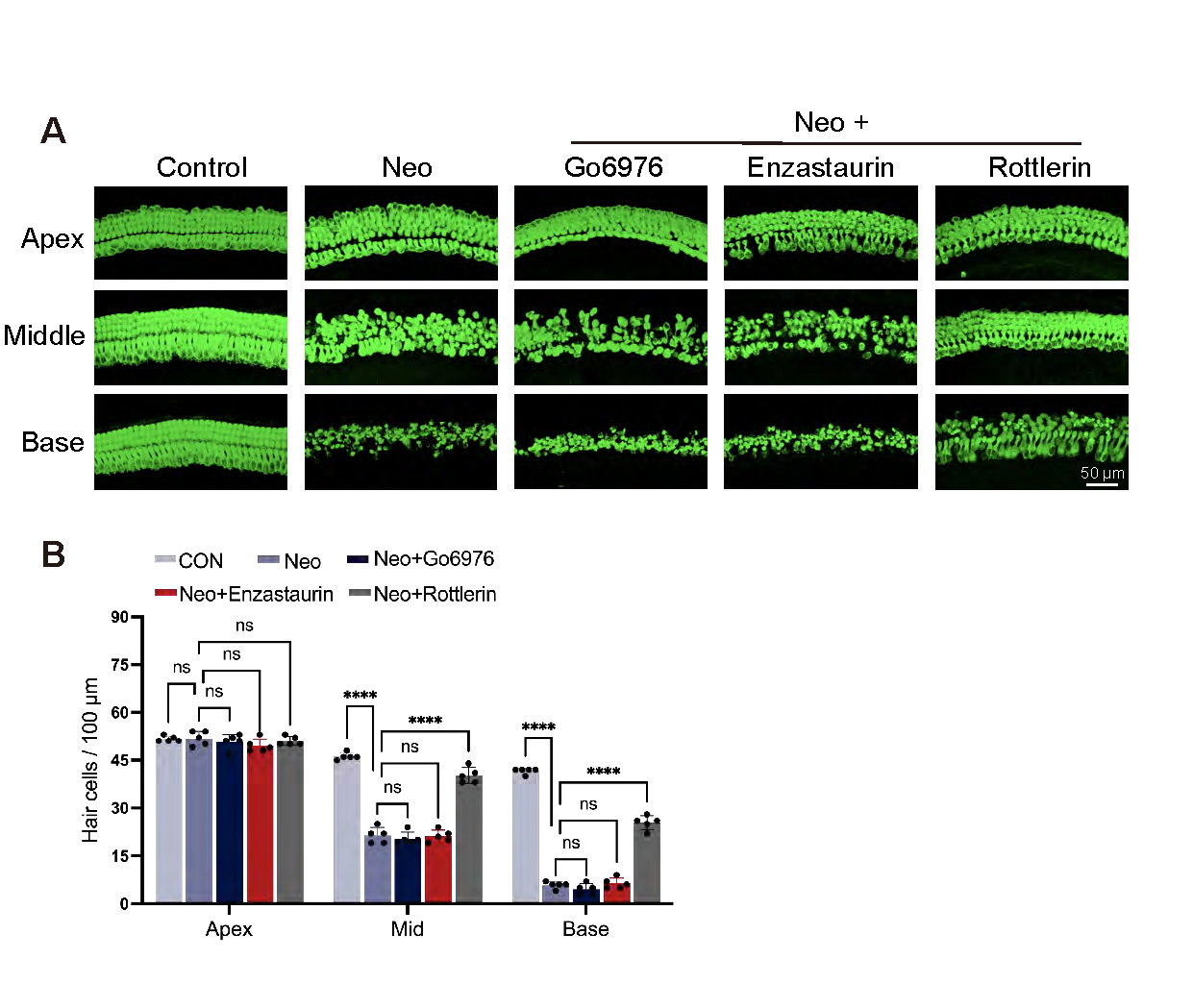


**Figure S12 Rottlerin ameliorated neomycin ototoxicity. Related to Figure 8.** (A and B) Representative images (A) and quantitative analysis (B) of HCs after neomycin treatment with or without indicated isoform-selective inhibitors of PKC. Go6976: An inhibitor of PKCα and PKCβ. Enzastaurin: a selective inhibitor of PKCβ. Rottlerin: a selective inhibitor of PKCδ. Statistical analysis: two-way ANOVA with a post-hoc Student Newman-Keuls test was employed for (B). ns means no significant difference, * *p*< 0.05, ** *p* < 0.01, *** *p* < 0.001, **** *p* < 0.0001.

**Table S1. The oligonucleotide primer sequences**

| Gene | Oligonucleotide primer sequences | |
| --- | --- | --- |
| *Smo* | F | 5′CTGTGTGCTGTCTACATGCC3′ |
|  | R | 5′AGCTCTTGGGGTTGTCTGTT3′ |
| *Shh* | F | 5′CGGCAGGCAAGGTTATATAGG3′ |
|  | R | 5′CCTGAGGACTTGTGAGCTGT3′ |
| *Ptch1* | F | 5′TTCAGACTCCAAAAGAAGAAGGCG3′ |
|  | R | 5′CAAAACAAGGGCCACATCAAGAG3′ |
| *Gli1* | F | 5′CCTGTTGGGGATGCTGGAT3′ |
|  | R | 5′CTGCTCCTGGGAATCGAACT3′ |
| *Tfrc* | F | 5′TGATTGGATTCATGAGTGGCT3′ |
|  | R | 5′GGTCTGCCCAATATAAGCGAG3′ |
| *Trf* | F | 5′CGCAGTCCTCTTGAGAAAGC3′ |
|  | R | 5′CACCGCCATCTTTCAGACAC3′ |
| *Fth* | F | 5′GACCGTGATGACTGGGAGAG3′ |
|  | R | 5′TAGCCAGTTTGTGCAGTTCC3′ |
| *Dmt1* | F | 5′TACAGTGAAGCCCAGCCAGA3′ |
|  | R | 5′ATGATCACAGCTCCCACGAT3′ |
| *Fpn*/*Slc40a1* | F | 5′TGGAAGCTAAGGGTGAGGAC3′ |
|  | R | 5′GGCCCAAGTCAGTGAAGGTA3′ |
| *Cp* | F | 5′TCTCGATGTGTGCAGCAGA3′ |
|  | R | 5′GCATCAATTAGGGTGGCAGG3′ |
| *Ncoa4* | F | 5′AGCAGAAGTCAGCATCCAGT3′ |
|  | R | 5′AGTCCTGTGGGTTGGTACTG3′ |
| *Acsl3* | F | 5′CCACGTATCTTCAACACCATCA3′ |
|  | R | 5′TGTATGCAGAGTCCGGTTTG3′ |
| *Acsl4* | F | 5′AGCGTTCCTCCAAGTAGACC3′ |
|  | R | 5′GTCCTTCGGTCCTAGTCCAG3′ |
| *Lpcat3* | F | 5′GCGGCTCATCTTCTCCATCT3′ |
|  | R | 5′GAGTGGTAGAACTGGTGGCC3′ |
| *Alox15* | F | 5′TGCTGTTTGTGAGAGTGCAG3′ |
|  | R | 5′TACTCCGATCCCTGGTCTCC3′ |
| *Aifm2* | F | 5′GGAGTACATCAAGGTGGAGAC3′ |
|  | R | 5′CTCAAATGCACTGCGGTAGG3′ |
| *Gch-1* | F | 5′TGTTCTCCATGTGTGAGCATC3′ |
|  | R | 5′CAATCTGTTTGGTGAGGCGC3′ |
| *Gpx4* | F | 5′AACAGCTCCGAGTTCCTGG3′ |
|  | R | 5′CACACGAAACCCCTGTACTT3′ |
| *Slc7a11* | F | 5′ATCATCACAGTGGGCTACGT3′ |
|  | R | 5′GAGAATTTTCCCAGCAGCCG3′ |
| *Nfe2l2* | F | 5′GCAGGACATGGATTTGATTGAC3′ |
|  | R | 5′CGGCTGAATTGGGAGGAATT3′ |
| *Sqstm1* | F | 5′ACCCACTACCCCAGAAAGTT3′ |
|  | R | 5′CACCGACTCCAAGGCTATCT3′ |
| *Vdac2* | F | 5′AACCTCGGCTGTGATGTTGA3′ |
|  | R | 5′GGAAGTCCCCAGTCCTGTAG3′ |
| *Irp1*/*Aco1* | F | 5′TCAAGATACGGACGCTTACCA3′ |
|  | R | 5′GTATGACTCGGGCTGGCTTA3′ |
| *Irp2*/*Ireb2* | F | 5′CTGGAACTGGAATGGCTCATC |
|  | R | 5′CCAACTCCCCACCCAAGAAT3′ |
| *Fbxl5* | F | 5′TGACTTCCGTGCTCTTCTCC3′ |
|  | R | 5′ACTGTTCGTACTCATTCTTGACA3′ |
| *Hamp1* | F | 5′GCGATACCAATGCAGAAGAGA3′ |
|  | R | 5′AATAATGGGGTTGAGGGGCT3′ |
| *Atf2* | F | 5′CCTCCGGGGCTAGTTTGTAA3′ |
|  | R | 5′CACTTTGTAGCTGGTGGGC3′ |
| *Gapdh* | F | 5′ATTGTTGCCATCAACGACC3′ |
|  | R | 5′CATGGACTGTGGTCATGAGC3′ |

F: forward primer. R: reverse primer.
