# Supplementary material for: Restoring Iron Homeostasis via Smoothened Inhibition: A Novel Strategy Against Hearing Loss
Source: Adv Sci (Weinh). 2026 Apr 17;13(29):e20749. doi: 10.1002/advs.202520749 (PMC13205593; doi:10.1002/advs.202520749)
Supplement: Supplementary file 2 — Supporting File 2: advs74603‐sup‐0002‐DataSet.docx. [file ADVS-13-e20749-s002.docx]

**Western Blot Images**

1. **Figure7G-Repeat1**

**Whole Gel**

**
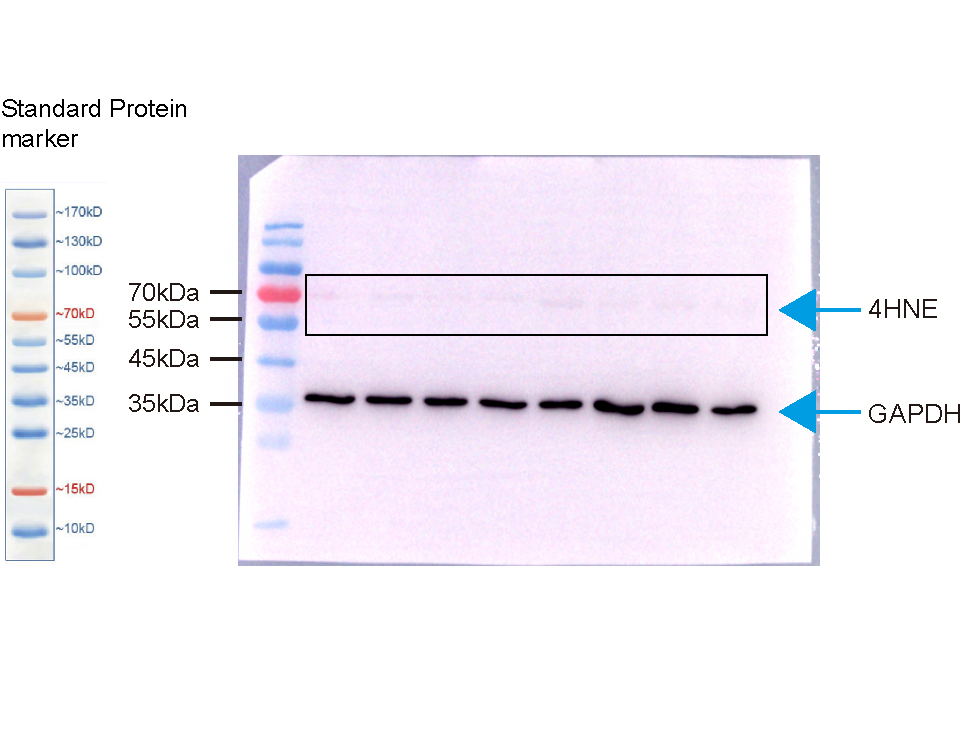
**

**4HNE**

**
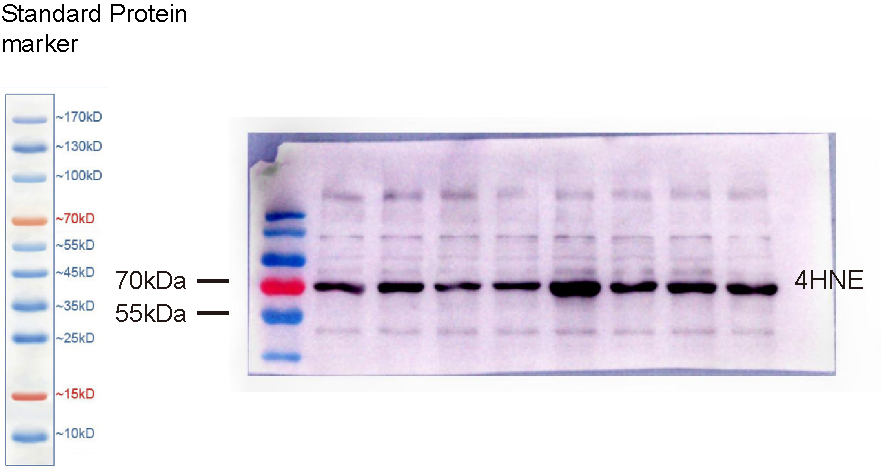
**

1. **Figure7G-Repeat2**

**Whole Gel**

**
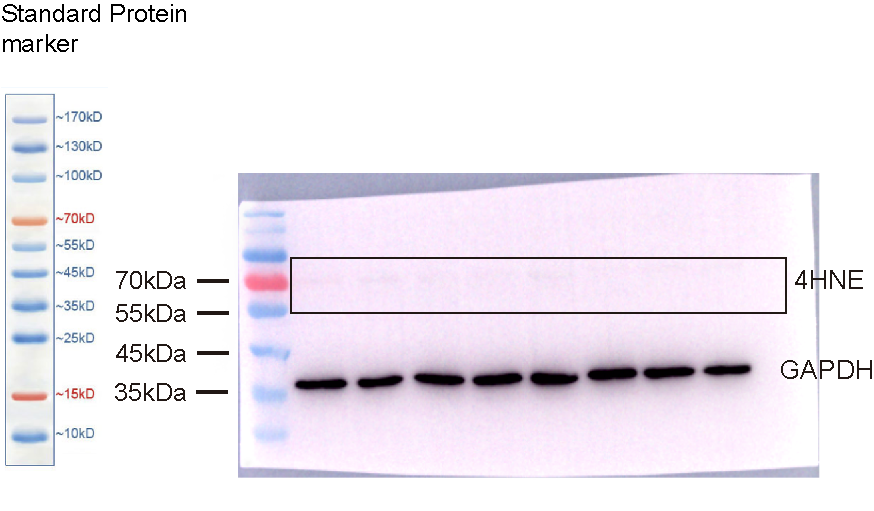
**

**4HNE**

**
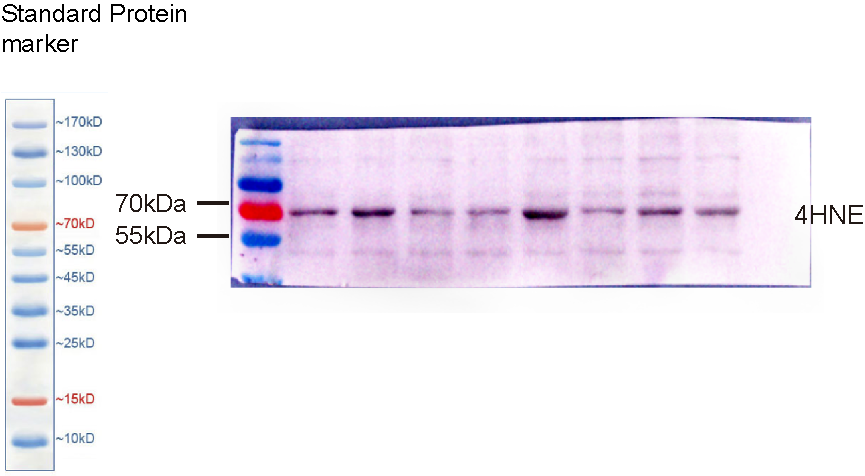
**

1. **Figure7G-Repeat3**

**Whole Gel**

**
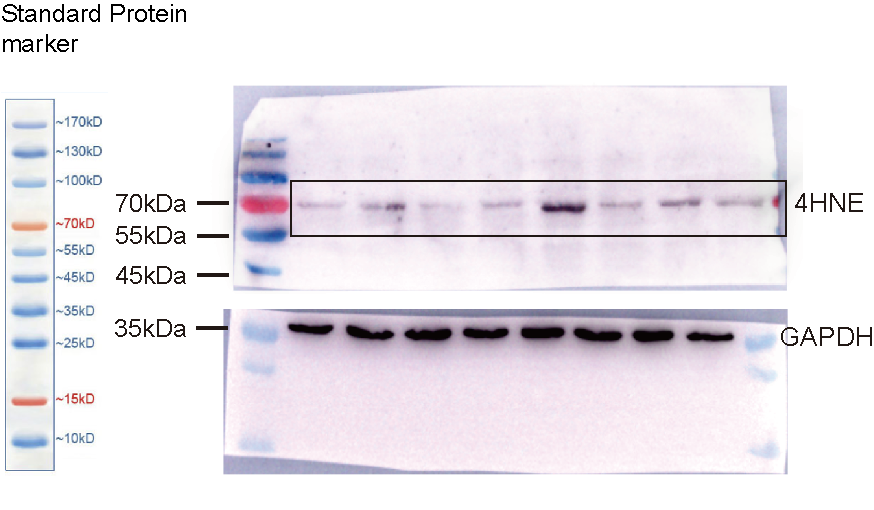
**

**4HNE**

**
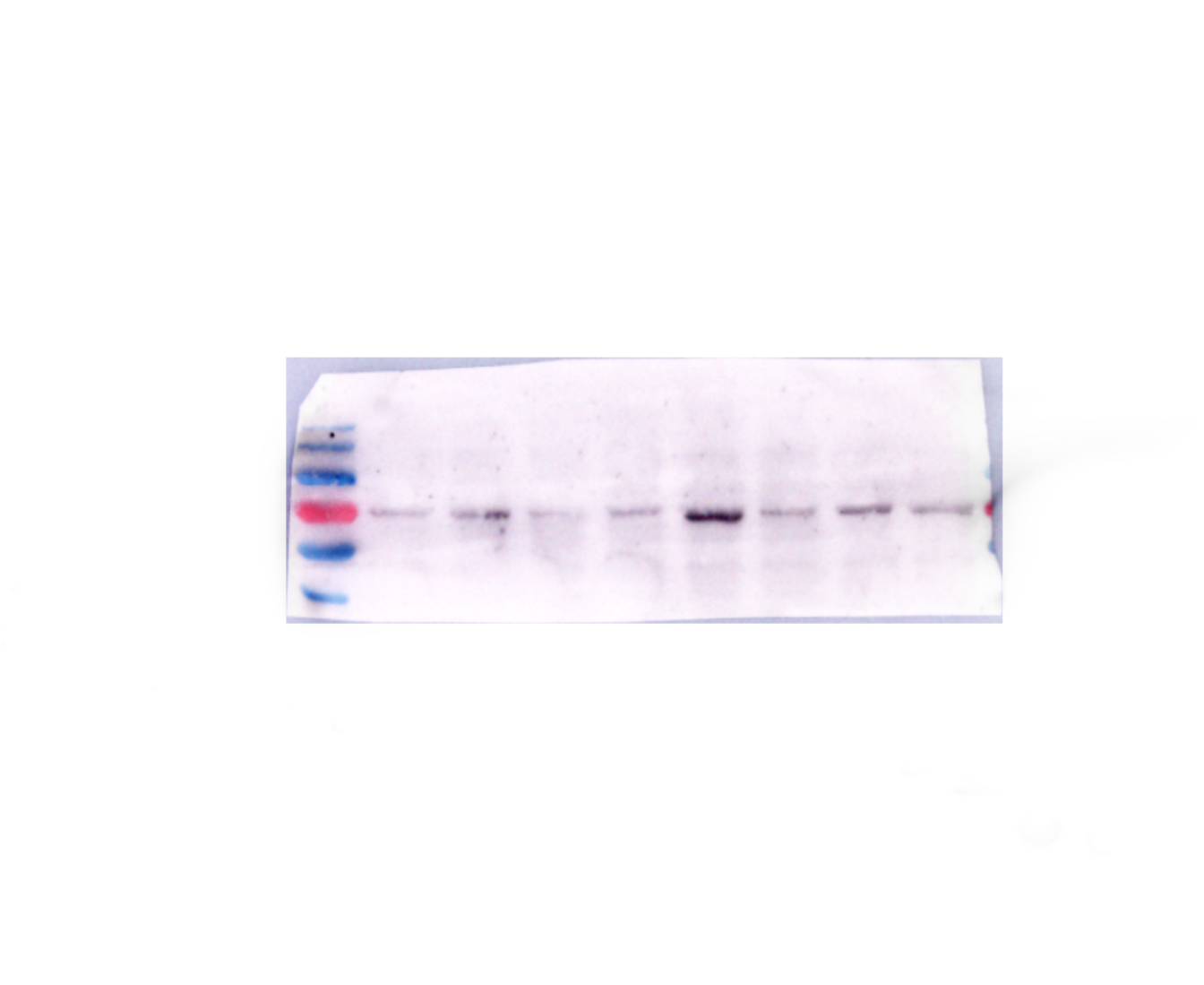
**

**GAPDH**

**
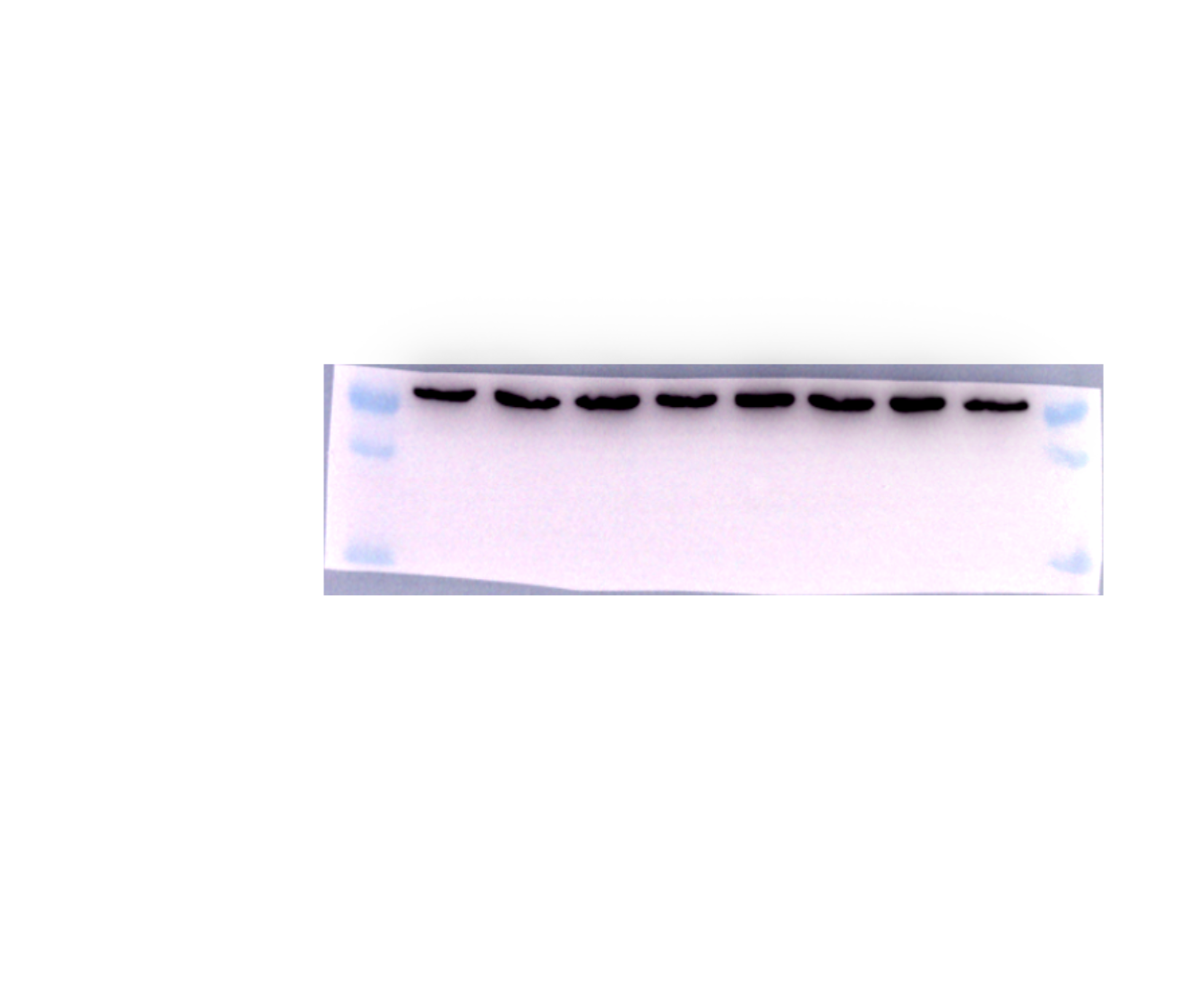
**

1. **Figure8A**

**Whole Gel**


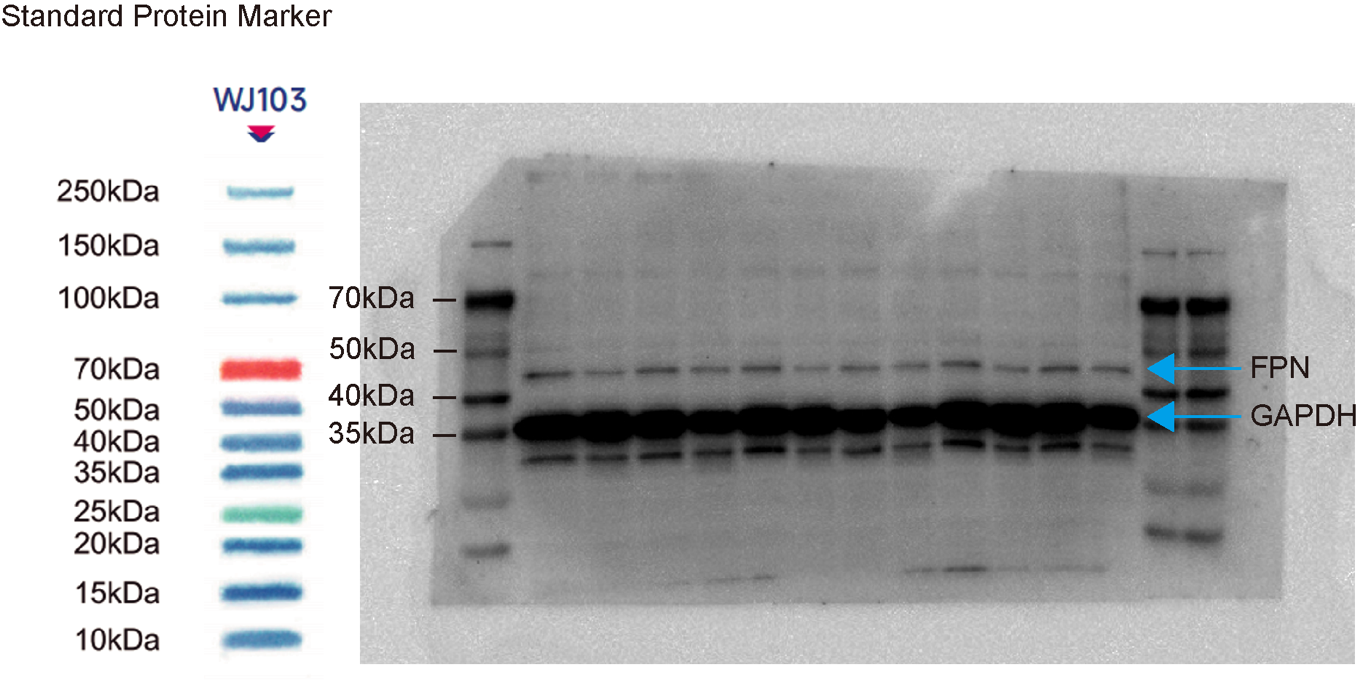


**FPN**

**
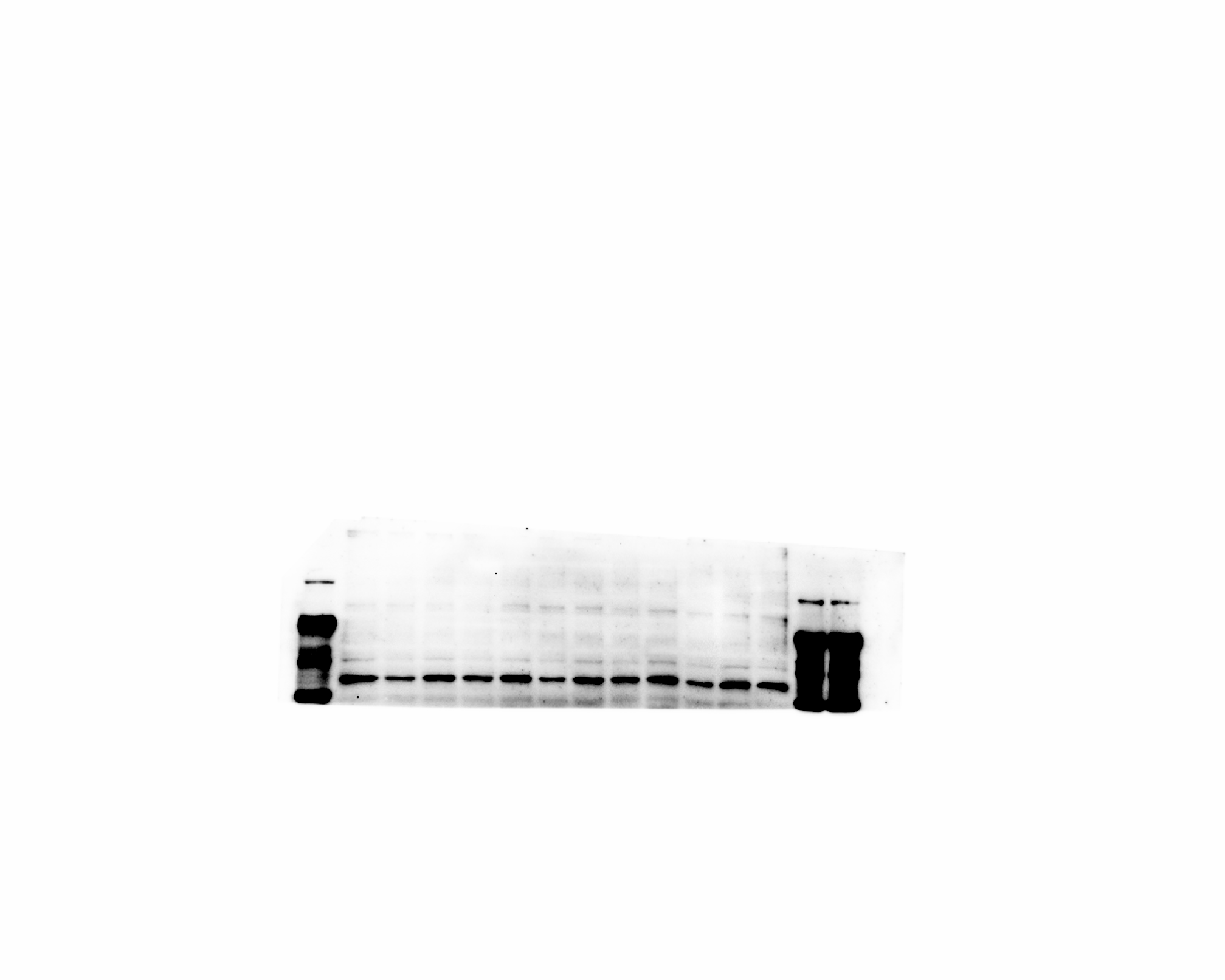
**

**GAPDH**

**
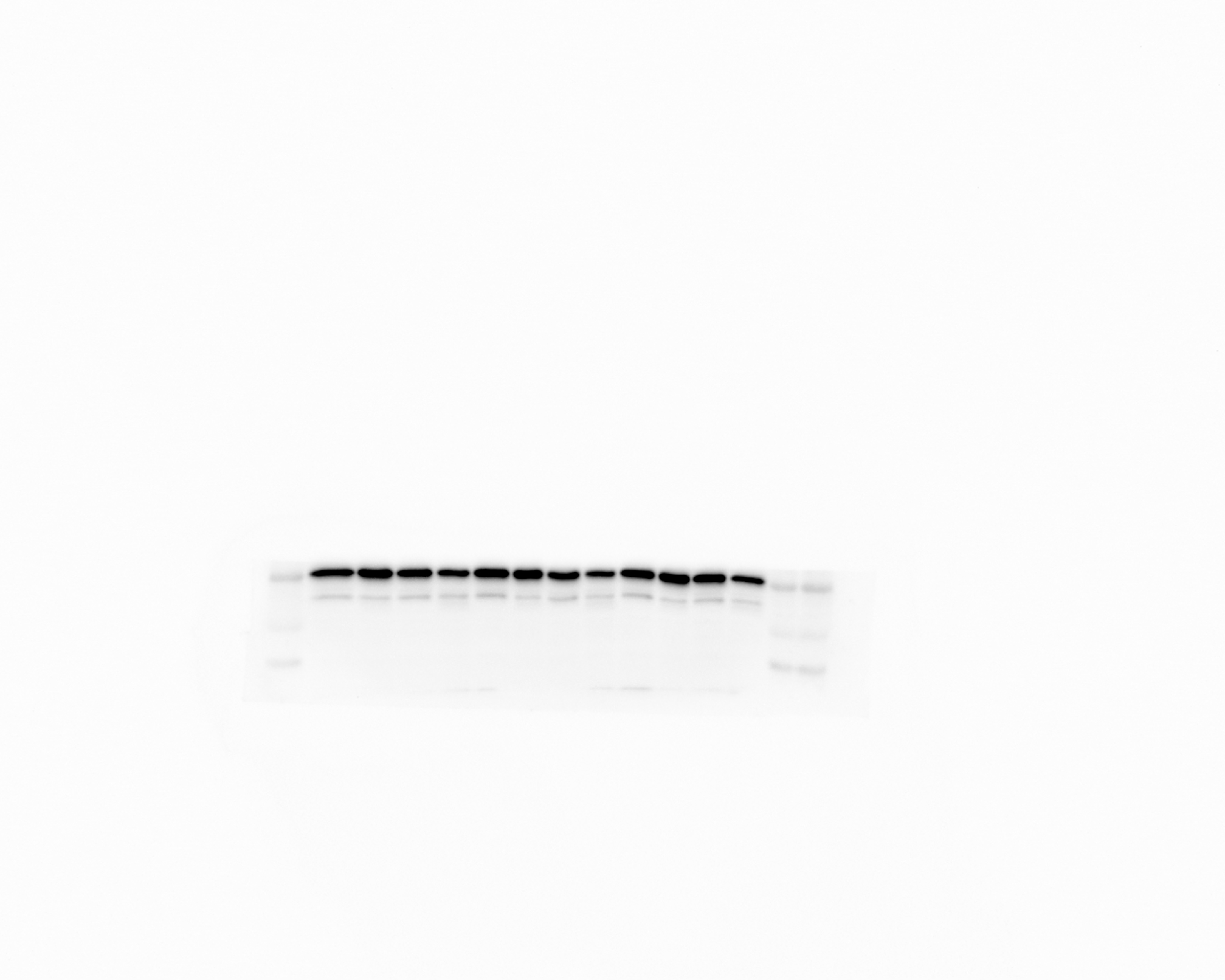
**

1. **Figure8D**

**Whole Gel**

**
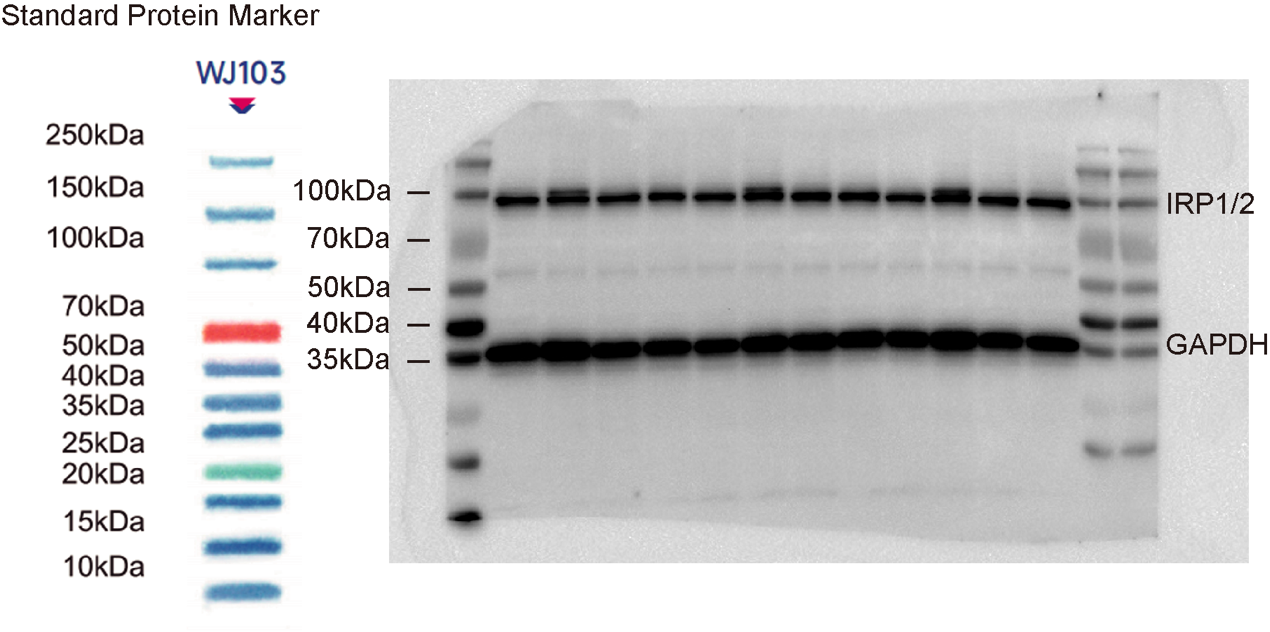
**

1. **Figure8G**

**Whole Gel**

**
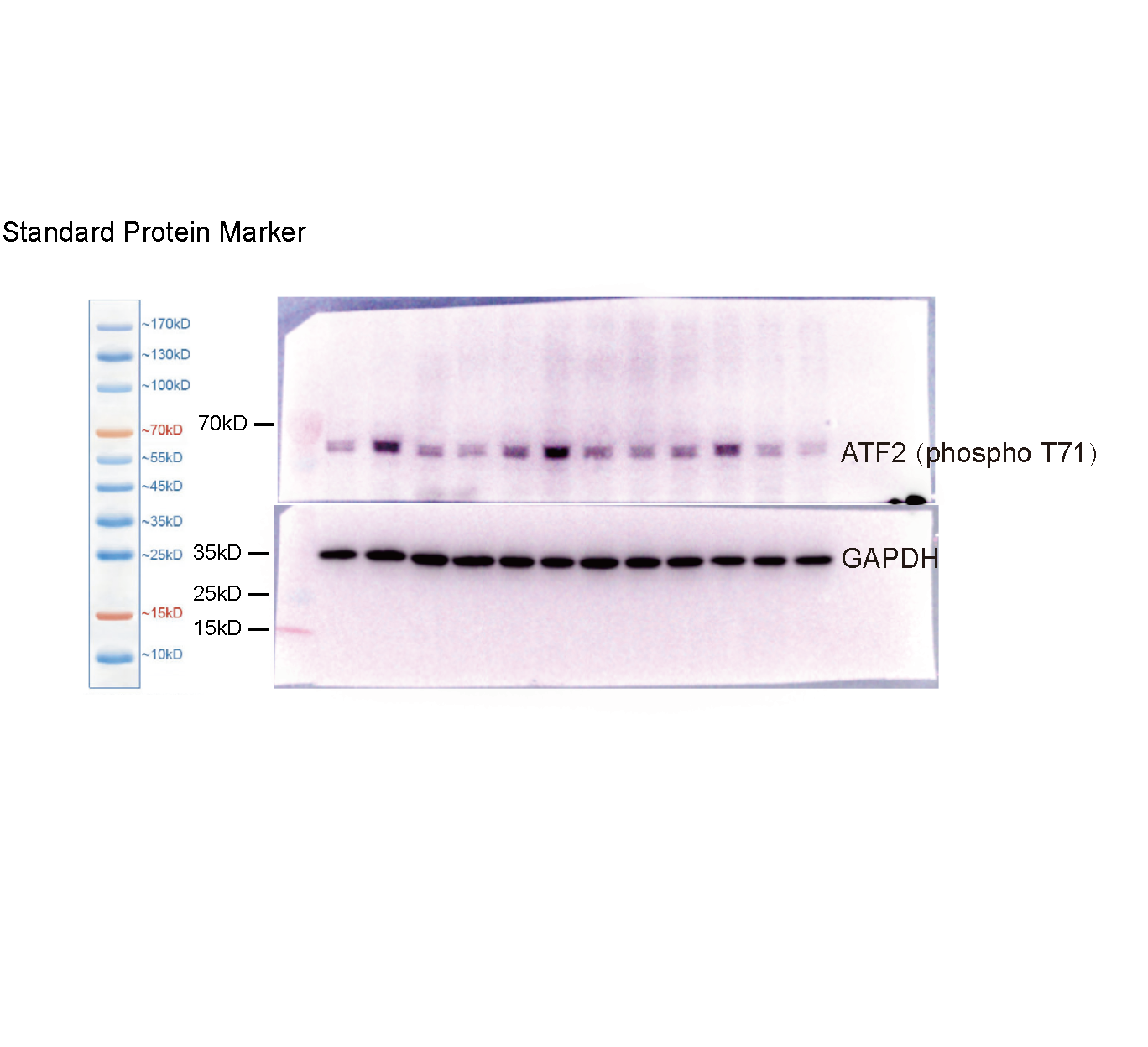
**

**ATF2(pT71)**

**
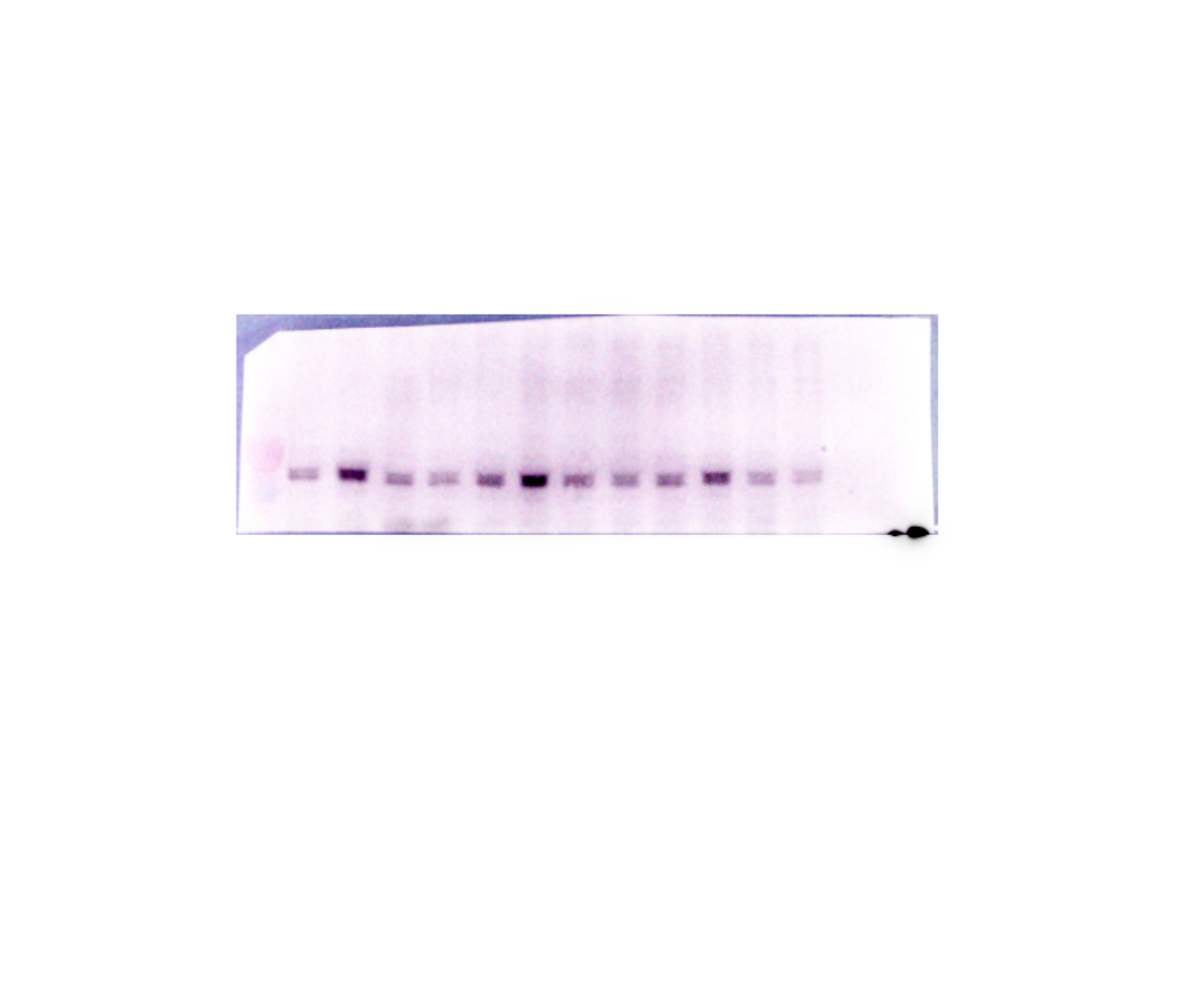
**

**GAPDH**

**
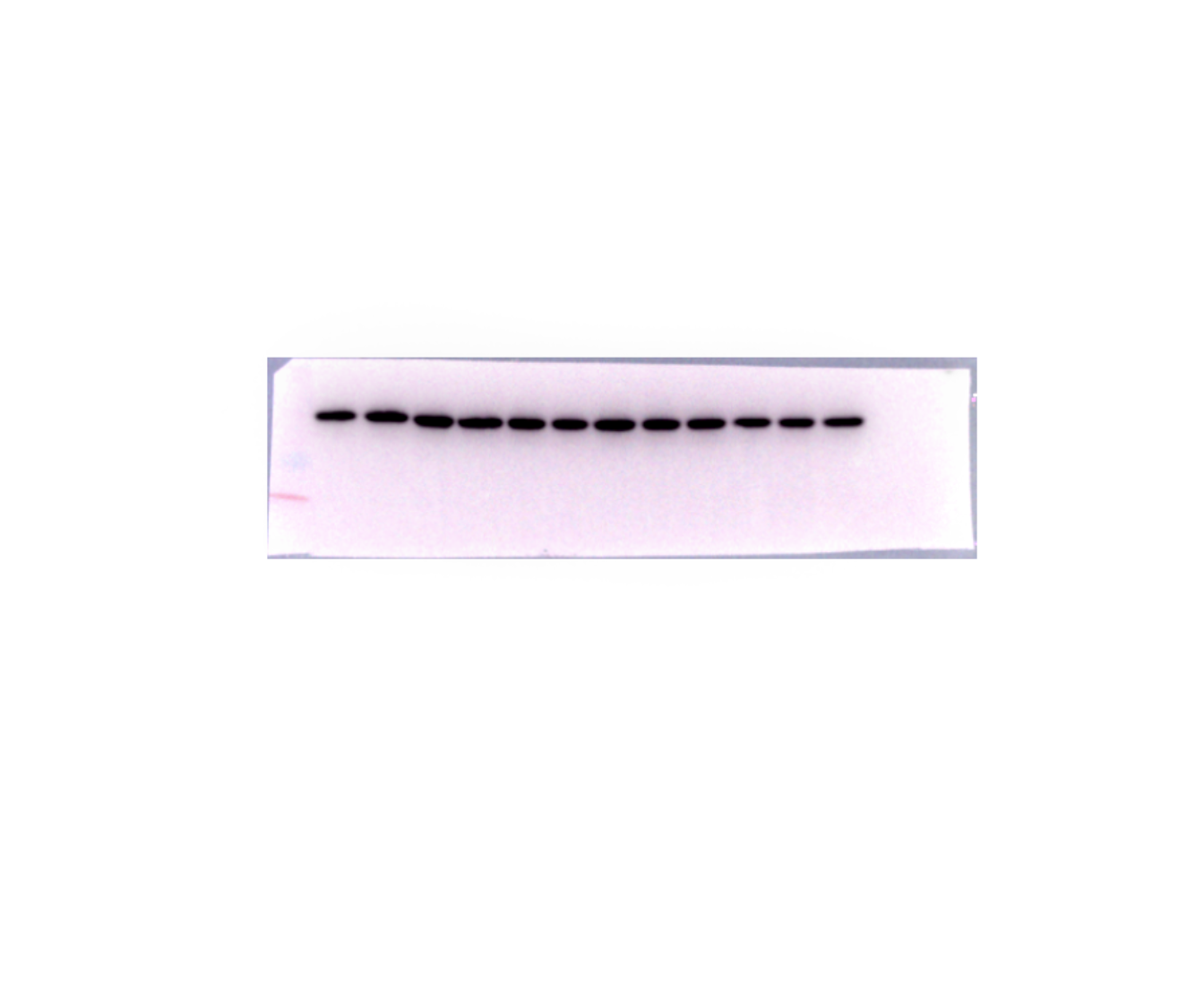
**

1. **Figure8K**

**Whole Gel**

**
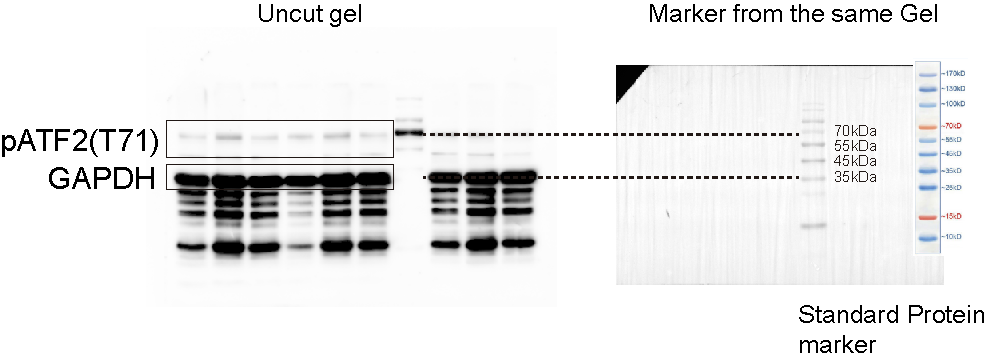
**

**ATF2(pT71)**

**
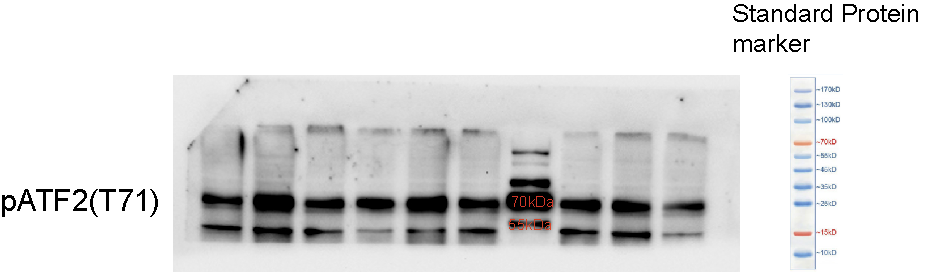
**

1. **Figure8Q-Repeat1**

**Whole Gel**

**
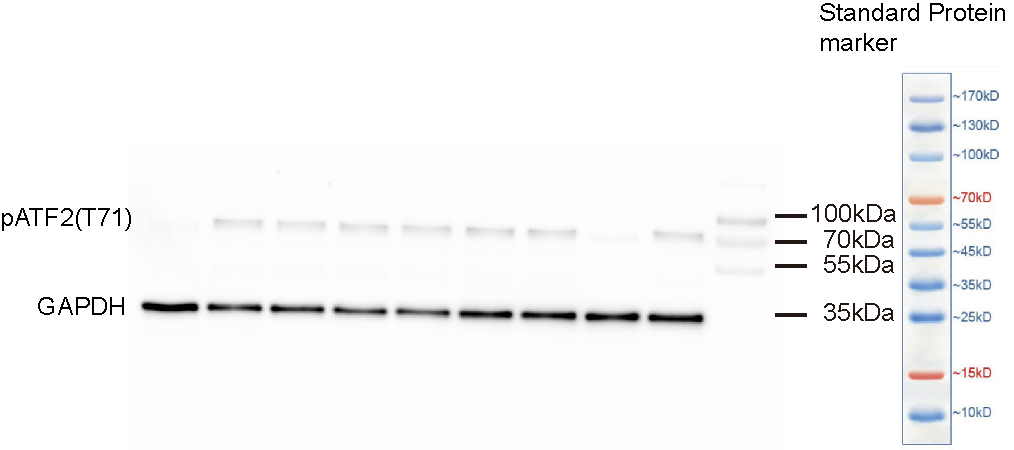
**

**ATF2(pT71)**

**
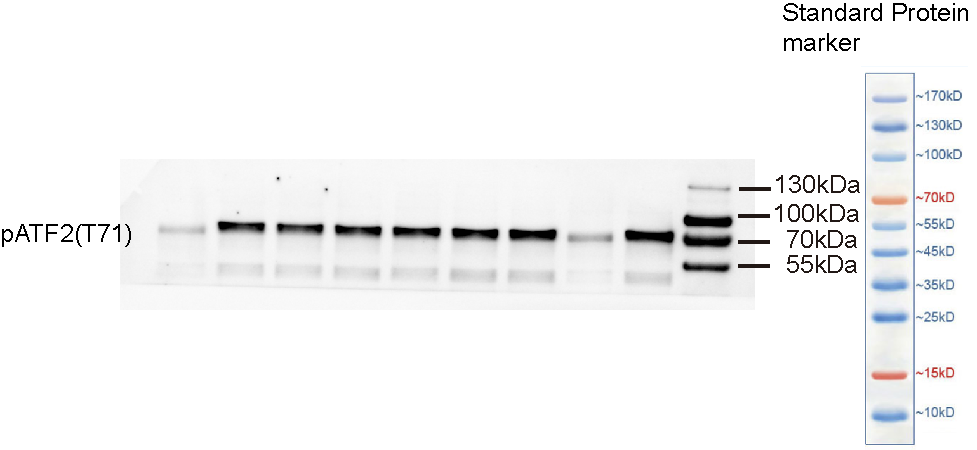
**

1. **Figure8Q-Repeat2**

**Whole Gel**

**
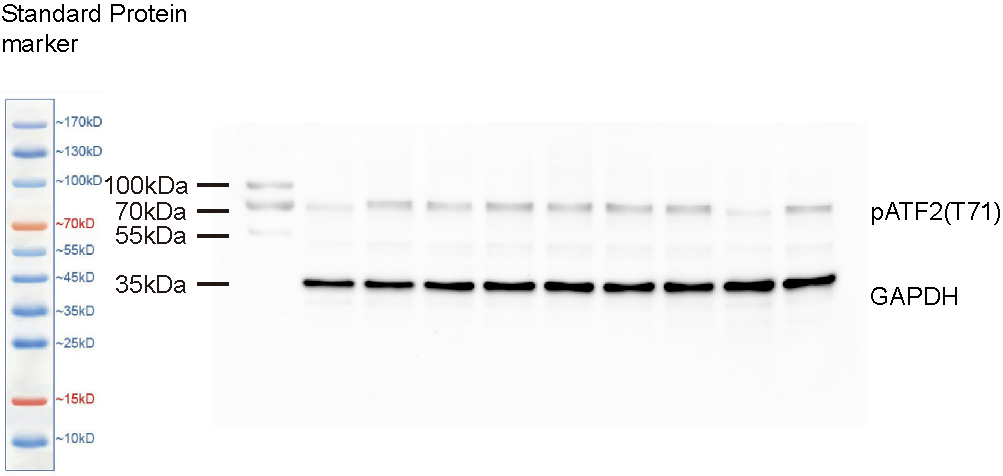
**

**ATF2(pT71)**

**
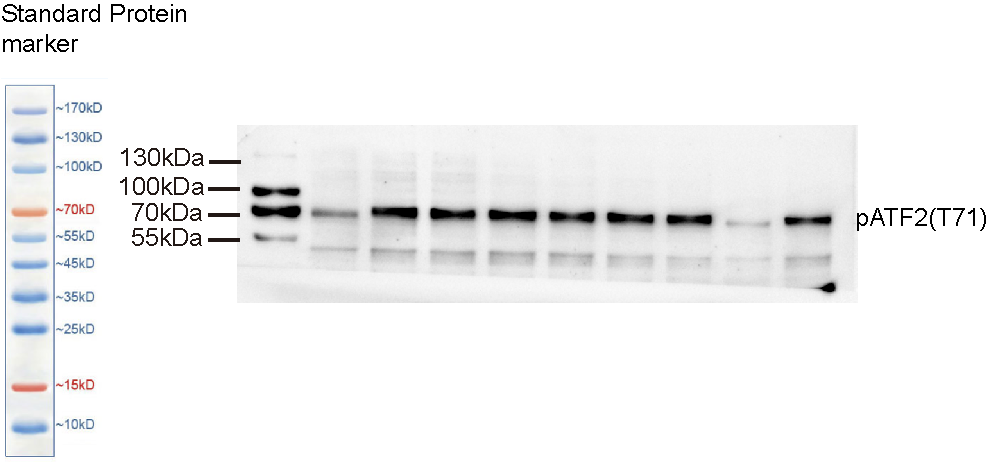
**

1. **Figure8Q-Repeat3**

**Whole Gel**

**
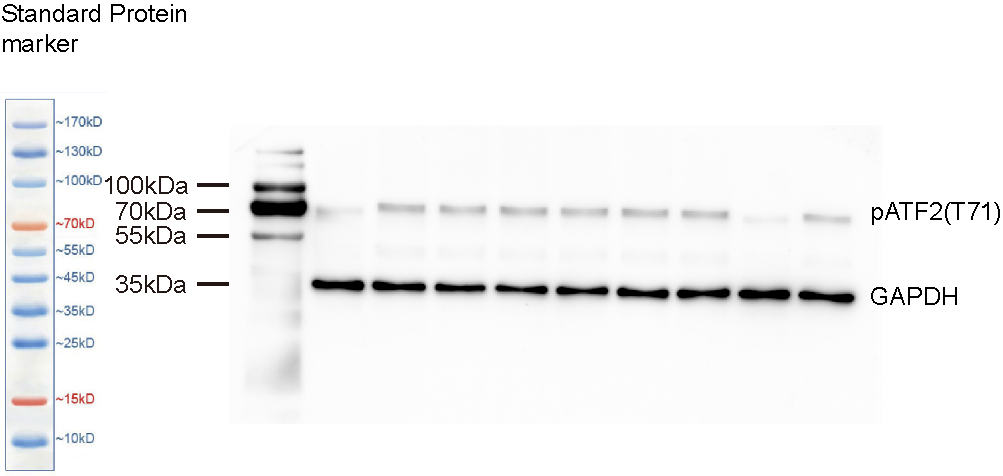
**

**ATF2(pT71)**

**
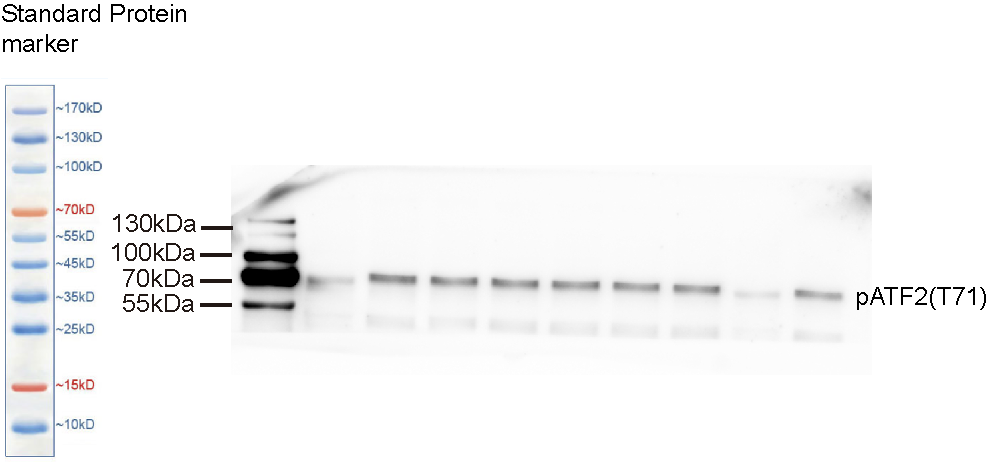
**

**Microscope Images**

**Figure 2B**

**
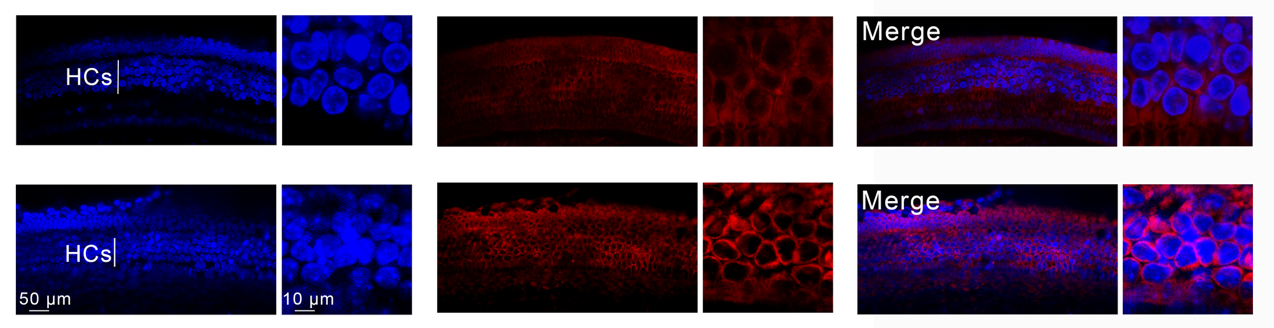
**

**Figure 2D**

**
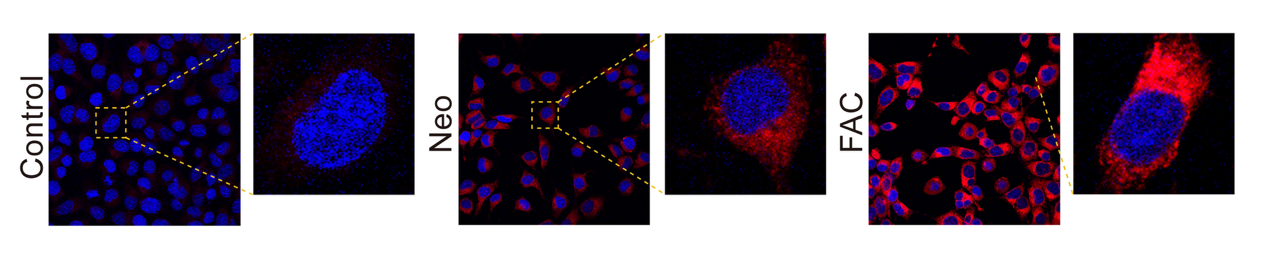
**

**Figure 2G**

**
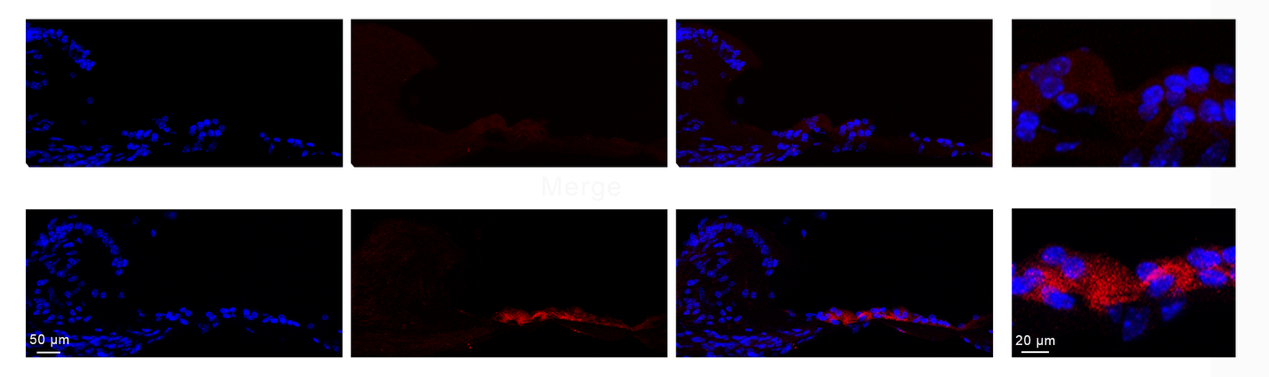
**

**Figure 3F and 3G**

**
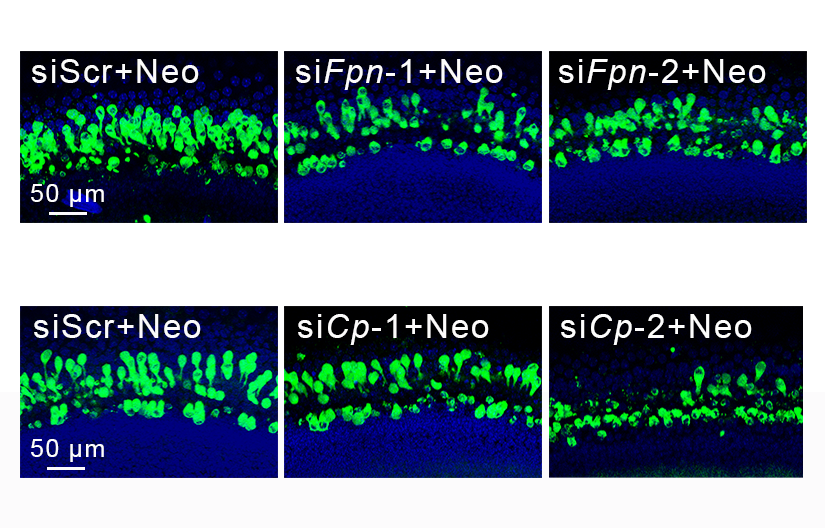
**

**Figure 4D**

**
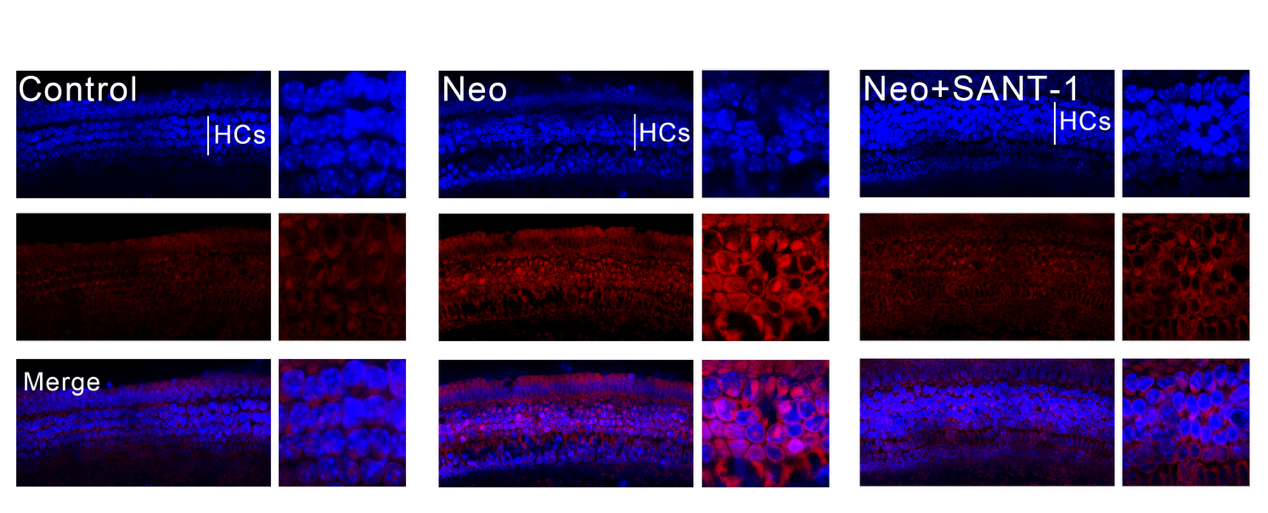
**

**Figure 4H**

**
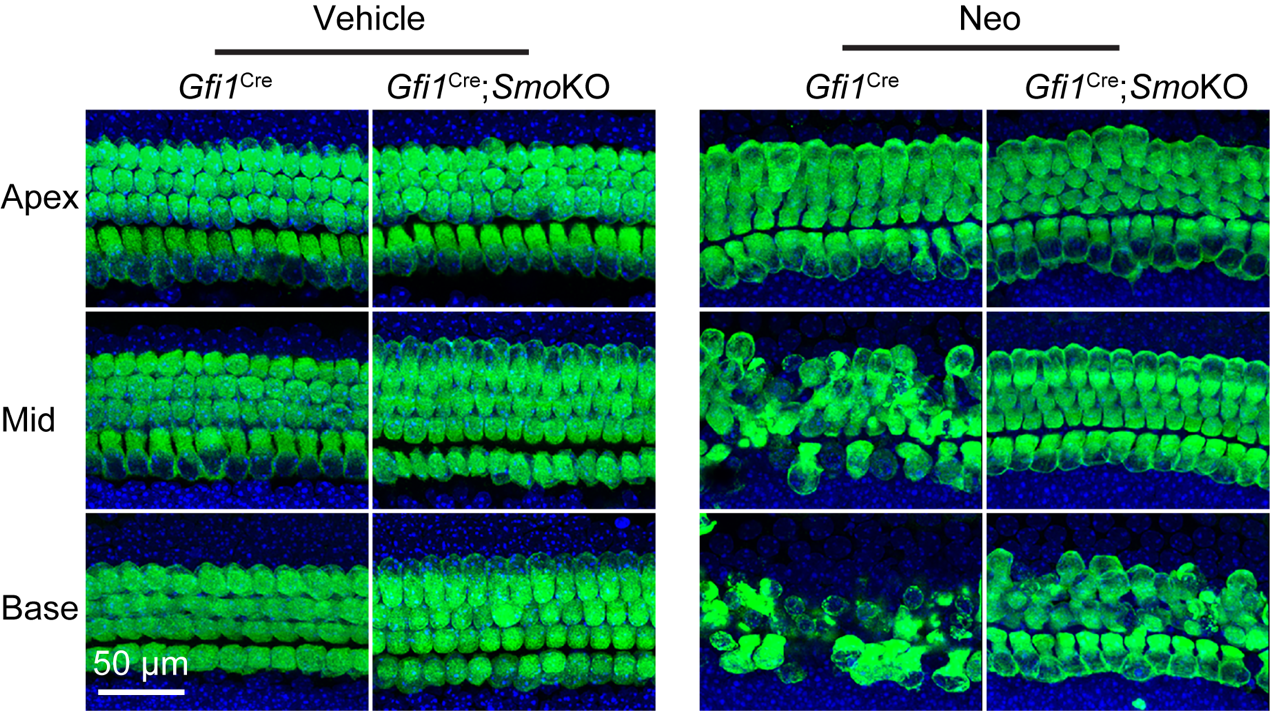
**

**Figure 5E**

**
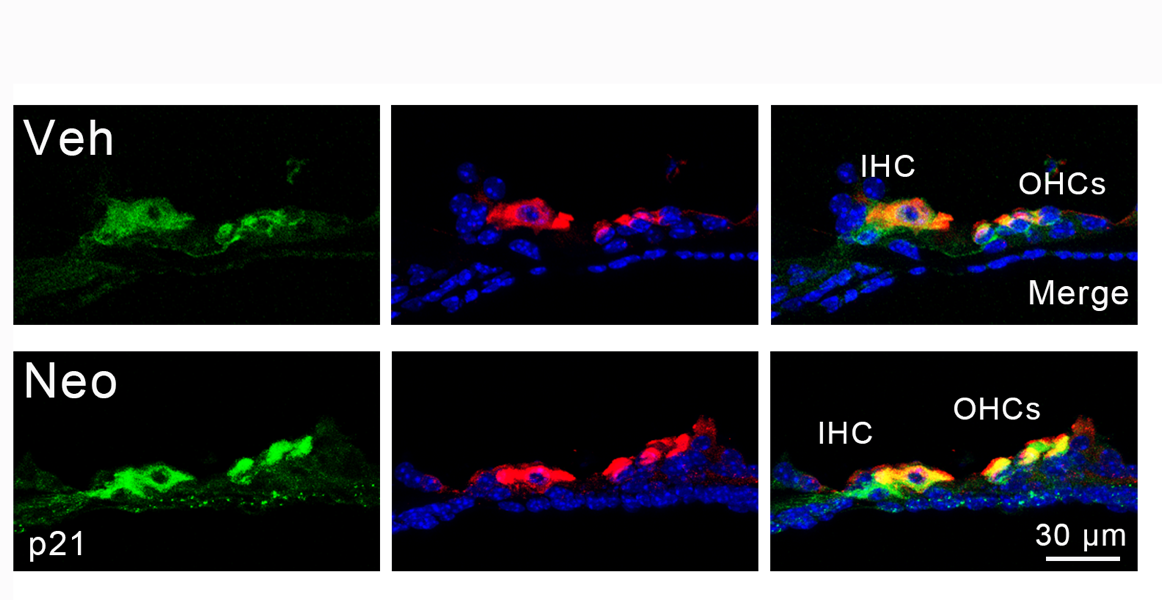
**

**Figure 5G**

**
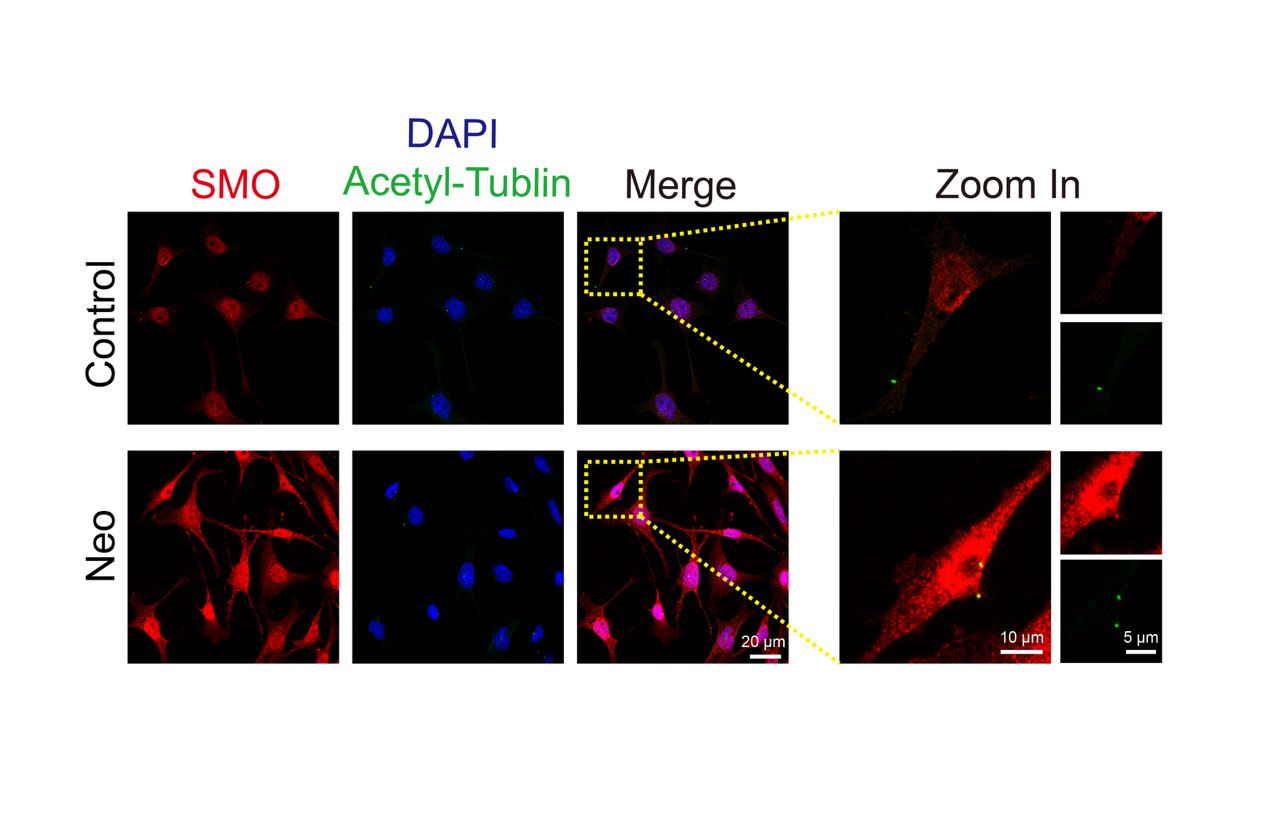
**

**Figure 5J**

**
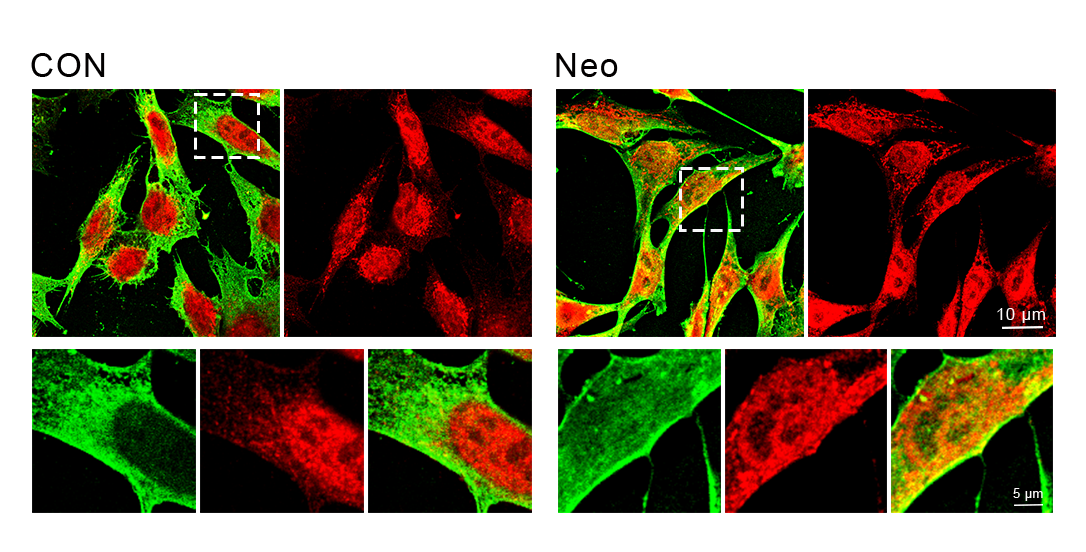
**

**Figure 6A**

**
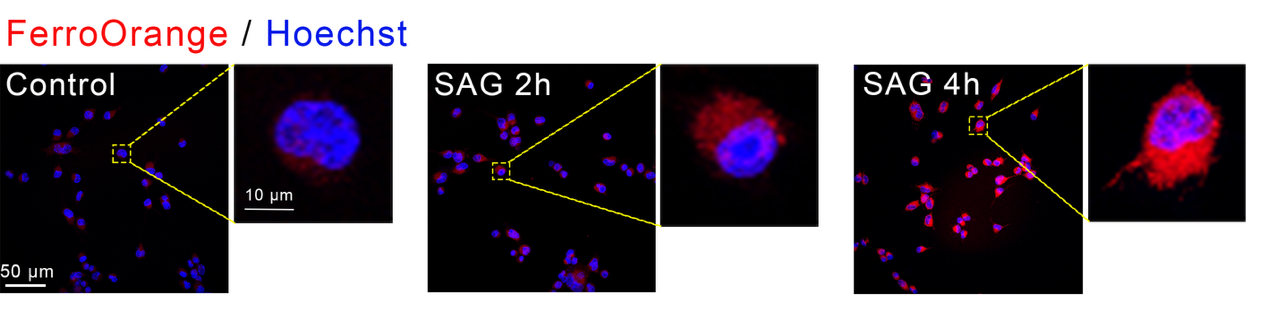
**

**Figure 6F**

**
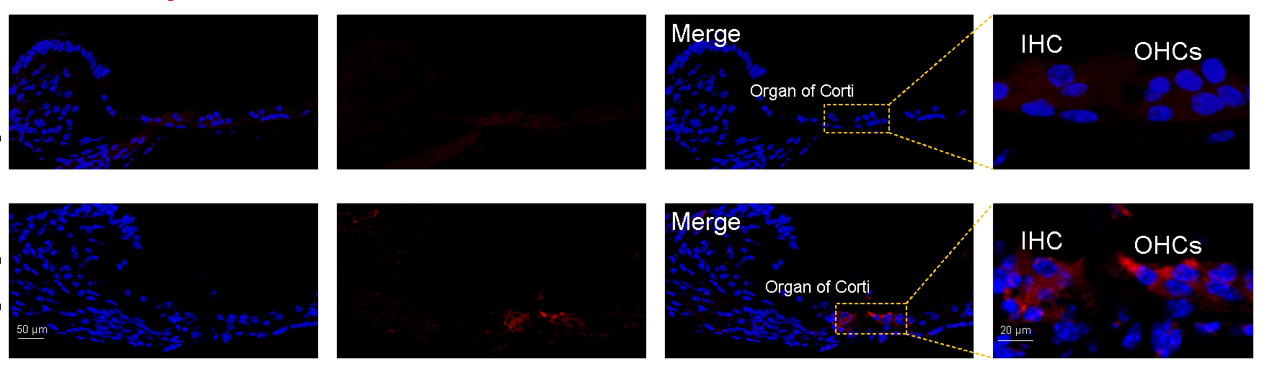
**

**Figure 6I**

**
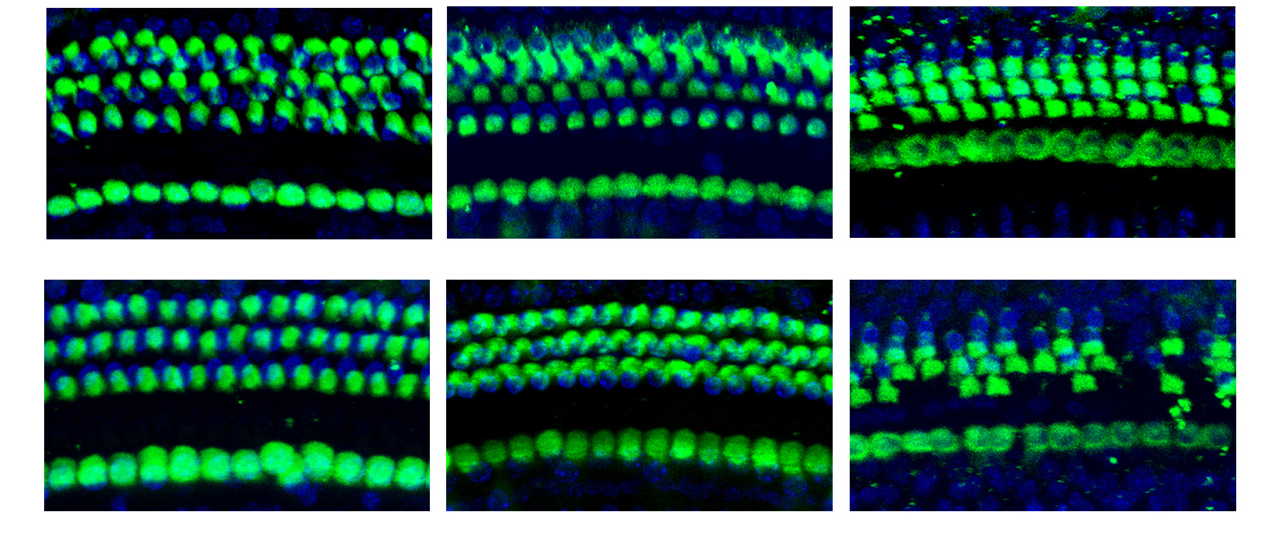
**

**Figure 7A**

**
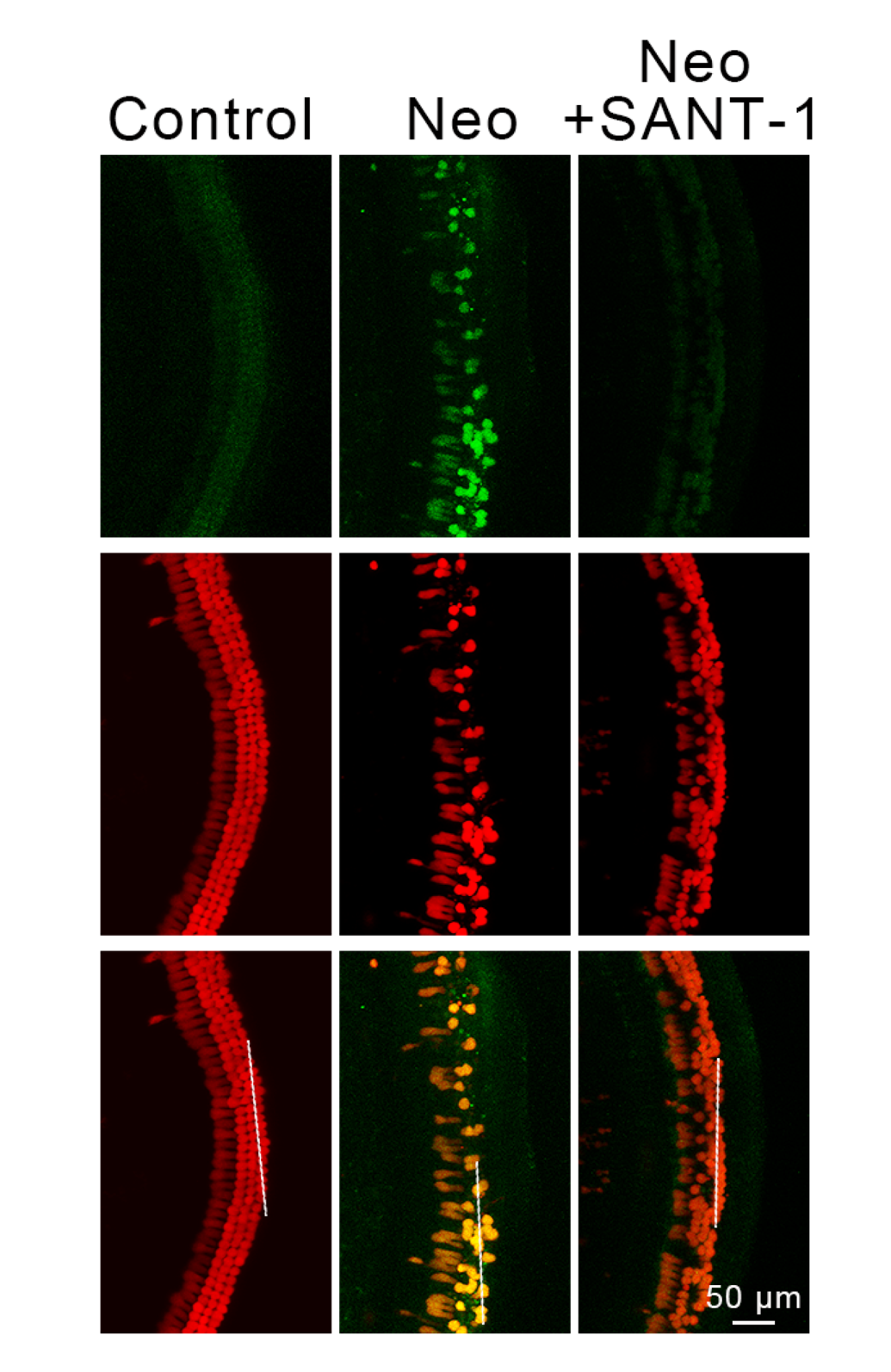
**

**Figure 7C**

**
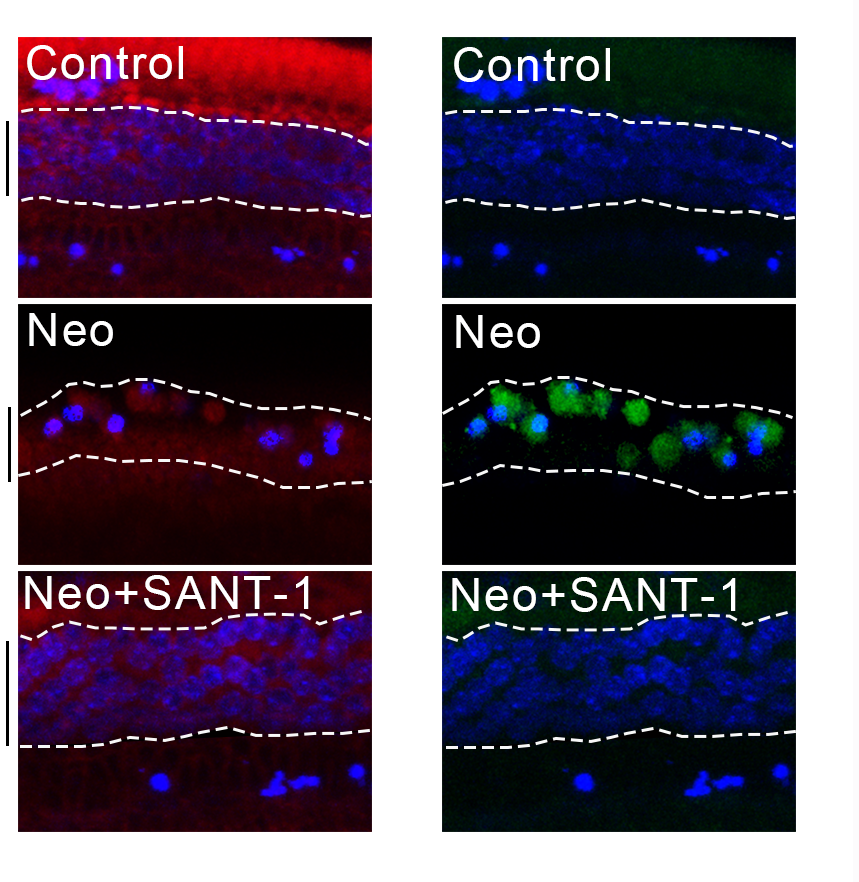
**

**Figure 7E**

**
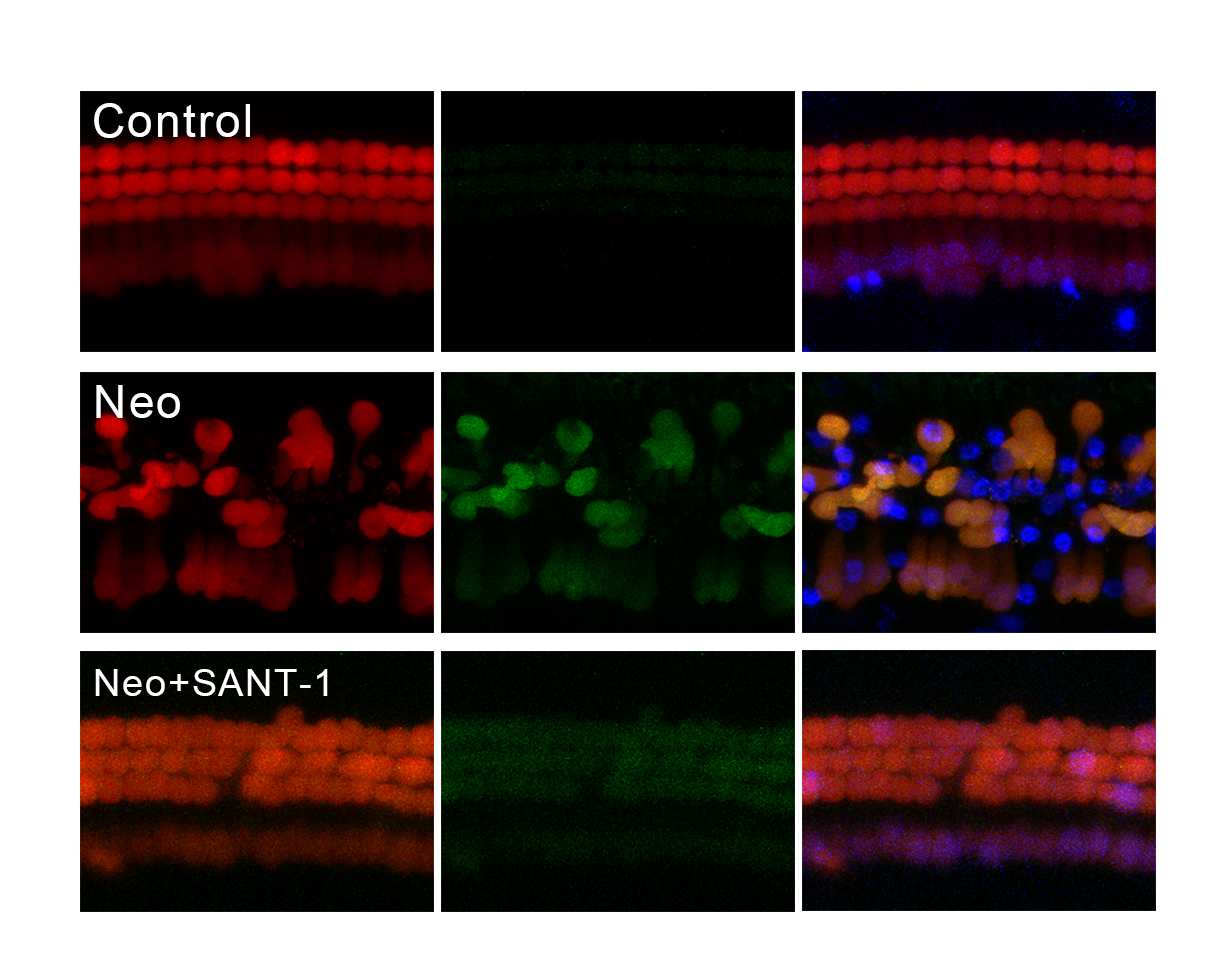
**

**Figure 7H**

**
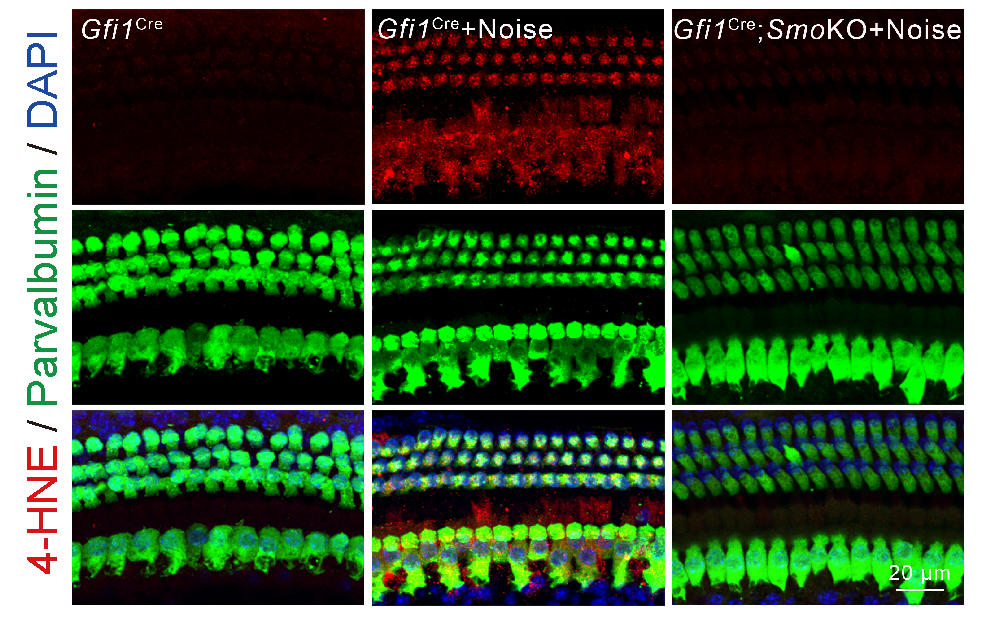
**

**Figure 9C**

**
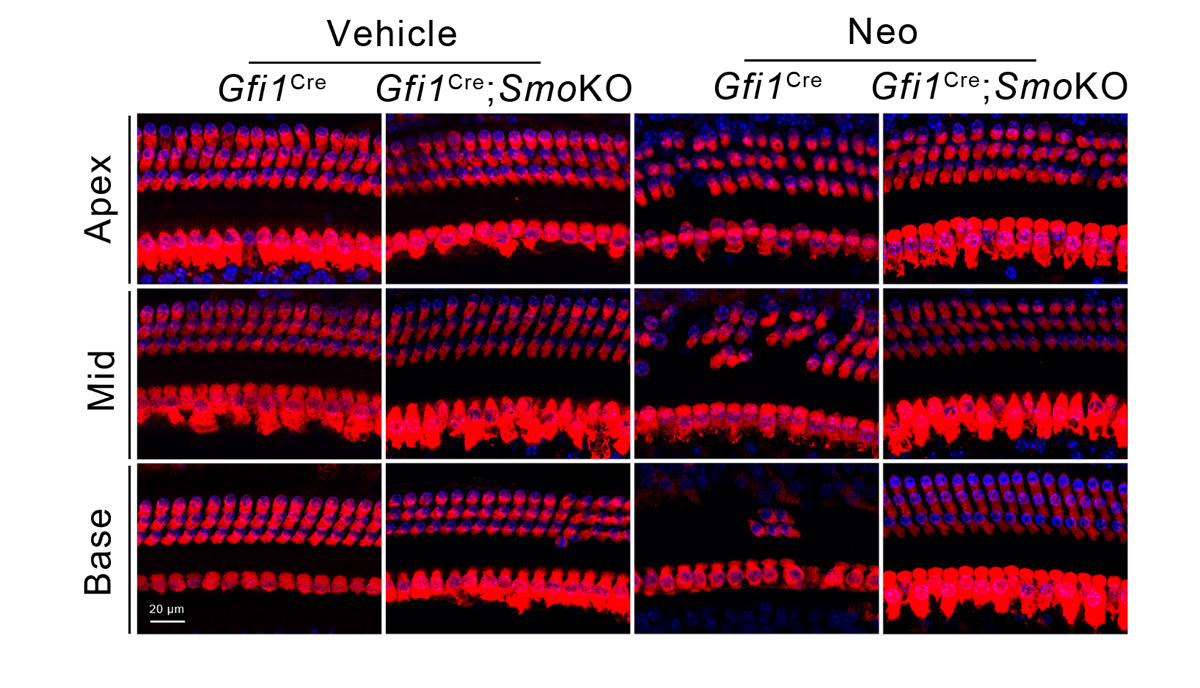
**

**Figure 9G**

**
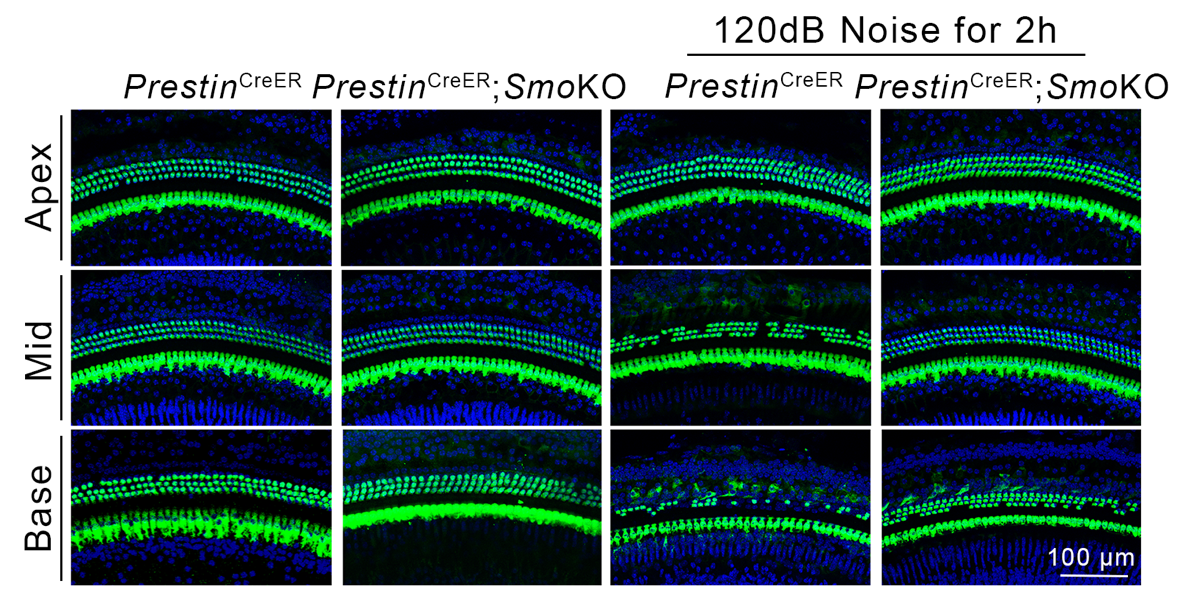
**

**Figure 10F**

**
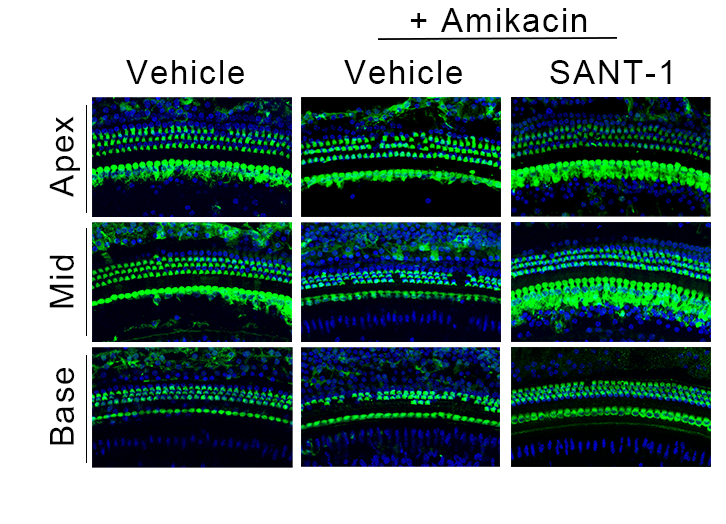
**

**Figure S1A**

**
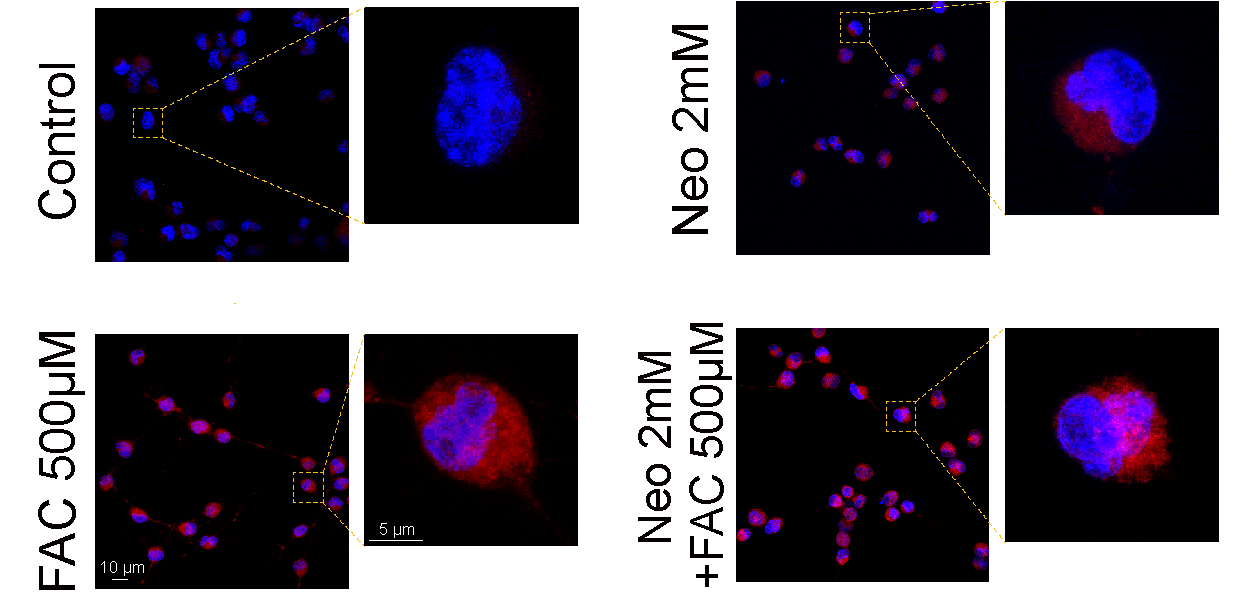
**

**Figure S1C**

**
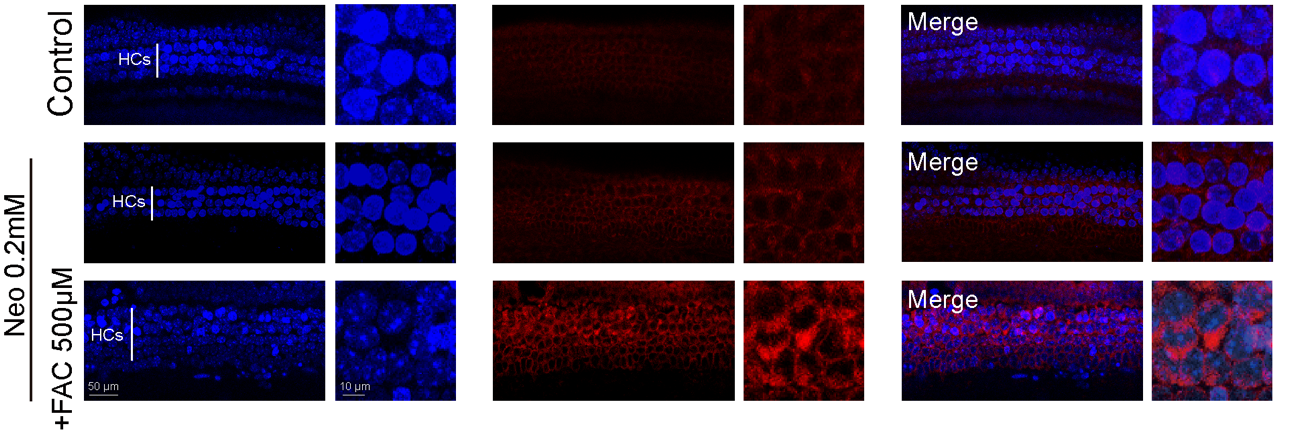
**

**Figure S2C**

**
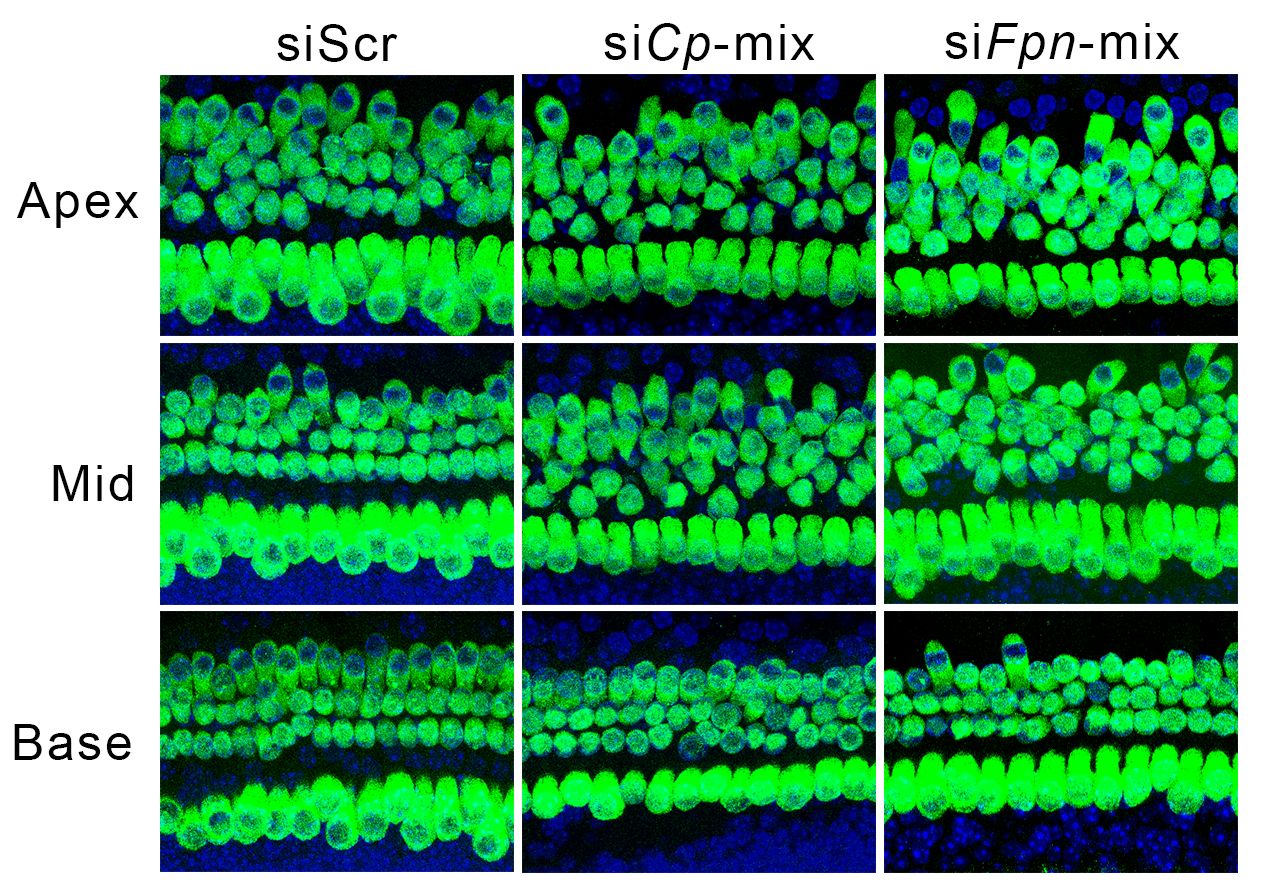
**

**Figure S3D**

**
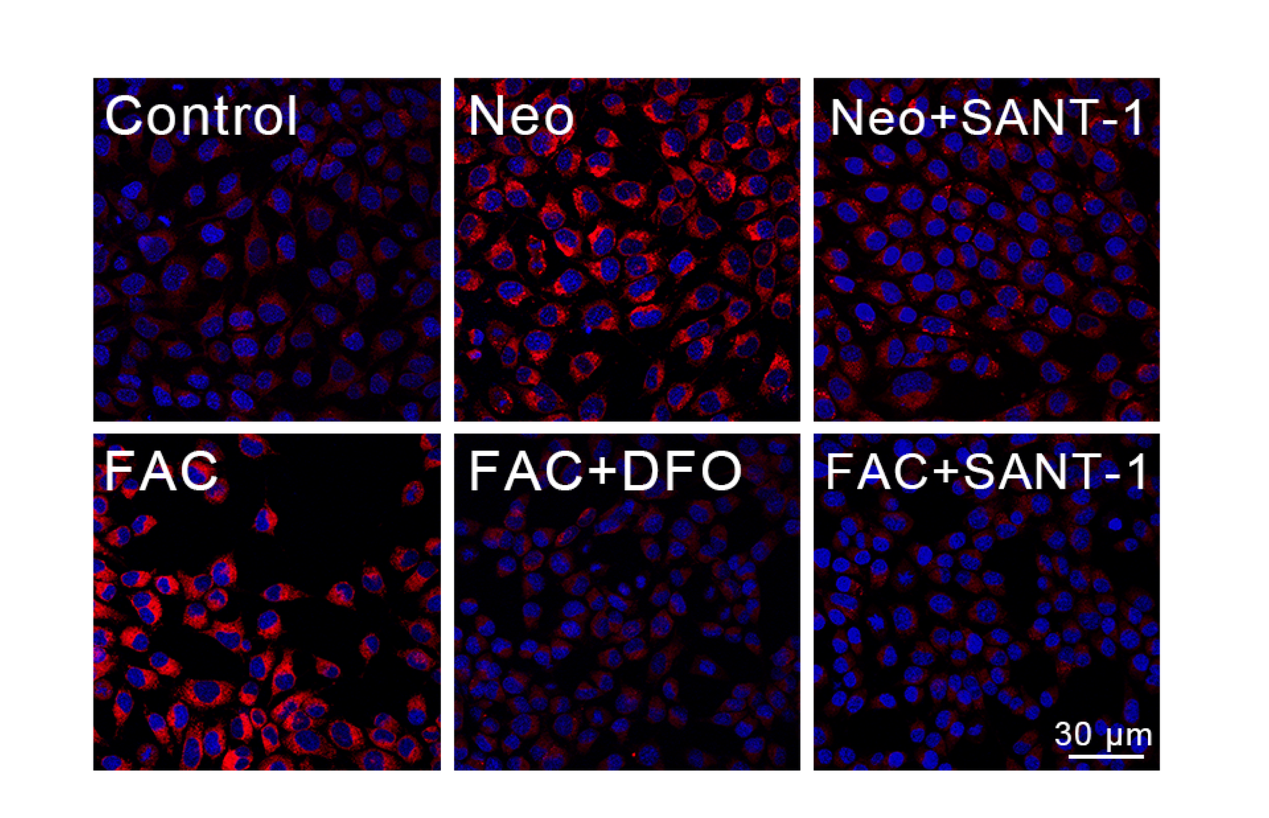
**

**Figure S3F**

**
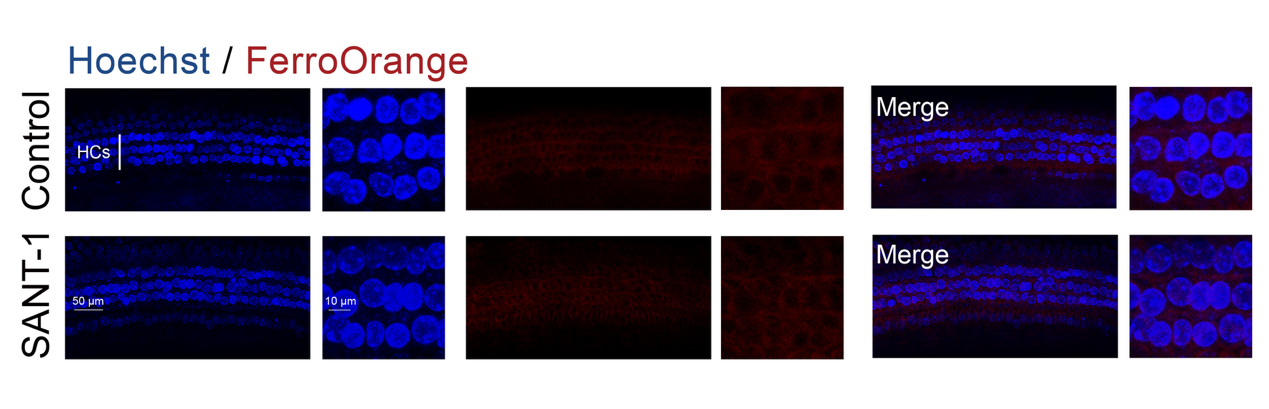
**

**Figure S5A**

**
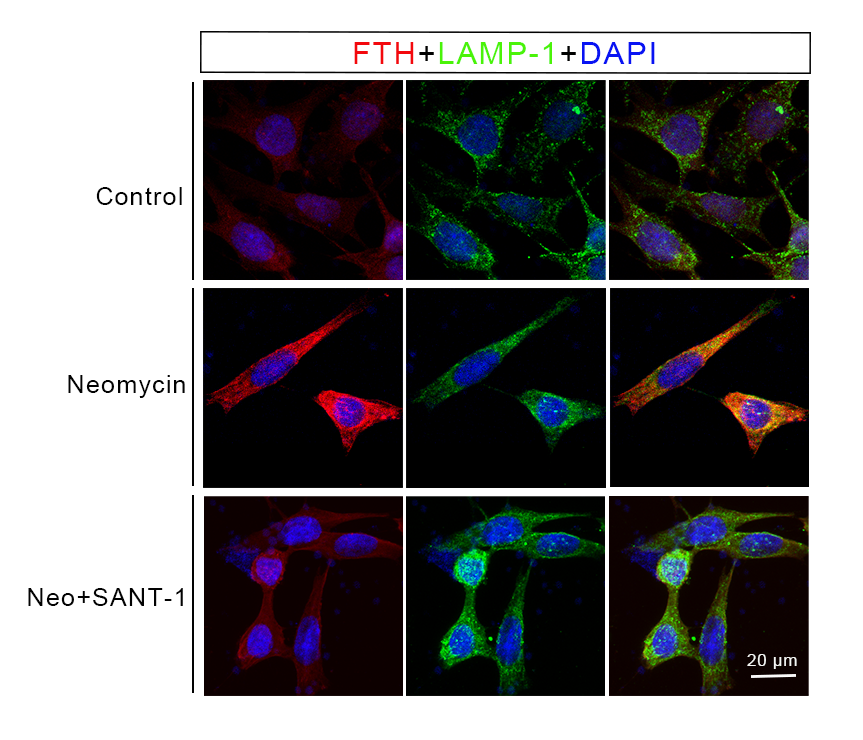
**

**Figure S6A**

**
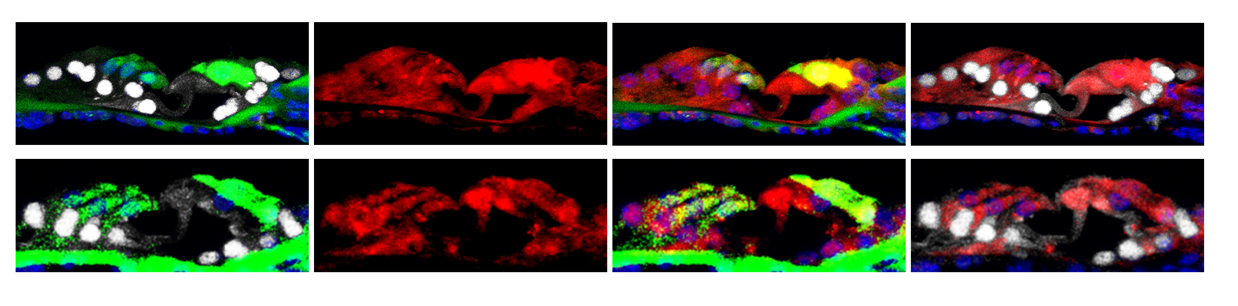
**

**Figure S6B**

**
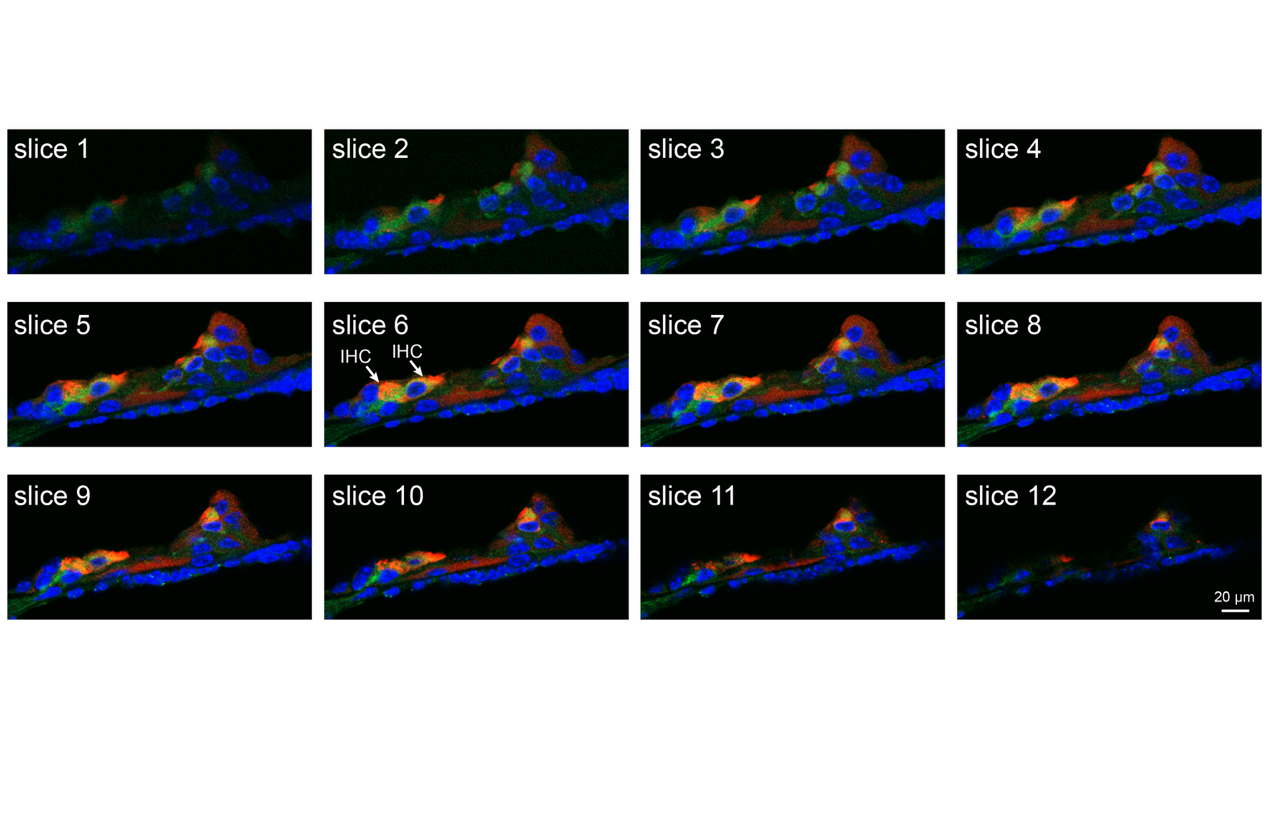
**

**Figure S6C**

**
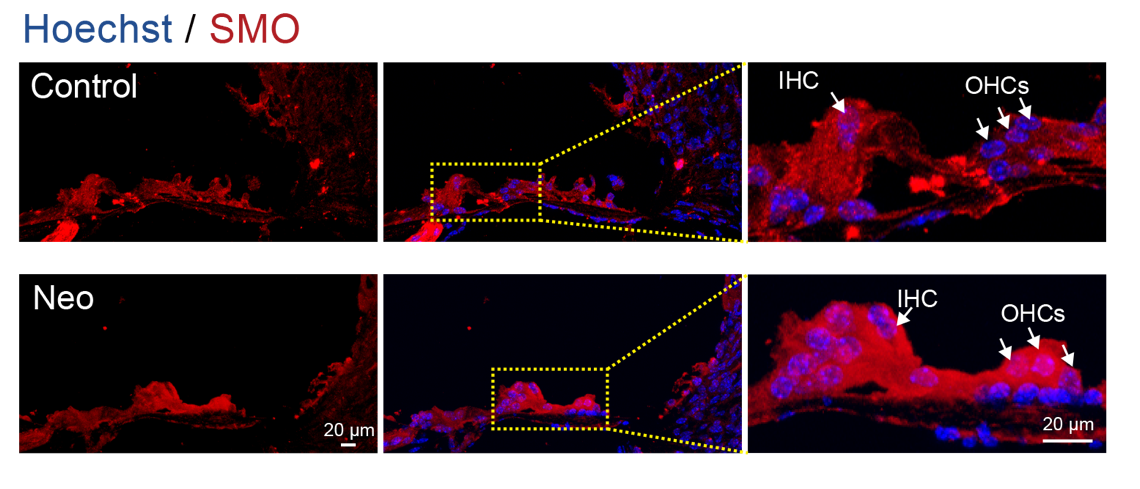
**

**Figure S7A**

**
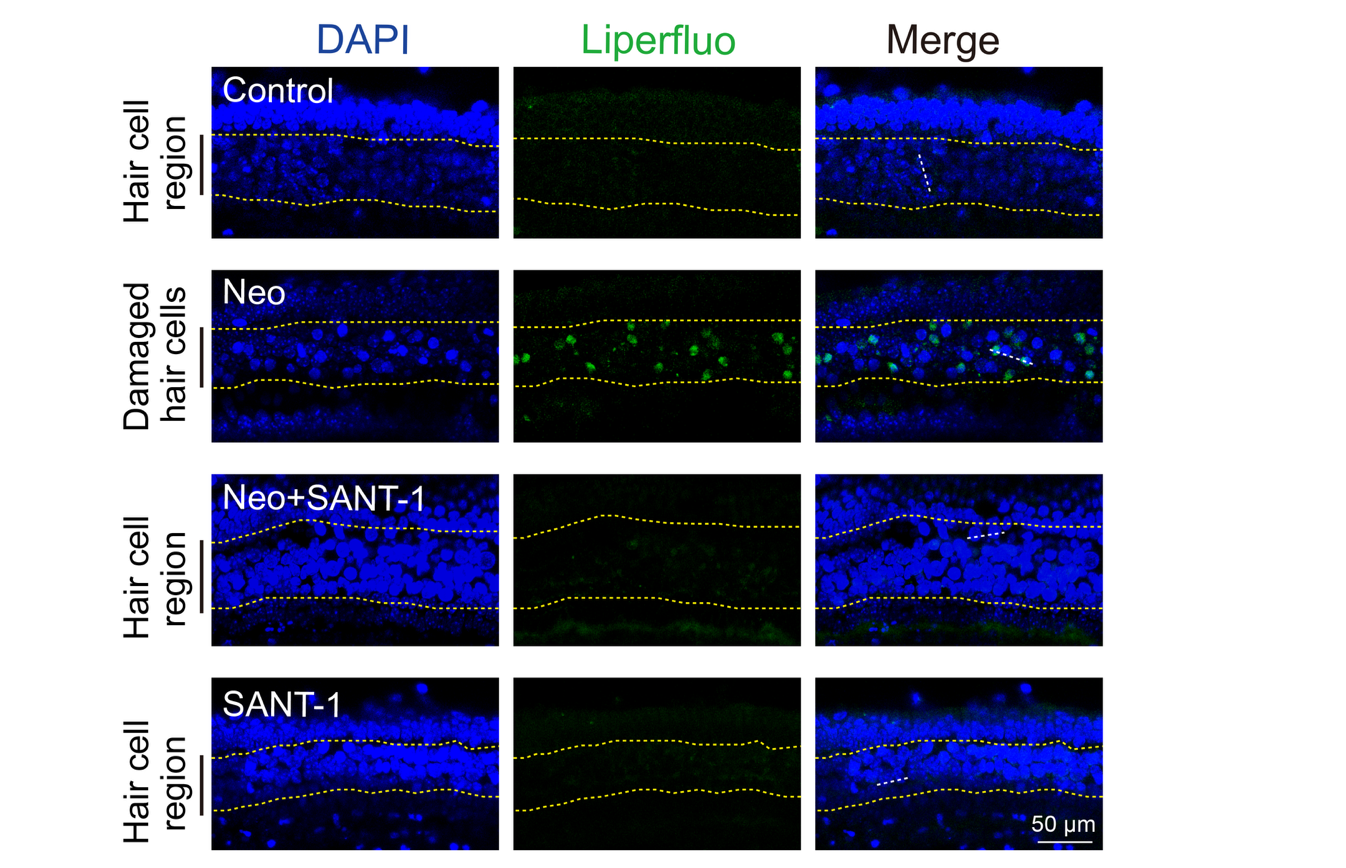
**

**Figure S7C**

**
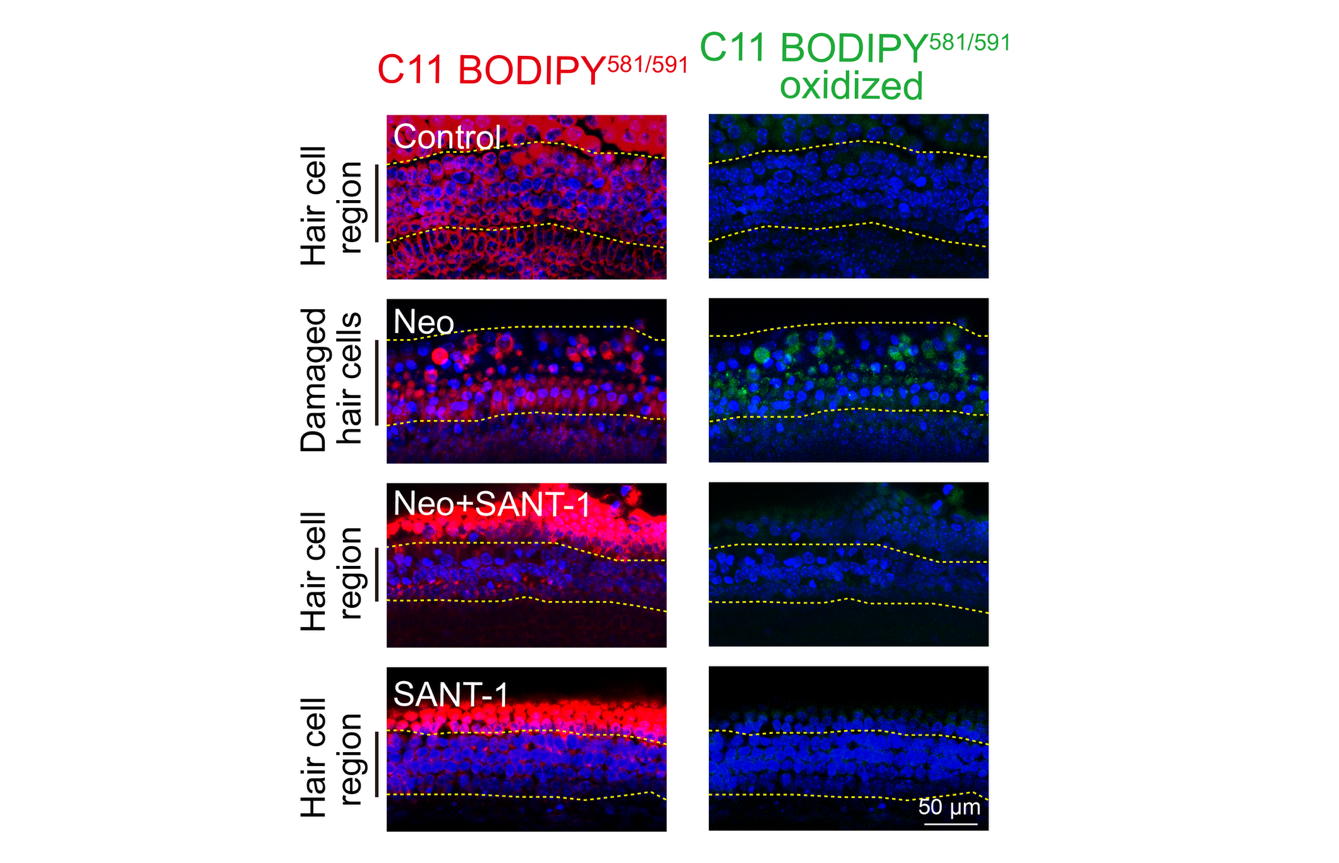
**

**Figure S7E**

**
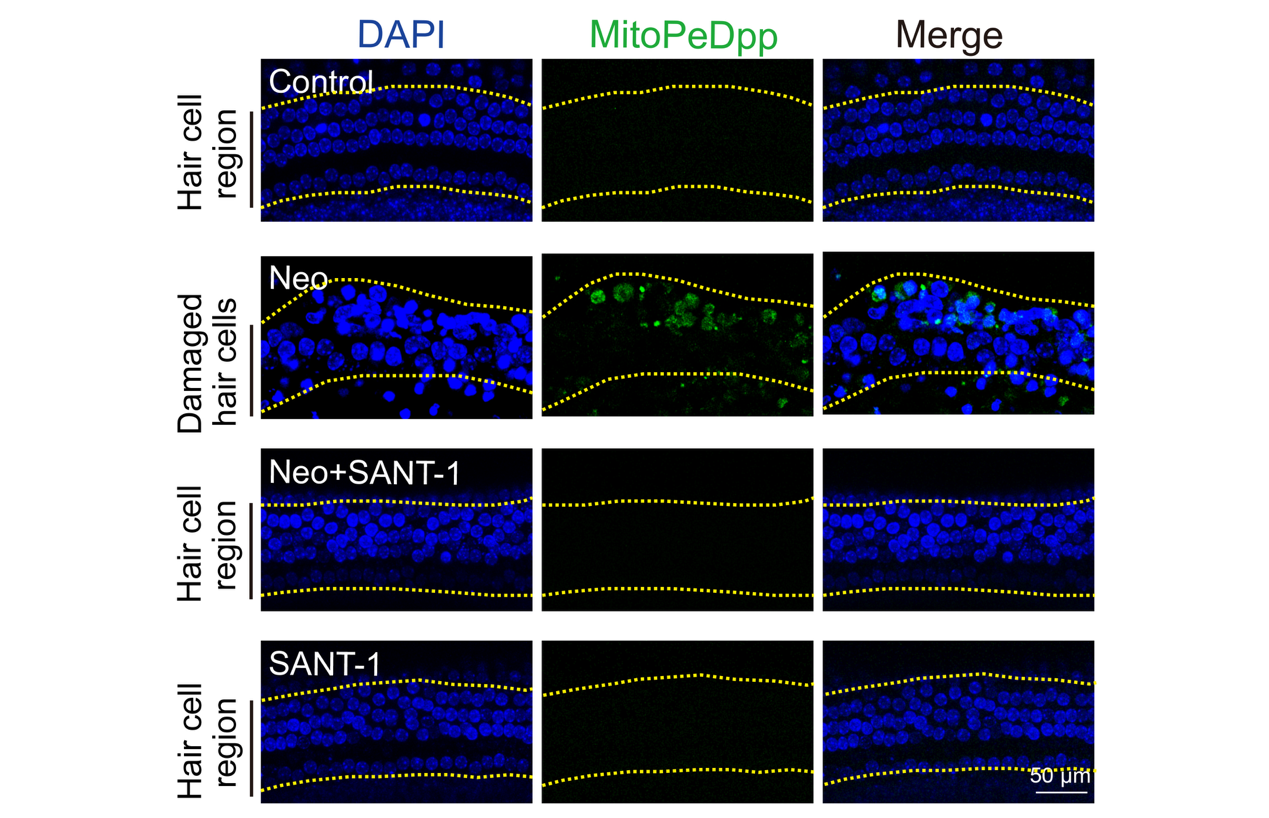
**

**Figure S8**

**
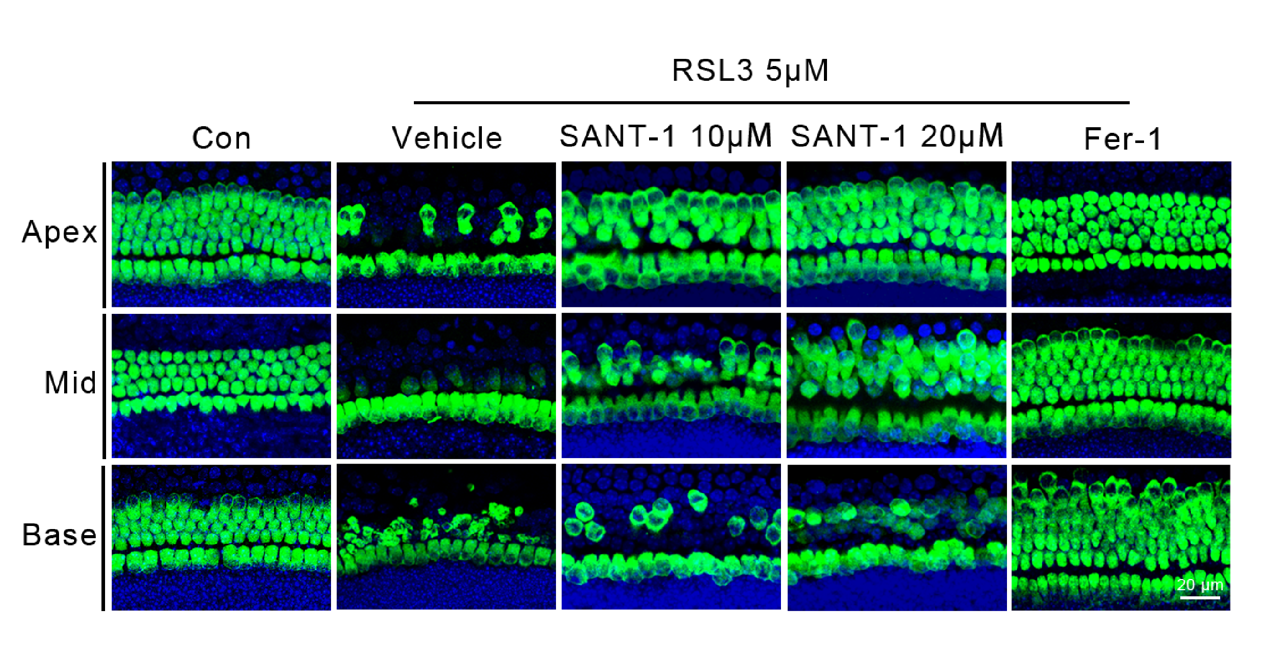
**

**Figure S10A**

**
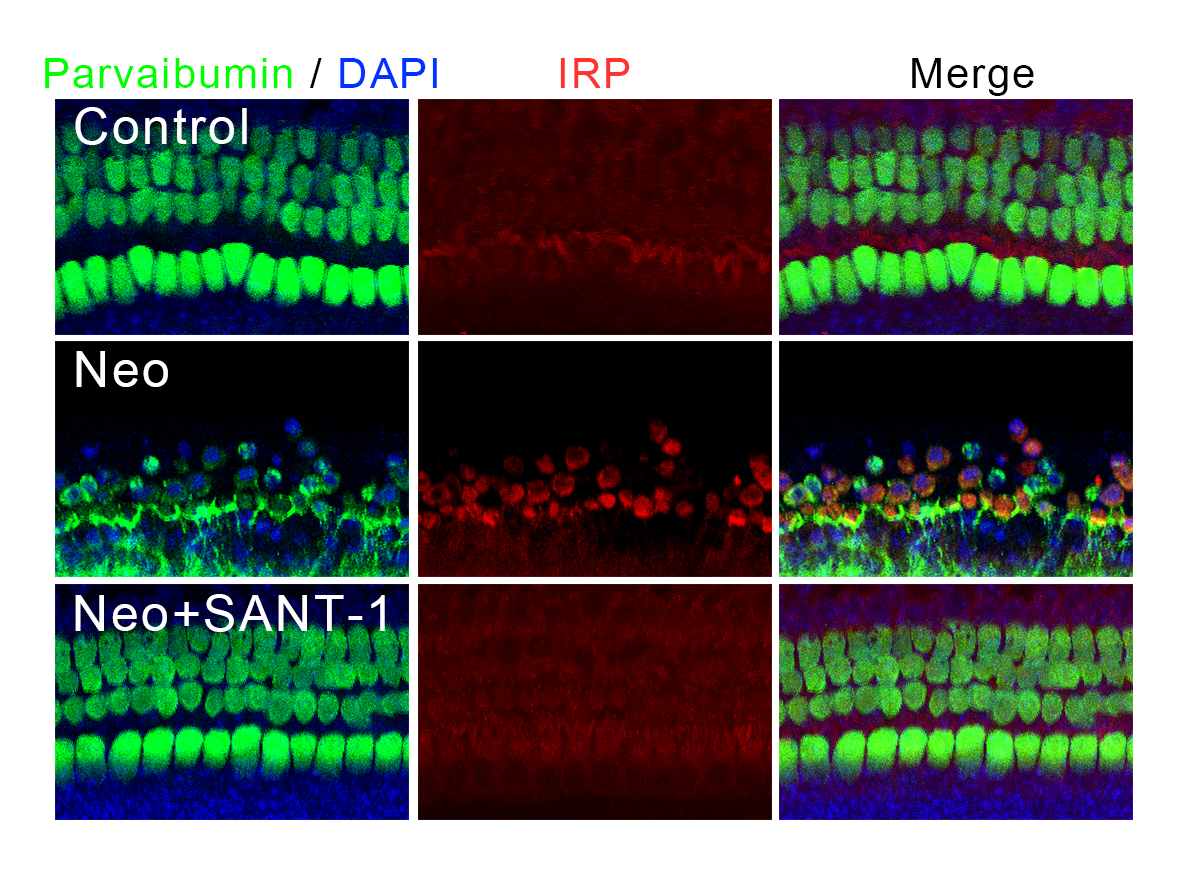
**

**Figure S10B**

**
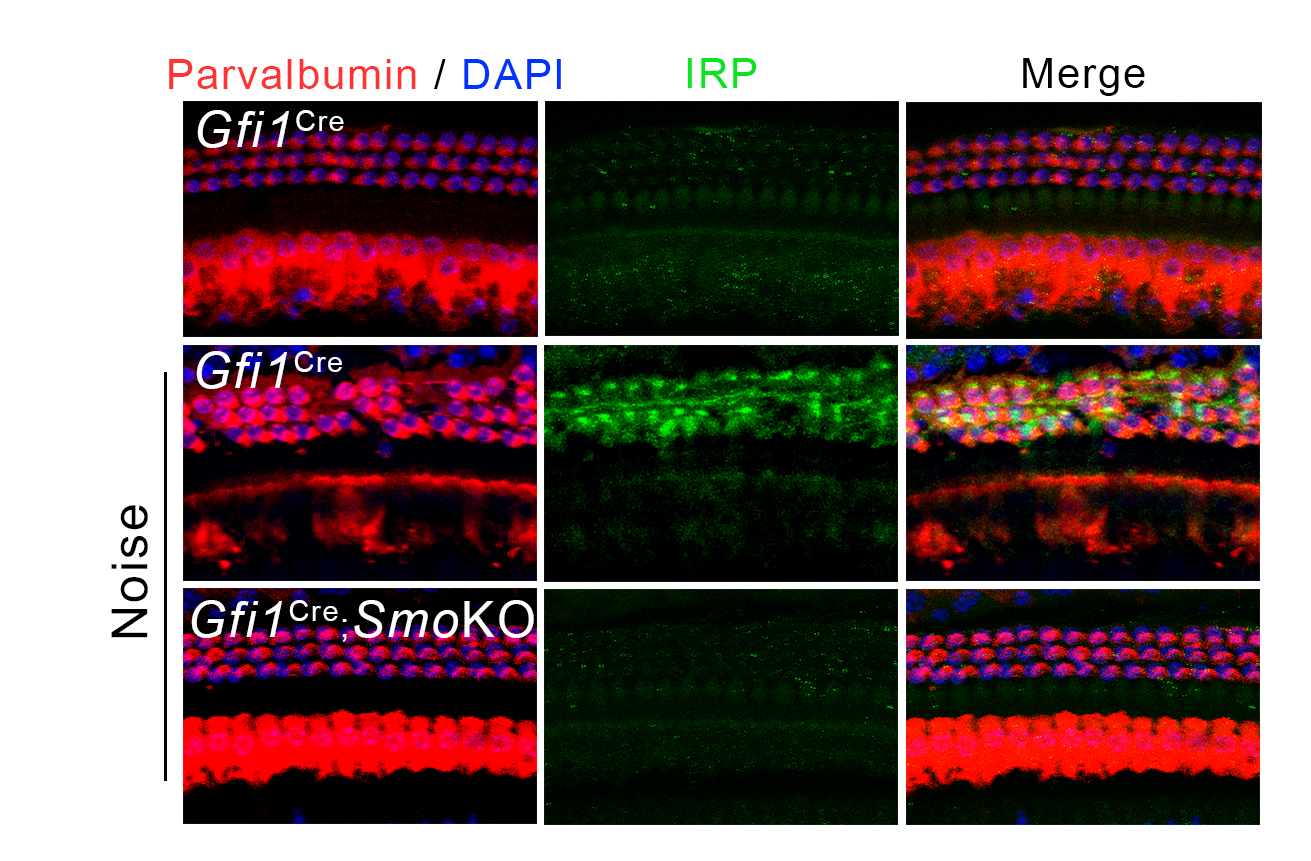
**

**Figure S10C**

**
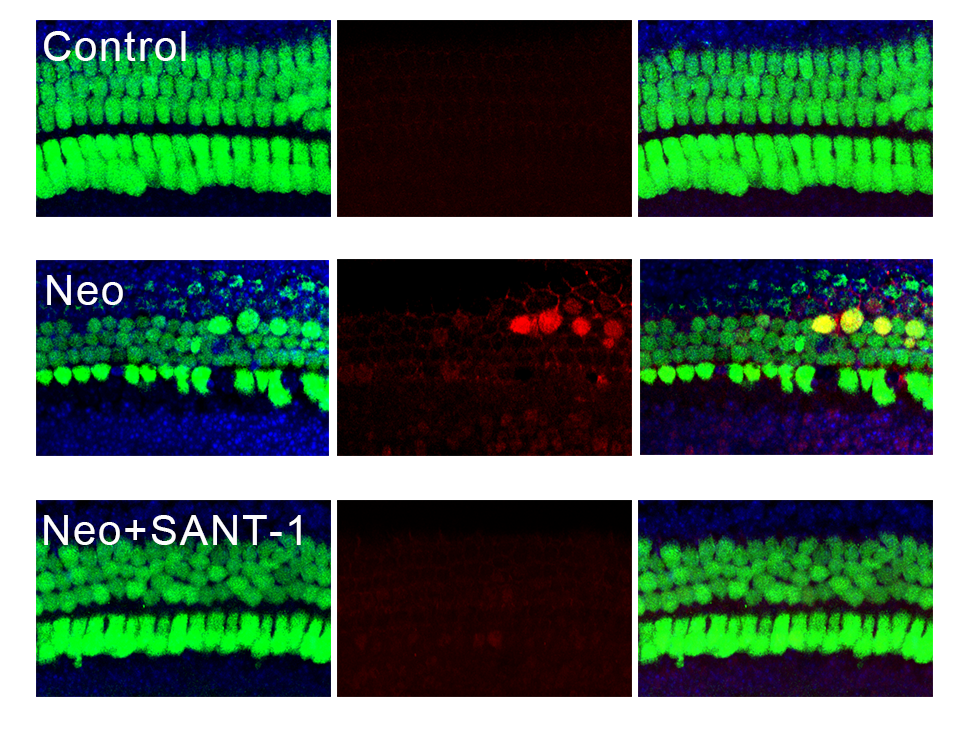
**

**Figure S10D**

**
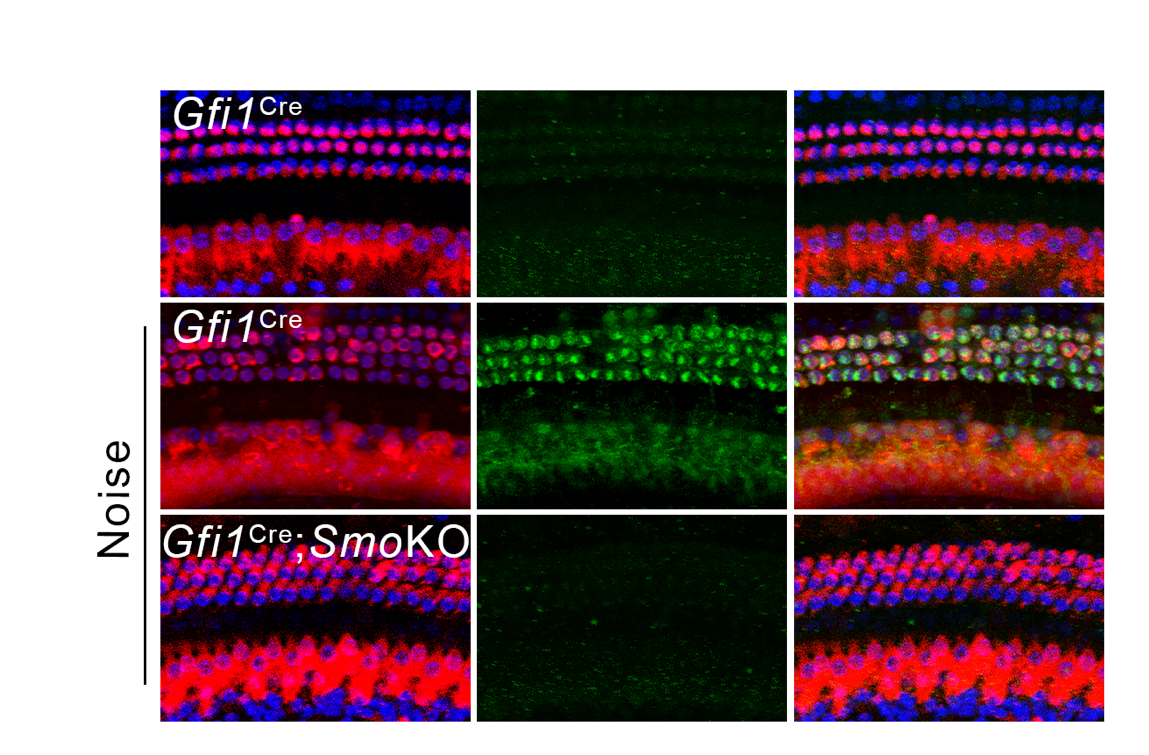
**

**Figure S12**

**
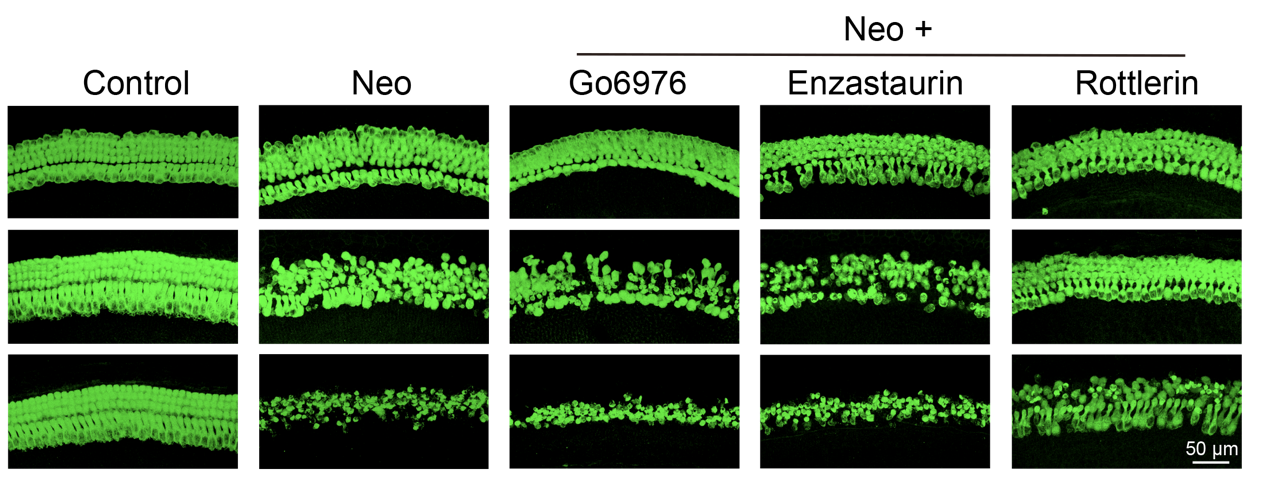
**
